# Design, Synthesis, and Antibacterial Evaluation of Rifampicin–Siderophore Conjugates

**DOI:** 10.1021/acsinfecdis.5c00311

**Published:** 2025-07-05

**Authors:** Vladyslav Lysenko, Mei-Ling Gao, Fabienne A. C. Sterk, Paolo Innocenti, Cornelis J. Slingerland, Nathaniel I. Martin

**Affiliations:** Biological Chemistry Group, Institute of Biology Leiden, 4496 Leiden University, Sylviusweg 72, 2333 BE Leiden, The Netherlands

**Keywords:** antibiotic, resistance, Gram-negative bacteria, rifampicin, siderophore, conjugate, Trojan horse

## Abstract

The growing concern over antibiotic resistance has sparked increased attention toward developing alternative antibiotic strategies. One promising approach, known as the “Trojan horse” strategy, involves the use of siderophores to hijack bacteria’s iron transport systems as a way of delivering antibiotics inside the bacterial cell. This method is particularly promising in tackling Gram-negative bacteria, which have an outer membrane that many antibiotics cannot penetrate. One such antibiotic is rifampicin, a drug used to treat tuberculosis and infections caused by Gram-positive bacteria. Although rifampicin binds to a highly conserved bacterial RNA subunit, its activity is generally poor against Gram-negative bacteria due to their outer membrane. Aiming to expand rifampicin’s efficacy, we here report the design and synthesis of several rifampicin–siderophore conjugates that exhibit enhanced activity against Gram-negative pathogens. Our findings indicate that the structural features of both the linker and catechol are crucial for the activity of conjugates with compound **33**, wherein rifampicin is connected to chlorocatechol via a short ester linker, showing an up to 32-fold improvement in activity.

## Graphical abstract

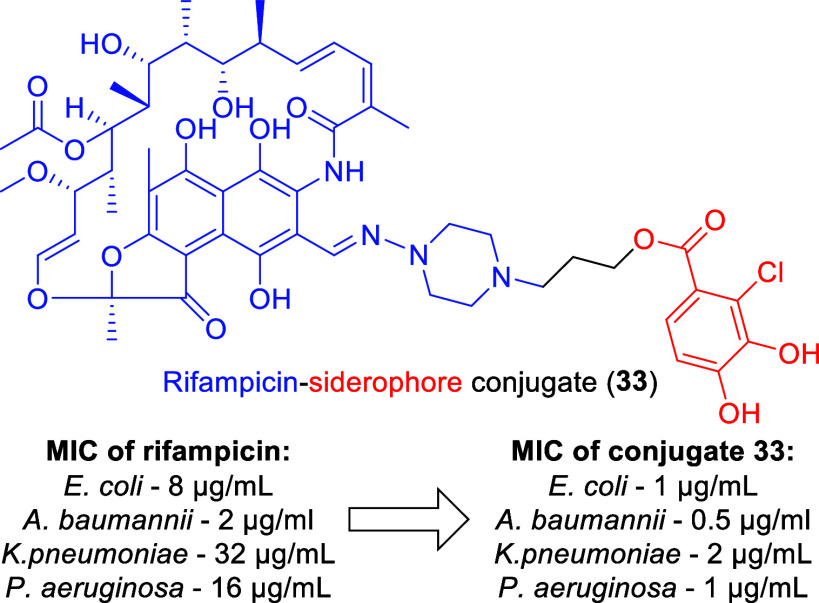

The rapid emergence and global spread of multidrug-resistant (MDR) bacteria represent a significant public health crisis and pose substantial challenges to modern medicine.^1–3^ Of particular concern are MDR Gram-negative pathogens, including , , , and , which are associated with severe and often life-threatening infections such as sepsis, pneumonia, and urinary tract infections.^4^ Due to their impermeable outer membrane (OM) and efficient efflux pumps, Gram-negative bacteria have generally proven to be more challenging to treat with antibiotics than Gram-positive pathogens.^5–7^ The growing threat posed by infections due to MDR Gram-negative bacteria underscores the need for novel antibiotics that can overcome both their intrinsic and acquired resistance mechanisms.

In this study, we describe approaches to expanding the therapeutic utility of rifampicin by making it more active toward Gram-negative bacteria. Rifampicin (Figure 1A) is a potent inhibitor of bacterial RNA polymerase and is a cornerstone antibiotic for treating infections and other diseases caused by Gram-positive bacteria.^8–11^ While rifampicin exhibits similar inhibitory activity against RNA polymerase from both Gram-positive and Gram-negative bacteria, its clinical efficacy against Gram-negative bacteria is severely limited due to poor outer membrane permeability.^12,13^ Despite these limitations, rifampicin’s well-characterized mechanism of action makes it an attractive candidate for modifications aimed at overcoming its lack of OM permeability against Gram-negative pathogens. Furthermore, recent studies have shown that when used together with agents capable of inducing OM disruption, the antimicrobial activity of rifampicin is improved, likely due to increased access to its intracellular target.^14,15^

**Figure 1:**
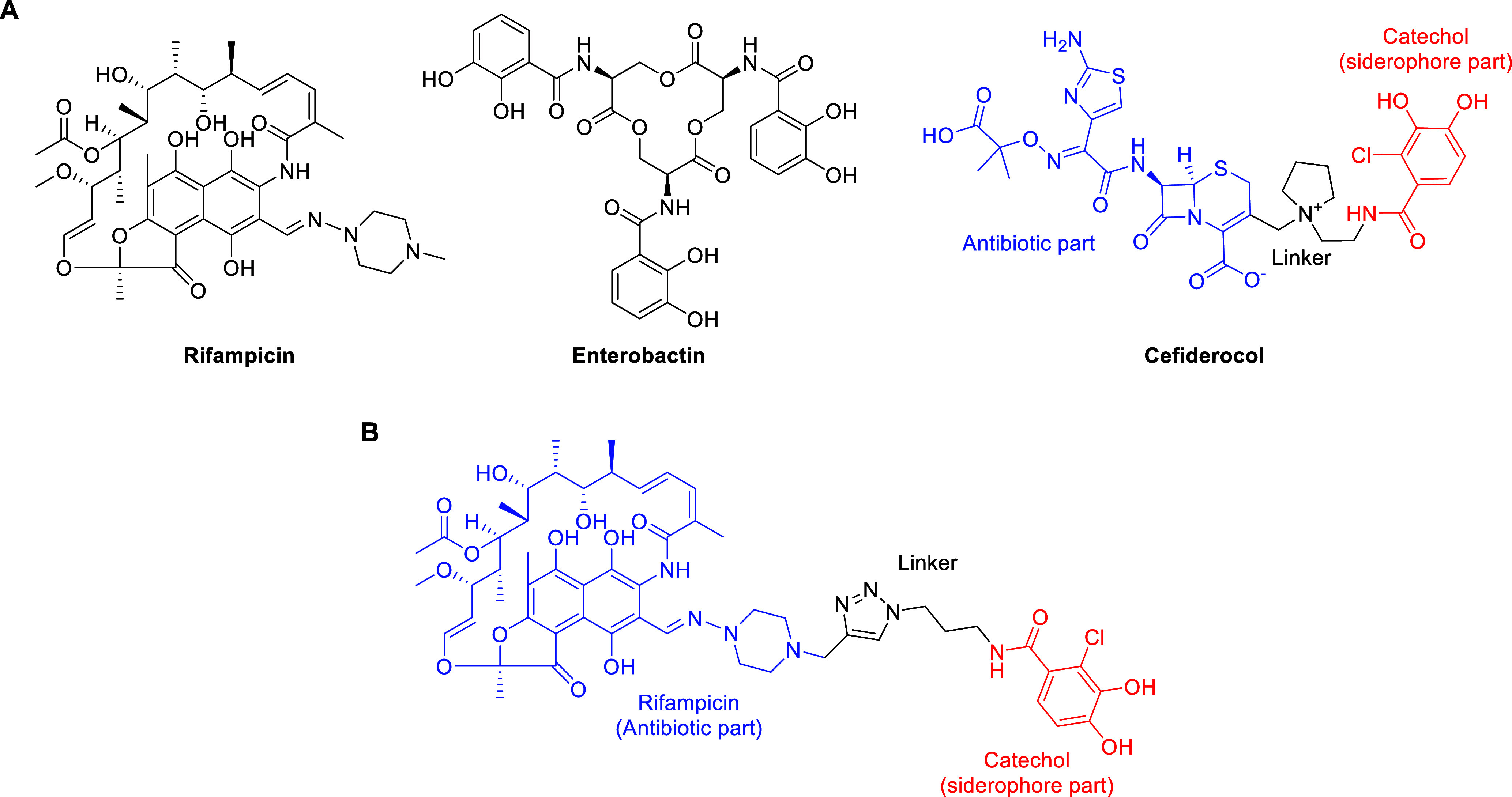
(A) Structures of rifampicin, enterobactin and cefiderocol. (B) Prototype rifampicin–siderophore conjugate (**1**).

To enable rifampicin to breach the OM of Gram-negative bacteria as a means of enhancing its antibiotic efficacy, we opted to explore the so-called “Trojan horse” strategy. This approach involves exploiting the bacterial iron acquisition pathway, which is critical for bacterial growth and pathogenicity.^16–18^ To acquire iron from environments where this critical resource is scarce, such as in mammalian hosts, bacteria synthesize and secrete siderophores that tightly bind exogenous iron and facilitate its uptake through specific outer membrane transporters.^19–21^ Enterobactin (Figure 1A) is the preeminent naturally produced siderophore. Enterobactin’s highly efficient iron binding is driven by the specific shape of its macrocycle and the positioning of the catechol rings, which induce the formation of a stable ferric complex.^22,23^ The underlying idea of the ″Trojan horse″ strategy is to covalently link a siderophore-like moiety with an antibiotic that has poor OM permeability. This then allows for hijacking of the bacterial iron uptake system to transport the siderophore–antibiotic conjugate across the OM with subsequent release inside the cell.^17,24,25^ Previous studies have shown that some rifamycin–siderophore conjugates bearing cleavable linkers can be transported inside bacteria, after which the rifamycin payload is released.^26^ Also of note, analogs of rifampicin, such as rifabutin, were also found to overcome the membrane permeability issue by hijacking an iron transporter to get inside the bacteria.^27–30^ These findings suggest that rifampicin and its analogues may be particularly well suited to Trojan horse strategies as a means of enhancing their activity against Gram-negative bacteria.

A prime example of an antibiotic that exploits the “Trojan horse” approach is the FDA-approved drug cefiderocol, which consists of a β-lactam core conjugated to a chlorocatechol moiety (Figure 1A). The chlorocatechol group significantly enhances the activity of cefiderocol compared to other β-lactam antibiotics and positions it as one of the few options for treating multidrug-resistant Gram-negative pathogens.^31–33^ Building from this concept, we envisioned conjugating rifampicin to a similar chlorocatechol unit (Figure 1B) as a means of enhancing antibacterial activity against Gram-negative bacteria. Previous reports have identified the piperazine ring of rifampicin as an exploitable site for chemical modification, given that it is not directly involved in binding to the RNA polymerase active site.^34,35^ This is an important consideration given the need to ensure that the conjugate preserves rifampicin’s inhibitory function while permitting structural modifications that facilitate siderophore-mediated uptake. Here, we report the synthesis and evaluation of a series of rifampicin–siderophore conjugates incorporating linkers with different properties and lengths. By optimizing the linker, we successfully identified a subset of conjugates with enhanced antibacterial activity and assessed the contribution of the bacterial iron uptake system to this activity.

## Results and Discussion

In designing a prototype conjugate of rifampicin and the chlorocatechol unit derived from cefiderocol, we elected to first apply an azide–alkyne “click-chemistry” ligation strategy to provide convenient access to conjugate **1** via well-described methods.^36–39^ The key building blocks needed for our strategy were the previously reported rifampicin alkyne **2**,^39,40^ and catechol-azide **7** (Scheme 1). In doing so, azido amine **4** was first generated by azide displacement of the corresponding 3-chloro propylamine. Carboxylic acid **6** was then prepared via the BBr_3_-mediated demethylation of commercially available **5** and coupled to **4** to yield catechol-azide **7**. Conventional click chemistry conditions were then used to ligate alkyne **2** (prepared as in Scheme S1) and azide **7**, yielding conjugate **1**. In addition to conjugate **1**, compounds **8−12** were also prepared as a series of controls to allow for the assessment of the contributions of the triazole and chlorocatechol moieties (Scheme S2) relative to unmodified rifampicin.

**Scheme 1:**
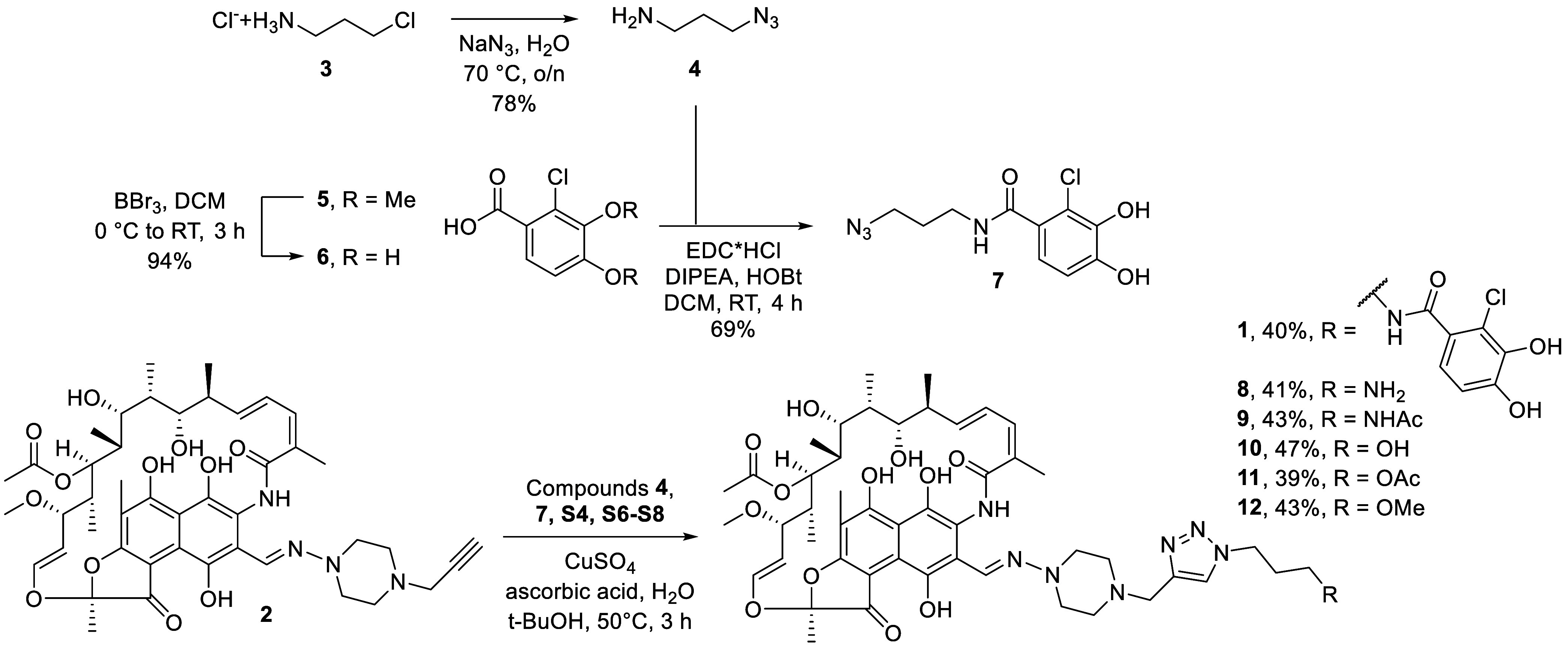
Synthesis of the Conjugates using the Click-Chemistry Approach

The activity of conjugate **1** was then assessed against a panel of Gram-negative bacteria (Table 1). Minimum inhibitory concentration (MIC) values were determined using standard broth dilution assays and compared to those measured for authentic rifampicin. Given that the impact of siderophore-mediated transport is most effective at low iron concentrations,^41^ the MIC assays were run using iron-depleted, cation-adjusted Mueller–Hinton broth (ID-CAMHB) as the growth medium. Rather unexpectedly, the results of these MIC assays showed that the antibacterial activity of conjugate **1** was consistently lower than that of unmodified rifampicin. Activity was reduced 8-fold against ATCC 25922 and 16-fold against ATCC 19606. Furthermore, **1** showed no measurable activity against ATCC 13883 and ATCC 10145. Only against BW 25113 was the activity of compound **1** equivalent to that of rifampicin. Given these findings, we proceeded to test compound **1** against two well-characterized OM-disrupted mutants: JW 3594 Δ*rfaD* (with the gene responsible for LPS biosynthesis knocked out) and BW 25113 Δ*bamB* Δ*tolC* (with deletions of the bamB gene alongside knockout of the tolC porin gene).^42,43^ These assays were conducted to assess whether **1** shows activity against hypersensitive strains in which the OM is compromised. We also included compound **2** to assess whether the alkyne substituent has any effect on rifampicin’s activity (Table 2). As expected, we found that rifampicin exhibits activity against the membrane-disrupted strains. Conversely, we observed that conjugate **1** again displayed reduced activity against both strains, with the largest reduction (8-fold) against the Δ*rfaD* strain. In comparison, compound **2** was found to have similar MIC values to rifampicin, with no significant reduction in activity. These findings suggested that the triazole-based linker strategy used in conjugate **1** may result in reduced binding to the target RNA polymerase. To investigate whether the introduction of the triazole was the key factor in the conjugate’s diminished antimicrobial potency, we therefore prepared and tested a series of triazole-modified rifampicin analogs terminating in amine, alcohol, amide, ester, or ether moieties (compounds **8**–**12**, Scheme 1) to estimate a possibility of modifying rifampicin via click-chemistry conjugation approach without affecting its activity. These compounds were tested against wild-type BW 25113, membrane-disrupted JW 3594 Δ*rfaD*, and BW 25113 Δ*bamB* Δ*tolC*. In all cases, the activities of the triazole-bearing compounds were reduced relative to rifampicin (Table 2), further indicating that triazole-based linkers, which include the use of building block **2**, negatively impact antibacterial potency.

**Table 1:**
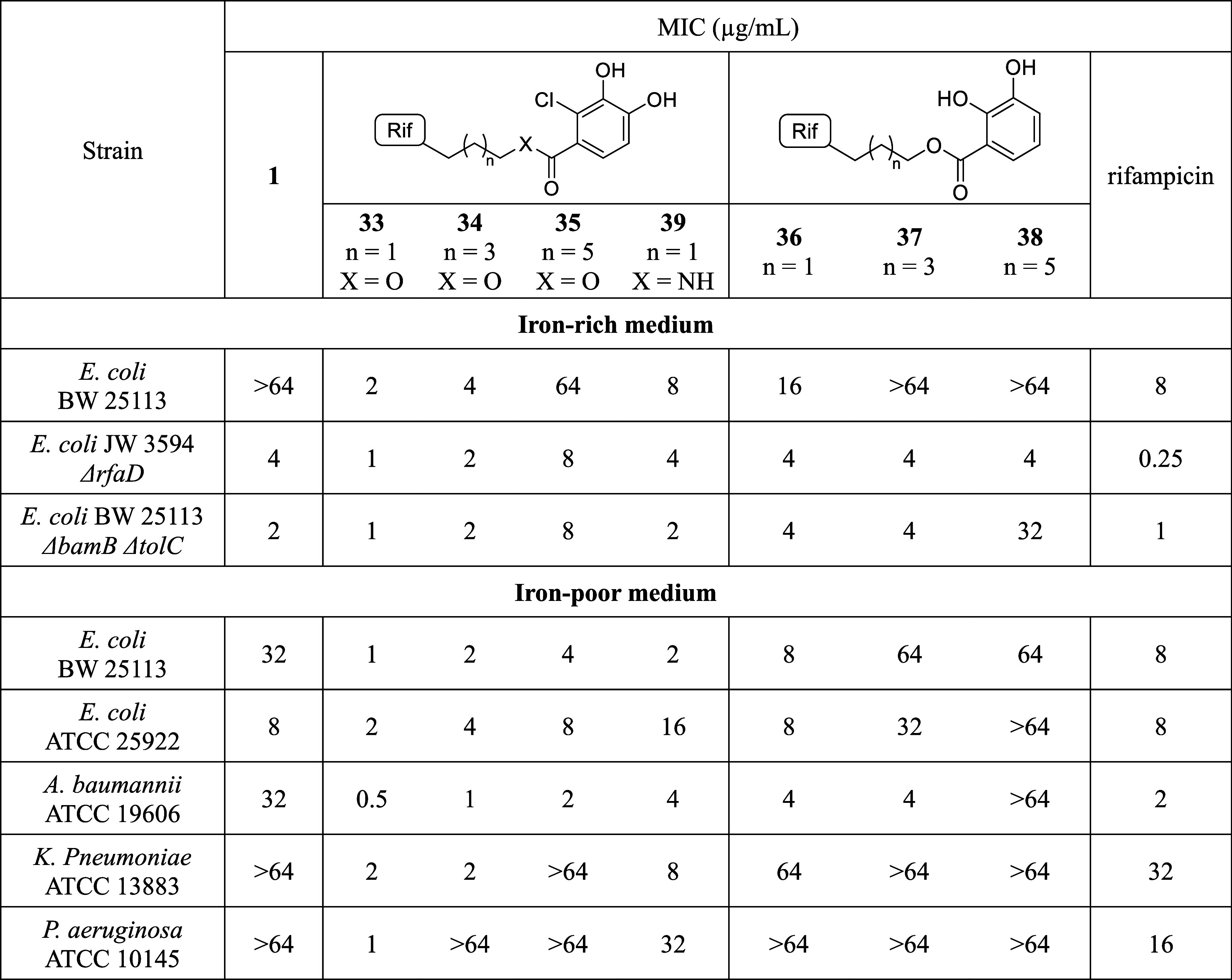
MICs of Compounds **1**, **33-39**, and Rifampicin in CAMHB (Iron–Rich Medium) and ID-CAMHB (Iron–Poor Medium) Against a Panel of Gram-Negative Bacteria^a^

a For the structure of conjugate **1** see Figure 1.

**Table 2:**
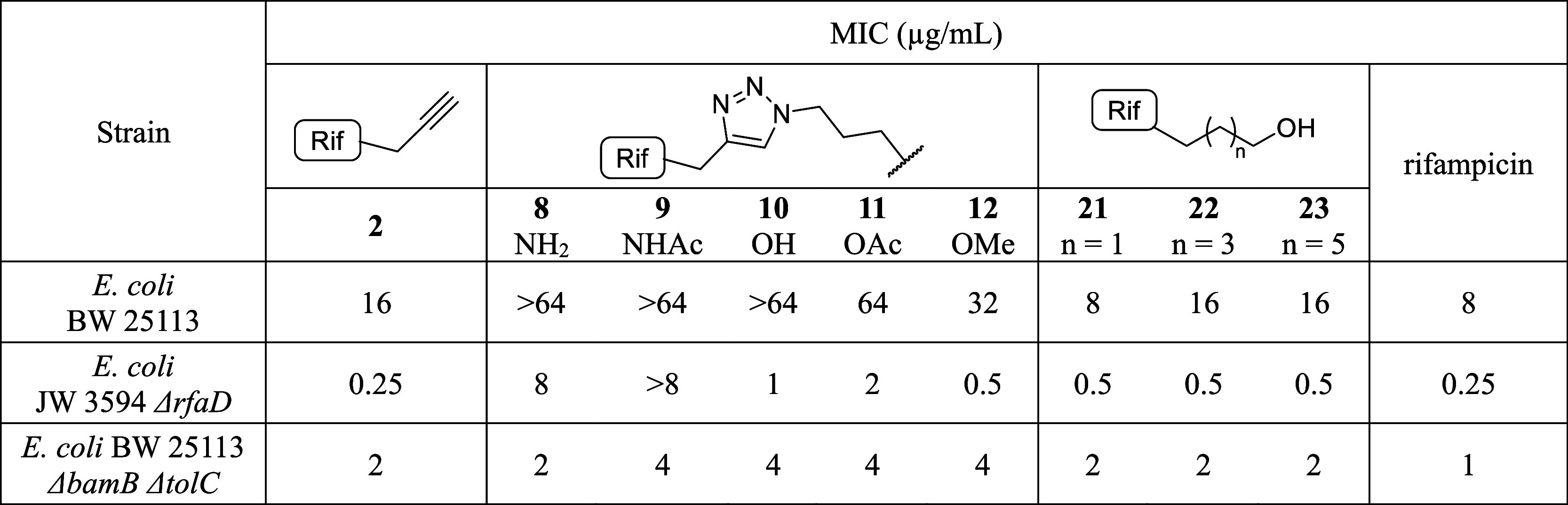
MICs of Compounds **2**, **8**–**12**, **21**–**23** Compared to Rifampicin Against Wild-Type and Membrane-Deficient Δ*rfaD* and Δ*bamB* Δ*tolC* Strains in CAMHB (Iron−Rich Medium)

In light of these findings, we proceeded to explore alternative linking strategies not dependent on alkyne–azide ligation. To this end, we designed a series of ester-linked conjugates wherein the rifampicin piperazine was substituted with an alkyl linker, terminating with an alcohol group to which the catechol carboxylic acid would be coupled. To accommodate variations in the linker, we designed a synthetic route that would allow for the most divergent approach (Scheme 2). Starting from Boc-protected piperazine **13**, reaction with *tert*-butyl nitrite yielded *N*-nitroso compound **14** quantitatively. Reduction using zinc with NH_4_Cl afforded hydrazine **15**, which was subsequently protected with a Cbz group, resulting in piperazine **16**. Treatment with TFA in DCM, followed by evaporation and trituration, afforded compound **17** as a TFA salt in multigram quantities. Protected piperazine **17** was then alkylated with three different bromo alcohols of varying lengths to give building blocks **18**–**20** in good yields. Following the removal of the Cbz group by hydrogenation, the piperazines were condensed with the commercially available rifaldehyde to yield compounds **21**–**23**. Before coupling **21**–**23** with the catechol carboxylic acid, we tested their activities against a panel of bacteria to assess the impact of the linkers. The results of these assays were encouraging, with substituted rifampicin building blocks **21**–**23** showing activity similar to that of the parent antibiotic (Table 2). Encouraged by these findings, we proceeded to generate the corresponding rifampicin–siderophore conjugates connected by an ester linkage.

**Scheme 2:**
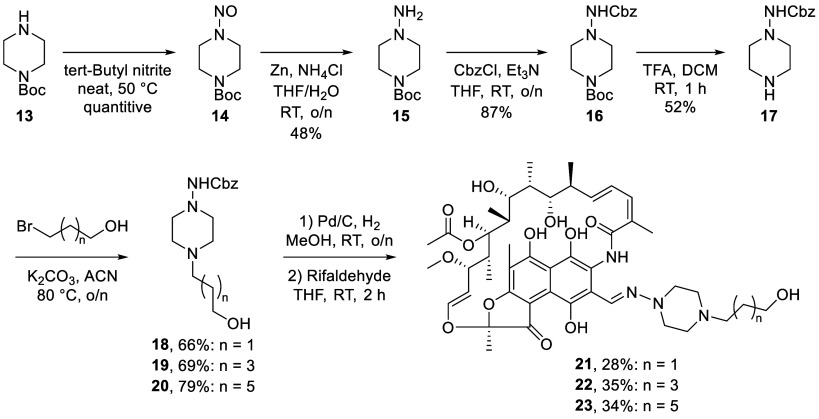
Synthesis of Rifampicin Analogues Containing Different Alcohol Linkers

Two different catechol fragments were chosen as building blocks for the ester-linked rifampicin conjugates: the chlorocatechol featured in compound **1** (derived from the structure of cefiderocol) and a simpler nonchlorinated variant inspired by the catechol unit of enterobactin. In doing so, we generated a set of six rifampicin–siderophore conjugates consisting of alkyl linkers of three different lengths and two different catechol moieties (Scheme 3). The protected catechol building blocks required for our synthetic strategy were prepared from compounds **6** and **24** via reaction with benzyl bromide in acetone, followed by aqueous basic hydrolysis to obtain the benzoic acid derivatives **25** and **26**. Using DIC/DMAP activation, **25** and **26** were then coupled to the previously obtained Cbz-protected aminopiperazine-based alcohols **18**–**20** to give the protected ester intermediates. Following the workup, the protected ester products were directly treated with Pd/C under a hydrogen atmosphere to simultaneously remove the Cbz and benzyl groups. This gave building blocks **27**–**32**, which were then condensed with rifaldehyde to yield rifampicin–siderophore conjugates **33**–**38**. The antibacterial activities of **33–38** were then evaluated against the same panel of strains previously used for compound **1** and again in iron–poor medium (Table 1).

**Scheme 3:**
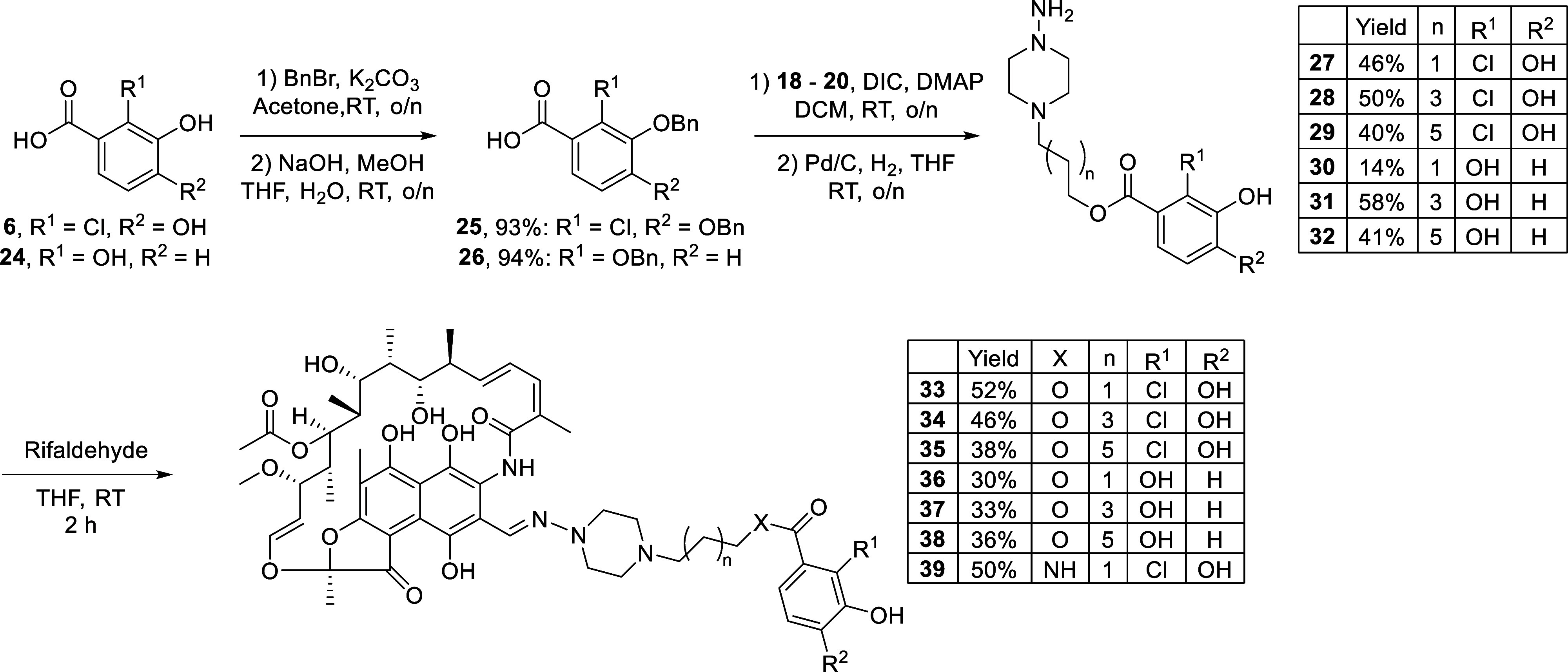
Synthesis of the Rifampicin–Siderophore Conjugates

We were pleased to find that some of the ester-linked conjugates demonstrated an antimicrobial profile equivalent to or better than that of rifampicin itself, with compound **33**, bearing the three-carbon linker and chlorocatechol moiety, being the most potent overall. Conjugates **33–38** were also found to be nonhemolytic, indicating that the enhanced antibacterial activities observed are not due to nonspecific membrane disruption (Figure S1). Additionally, in MIC assays using membrane-deficient strains, **33** showed activity comparable to that of rifampicin (Table 1). This finding suggests that the ester linkage does not interfere with target binding as much as the triazole-based linker used in the initial design of conjugate **1**. The most considerable increase in potency observed for compound **33** relative to rifampicin (16-fold) was found against strains of and , whose proliferation was interestingly not at all affected by treatment with compound **1** (Table 1). Upon comparing the activity of the rifampicin–siderophore conjugates, we noticed a number of interesting correlations. First, conjugates deriving from chlorocatechol **6** appeared more potent than those derived from catechol **24**. For example, when comparing compound **36** to compound **33**, which differ only in the substitution pattern on the bisphenol ring, we noticed a 4- to 64-fold difference in the MICs, suggesting that the chloro-containing catechol derived from cefiderocol has a beneficial effect on the activity compared to the simpler catechol derived from enterobactin. A similar trend, favoring the activity of chloro-substituted conjugates, was observed for other compounds with longer linkers. Additionally, we found that the distance between the rifampicin core and the catechol portion has an impact on the antimicrobial potency. The data summarized in Table 1 show that analog **33** is more potent than **34**, which in turn is more active than **35**. This trend, also shared by conjugates **36**–**38**, incorporating the catechol derived from enterobactin, suggests a preference for the shorter linker.

Given that conjugate **33** contains an ester linkage between rifampicin and the chlorocatechol moiety, we elected to also synthesize the corresponding amide analog **39** to assess whether the more stable and synthetically convenient amide linked variant is also more active than rifampicin. The synthesis of **39** was carried out following the same route depicted in Scheme 3, but employing the amide-linked intermediate **S12** in the final condensation with rifaldehyde (**S12** was prepared in five steps from Cbz-protected *N*-aminopyperazine **17**, as illustrated in Scheme S3). Interestingly, when tested against the same panel of Gram-negative bacteria, conjugate **39** was found to have consistently reduced antibacterial activity relative to both ester-linked **33** and rifamipcin. This finding may provide insights into the working mechanism of these conjugates. In the case of **33**, siderophore-mediated entry into the bacterial cell might be followed by the hydrolysis of the ester linkage to release active compound **21** (Table 2). In contrast, the same pathway is less likely with **39**, due to the greater hydrolytic stability of the amide linkage. However, based on the data presented, it cannot be conclusively stated that the decreased potency observed for **39** is due to linker stability. Other possible effects, such as reduced uptake of **39** by outer membrane transporters, or decreased binding to the target RNA polymerase could also explain the reduced activity observed.

Given the strongly enhanced activity of conjugate **33** relative to rifampicin when tested against strains used in the preliminary screen, the compound was further evaluated against a broader panel of clinical isolates with different resistance mechanisms (Table 3). Again, **33** displayed enhanced activity against almost all strains compared to rifampicin. While testing against a panel of different strains, the activity enhancement over rifampicin was from 4- to 32-fold, with the biggest increase of activity against the 2018–022 (VIM-2) strain. Meanwhile, good activity, with MICs ranging from 0.5 to 2 μg/mL, was also observed for a number of strains, including multidrug-resistant isolates, which are among the most clinically challenging classes of Gram-negative pathogens.^44,45^ These MIC values correspond to a 2–8-fold improved activity over rifampicin, depending on the strain. Compound **33** also showed enhanced potency against multiple resistant strains, resulting in 4- to 16-fold lower MIC values than those measured for rifampicin. On the other hand, a number of the strains tested were not found to be more sensitive to conjugate **33**, showing similar MIC values as those of rifampicin, with the exception of NRZ-03961, where we observed a 16-fold increase in potency, similar to our findings with ATCC 10145 in the initial screen. The activity of conjugate **33** was also improved relative to rifampicin against highly colistin-resistant strains of pRIVM_C029515_2 and RIVM_C019741 acquired from hospitals in The Netherlands.^46^ Interestingly, the RC0089 and NCTC 13443 strains, both bearing NDM-1 resistance were found to be resistant to both rifampicin and conjugate **33**. This is possible related to the finding that plasmids that harbor the NDM-1 gene are often associated with other resistance markers, including those conferring rifampicin-resistance.^47^

**Table 3:** MICs of Conjugate **33** and Rifampicin in ID-CAMHB (Iron–Poor Medium) Against Multiple Bacterial Strains, Including Drug-Resistant Clinical Isolates

	MIC (μg/mL)	
strain	**33**	rifampicin
*E. coli* BW 25113	1	8
*E. coli* ATCC 25922	2	8
*E. coli* NTCT 13846 (MDR)	2	16
*E. coli* MVAST0072 (MDR)	4	16
*E. coli* EQAS 2016 (mcr-1)	4	16
*E. coli* pRIVM_C029515_2 (mcr-1)	4	16
*E. coli*2018–022 (VIM-2)	1	32
*E. coli* RC0089 (NDM-1)	>32	>64
*A. baumannii* ATCC 19606	0.5	2
*A. baumannii* ATCC 17978	1	8
*A. baumannii* NRZ-00687 (NDM-2)	1	4
*A. baumannii* RUH-134 (MDR)	2	8
*A. baumannii* 2018–006 (NDM/OXA-023/OXA-051)	2	4
*A. baumannii* KML-11668 (MDR)	1	2
*K. pneumoniae* ATCC 13883	2	32
*K. pneumoniae* NR-48977 (MDR)	4	64
*K. pneumoniae* NR-48978 (MDR)	4	64
*K. pneumoniae* RIVM_C019741 (colistin-resistant)	8	64
*K. pneumoniae* 1124 (VIM-1)	8	32
*K. pneumoniae* NCTC 13443 (NDM-1)	>32	>64
*P. aeruginosa* ATCC 10145	1	16
*P. aeruginosa* ATCC 27853	8	16
*P. aeruginosa* PAO1	16	16
*P. aeruginosa* NRZ-08418 (NDM-1)	16	16
*P. aeruginosa* 1427 (VIM-2)	8	8
*P. aeruginosa* NRZ-03961 (IMP-1)	0.5	8

The role of available iron and iron transport on the activity of the rifampicin–siderophore conjugates was further investigated. In doing so, we directly compared the antibacterial activities of **33–39** in iron-rich and iron-depleted media (Table 1). In all cases, the MIC values measured for our conjugates were heightened when tested in iron-rich media, suggesting that compounds are iron-dependent antibiotics, unlike rifampicin, which showed the same MIC values in both iron-rich and iron-depleted media. To further investigate the contribution of the siderophore moiety and its role in exploiting the bacterial iron uptake system, we assessed the activity of **33** in the presence and absence of the natural siderophore enterobactin (prepared following a previously published procedure with slight modifications, as illustrated in Scheme S4).^48–51^ Under these conditions, we tested several strains, including wild-type BW 25113 as well as knockout strains Δ*entA* (impaired enterobactin biosynthesis), Δ*entC* (impaired enterobactin biosynthesis), and Δ*fepA* (impaired enterobactin import), and a standard ATCC 19606 strain (Table 4). In all cases, enterobactin supplementation reduced the activity of compound **33**. These results are in line with similar competition experiments performed for other siderophore conjugates and suggest that the uptake is likely mediated via a siderophore uptake route.^52–54^ In the presence of enterobactin, conjugate **33** showed up to a 16-fold decrease in activity, while the potency of rifampicin remained unaffected. Taken together, these findings suggest that conjugate **33** operates by hijacking the bacterial cell’s iron transport system to gain entry to the cell, after which the rifampicin moiety can engage with its target, eliciting its antibacterial effect. We also attempted to identify the specific transporter related to the increased activity of conjugate **33**. To do so, we tested **33** alongside rifampicin against a number of single knockout mutants, with impaired enterobactin (catecholate-type siderophore) transport (Δ*fepA,* Δ*fepB,* Δ*fepD*) or impaired ferrichrome (hydroxamate-type siderophore) transport *(*Δ*fhuA,* Δ*fhuB,* Δ*fhuC,* Δ*fhuD,* Δ*fhuE,* Δ*fhuF*).^55,56^ The data obtained (Table S1) show no change in MIC for either compound **33** or rifampicin, when compared to the data against wild-type , suggesting that none of the transporters here assessed is singularly responsible for the increased activity of the conjugate **33**.

**Table 4:** MICs of Compound **33** and Rifampicin in ID-CAMHB (Iron–Poor Medium) With and Without Enterobactin Supplementation

	MIC (μg/mL)			
	**33**		rifampicin	
strain	without enterobactin	+8 μg/mL enterobactin	without enterobactin	+8 μg/mL enterobactin
E. coli BW 25113	2	16	8	8
E. coli Δ*entA*	2	16	8	8
E. coli Δ*entC*	1	16	8	8
E. coli Δ*fepA*	2	8	8	8
A. baumannii ATCC 19606	0.5	4	2	2

In conclusion, we here report the development of a series of rifampicin–siderophore conjugates with activity against Gram-negative bacteria, which are normally impervious to the parent antibiotic. Our findings demonstrate that the activity of the conjugates is heavily dependent on the nature and length of the linkers used in conjugating rifampicin with established catechol-based iron-binding moieties. Notably, while the application of alkyne/azide click-chemistry successfully enabled the construction of a first-generation rifampicin–siderophore conjugate, it was found to be devoid of activity, an effect subsequently attributed to the presence of the resulting triazole moiety. As an alternative, we found that rifampicin analogs prepared via ester conjugation showed improved activity compared to the parent antibiotic. Control experiments carried out with the natural siderophore enterobactin, as well as with iron-poor and iron-rich media, suggest that these conjugates exploit the bacterial cell’s iron transport system to penetrate the OM. These findings serve to further inform the design of antibiotics aimed at leveraging bacteria’s dependence on iron uptake pathways. Moreover, the synthetic routes developed for the preparation of rifampicin conjugates may open new avenues for further modifying the structure of this important antibiotic. Collectively, the results presented here contribute to the ongoing search for innovative antibacterial strategies in the fight against multidrug-resistant pathogens.

## Materials and Methods

### Synthesis

All reagents employed were of American Chemical Society (ACS) grade or higher and were used without further purification unless otherwise stated. Detailed synthesis of the compounds is provided in the Supporting Information.

### Bacterial Strains

The ATCC and NCTC reference strains used in this study, as well as JW 3594 Δ*rfaD*, JW 5503 Δ*tolC*, E. coli JW 0588 ΔentA, JW 0585 Δ*entC*, JW 0586 Δ*fepA*, JW 0584 Δ*fepB*, JW 0582 Δ*fepD*, JW 0146 Δ*fhuA,* JW 049 Δ*fhuB*, JW 0147 Δ*fhuC*, JW 0148 Δ*fhuD*, JW 1088 Δ*fhuE*, JW 4331 Δ*fhuF*, *KML-11668*, *RUH-134,* were commercially obtained or provided by Leiden University Medical Center (Leiden, Netherlands). MVAST0072, NR-48977, and NR-48978 were commercially obtained from BEI Resources. BW 25113, RC0089, and PAO1 were provided by the University Medical Center Utrecht (Utrecht, Netherlands). BW 25113 Δ*bamB* Δ*tolC* double deletion strain was provided by McMaster University (Hamilton, Canada). pRIVM_C029515_2, 2018-022, RIVM_C019741, and 2018-006 were supplied by the National Institute for Public Health and the Environment (Bilthoven, Netherlands). 1124 and 1427 were provided by VU University Medical Center Amsterdam (Amsterdam, Netherlands). EQAS 2016 (mcr-1) was provided by Wageningen University (Wageningen, Netherlands). NRZ-00687, NRZ-08418, NRZ-03961 were provided by The National Reference Centre (Bochum, Germany).

### Antibacterial Assay Against Gram-Negative and Gram-Positive Bacteria

From glycerol stocks, bacterial strains were cultured on blood agar plates and incubated overnight at 37 °C. Following incubation, 3 mL of tryptic soy broth (TSB) was inoculated with an individual colony. The cultures were grown to exponential phase (OD_600nm_ = 0.5) at 37 °C. The bacterial suspensions were then diluted 100-fold in cation-adjusted Mueller Hinton Broth (CAMHB) or iron-depleted cation-adjusted Mueller Hinton Broth (ID-CAMHB) to reach a bacterial cell density of 10^6^ CFU mL^–1^. In polypropylene 96-well microtiter plates, test compounds in assay media (e.g., CAMHB or ID-CAMHB) were added in triplicate and 2-fold serially diluted to achieve a final volume of 50 μL per well. An equal volume of bacterial suspension (50 μL, 10^6^ CFU mL^–1^) was added to the wells. The plates were sealed with breathable membranes and incubated at 37 °C for 18–22 h with constant shaking (600 rpm). The minimal inhibitory concentrations (MIC) were determined by visual inspection as the median of a minimum of triplicates.

ID-CAMHB was prepared as follows: 1 L of autoclaved Mueller Hinton broth was incubated with 100 g of cation binding resin Chelex 100 to remove cations, including iron, from the medium and filtered, and the pH was adjusted to 7.3 with hydrochloric acid. The medium was filtered again and supplemented with 20 to 25 mg/L of Ca^2+^ and 10 to 12.5 mg/L of Mg^2+^, according to CLSI recommendations.

#### Hemolysis Assay

The hemolytic activity of compounds was assessed in triplicate. Red blood cells from defibrillated sheep blood obtained from Thermo Fisher were centrifuged (400 g for 15 min at 4 °C) and washed with Phosphate-Buffered Saline (PBS) containing 0.002% Tween20 (buffer) five times. Then, the red blood cells were normalized to obtain a positive control read-out of 2.5 at 415 nm to stay within the linear range with the maximum sensitivity. A serial dilution of the compounds (64 to 2 μg/mL, 75 μL) was prepared in a 96-well plate. The outer border of the plate was filled with 75 μL buffer, the plate also contained a positive control (0.1% Triton-X final concentration, 75 μL) and a negative control (buffer, 75 μL) in triplicate. The normalized blood cells (75 μL) were added, and the plates were incubated at 37 °C for 1 h while shaking at 500 rpm. A flat-bottom plate of polystyrene with 100 μL buffer in each well was prepared. After incubation, the plate was centrifuged (800 g for 5 min at room temperature), and 25 μL of the supernatant was transferred to their respective wells in the flat-bottom plate. The values obtained from a read-out at 415 nm were corrected for background (negative control) and transformed to a percentage relative to the positive control (0.1% Triton-X).

## Supplementary Material



## Notes

General information, synthesis methods, analytical data, supplementary MIC results, hemolysis results, and HPLC traces for final conjugates (PDF)
