# Supplementary material for: Design, Synthesis, and Antibacterial Evaluation of Rifampicin–Siderophore Conjugates
Source: ACS Infect Dis. 2025 Jul 5;11(8):2301–9. doi: 10.1021/acsinfecdis.5c00311 (PMC12340970; doi:10.1021/acsinfecdis.5c00311)
Supplement: Supplementary file 1 [file id5c00311_si_001.pdf]

Supporting Information

Design, Synthesis, and Antibacterial Evaluation of Rifampicin-Siderophore Conjugates

Vladyslav Lysenko,[§] Mei-Ling Gao,[§] Fabienne A.C. Sterk, Paolo Innocenti, Cornelis J. Slingerland, Nathaniel I. Martin*

Biological Chemistry Group, Institute of Biology Leiden, Leiden University, Sylviusweg 72, 2333 BE Leiden, The Netherlands.

[§]Equal contribution

*All correspondence should be directed to n.i.martin@biology.leidenuniv.nl

Table of contents

	Page
**Table of contents**	S2
**General information**	S3
**Synthesis methods and analytical data**	S5
**Synthesis of rifampicin alkyne 2**	S5
**Synthesis of azide-containing building blocks**	S7
**Synthesis of triazole-linked conjugates 1, 8-13**	S10
**Synthesis of benzyl-protected catechols**	S12
**Synthesis of piperazine-containing building blocks**	S13
**Synthesis of amide S13**	S19
**Synthesis of alcohols 21-23, ester-linked conjugates 33-38, and amide 39**	S22
**Synthesis of enterobactin**	S26
**Supplementary MIC results**	S29
**Hemolysis**	S30
**NMR spectra**	S31
**HPLC traces**	S51
**References**	S60

General information

Reagents

All reagents employed were of American Chemical Society (ACS) grade or higher and were used without further purification unless otherwise stated.

HRMS

High-resolution mass spectra (HRMS) analyses were performed on a Shimadzu Nexera X2 UHPLC system with a Waters Acquity HSS C18 column (2.1 × 100 mm, 1.8 µm) at 30 °C and equipped with a diode array detector. The following solvent system, at a flow rate of 0.5 mL/min, was used: solvent A, 0.1 % formic acid in water; solvent B, 0.1 % formic acid in acetonitrile. Gradient elution was as follows: 95:5 (A/B) for 1 min, 95:5 to 15:85 (A/B) over 10 min, 15:85 to 0:100 (A/B) over 1 min, 0:100 (A/B) for 4 min, then reversion back to 95:5 (A/B) for 3 min. This system was connected to a Shimadzu 9030 QTOF mass spectrometer (ESI ionization) calibrated internally with Agilent's API-TOF reference mass solution kit (5.0 mM purine, 100.0 mM ammonium trifluoroacetate and 2.5 mM hexakis(1*H*,1*H*,3*H*-tetrafluoropropoxy)phosphazine) diluted to achieve a mass count of 10000.

Analytical HPLC

HPLC analyses were performed on a Shimadzu Prominence-i LC-2030 system with a Dr. Maisch ReproSil Gold 120 C18 column (4.6 × 250 mm, 5 µm) at 30 °C and equipped with a UV detector monitoring at 214 and 254 nm. The following solvent system, at a flow rate of 1 mL/min, was used: solvent A, 0.1 % TFA in water/acetonitrile 95/5; solvent B, 0.1 % TFA in water/acetonitrile 5/95. Gradient elution was as follows: 100:0 (A/B) for 3 min, 100:0 to 0:100 (A/B) over 47 min, 0:100 (A/B) for 4 min, then reversion back to 100:0 (A/B) over 1 min, 100:0 (A/B) for 5 min.

Preparative HPLC

Method A

The compounds were purified using a BESTA-Technik system with a Dr. Maisch Reprosil Gold 120 C18 column (25 × 250 mm, 10 µm) and equipped with an ECOM Flash UV detector monitoring at 214 nm. At a flow rate of 12 mL/min, the following solvent system was used: solvent A, 0.1 % TFA in water/acetonitrile 95:5; solvent B, 0.1 % TFA in water/acetonitrile 5:95. Gradient elution was as follows: 100:0 (A/B) for 3 min, 100:0 to 40:60 (A/B) over 47 min, 40:60 to 0:100 (A/B) over 1 min, 0:100 (A/B) for 4 min, then reversion back to 100:0 (A/B) over 1 min, 100:0 (A/B) for 4 min. The fractions were analyzed, combined, and lyophilized to obtain purified compounds.

Method B

The compounds were purified using a BESTA-Technik system with a Dr. Maisch Reprosil Gold 120 C18 column (25 × 250 mm, 10 µm) and equipped with an ECOM Flash UV detector monitoring at 214 nm. At a flow rate of 12 mL/min, the following solvent system was used: solvent A, 0.1 % TFA in water/acetonitrile 95:5; solvent B, 0.1 % TFA in water/acetonitrile 5:95. Gradient elution was as follows: 80:20 (A/B) for 3 min, 80:20 to 0:100 (A/B) over 47 min, 0:100 (A/B) for 5 min, then reversion back to 80:20 (A/B) over 1 min, 80:20 (A/B) for 4 min. The fractions were analyzed, combined, and lyophilized to obtain purified compounds.

NMR

^{1}H and ^{13}C NMR spectra were recorded on Bruker AV 400 MHz (at 400 (^{1}H), and 101 (13C) MHz), AV 600 MHz (at 600 (1H) and 151 (^{13}C) MHz). The temperature of the NMR experiments was 298 K unless stated otherwise. Chemical shifts are reported in ppm (δ) and were calibrated using residual deuterated solvent as an internal reference. (δ ^{1}H NMR: $CDCl_3$ 7.26; DMSO 2.50; δ ^{13}C NMR: $CDCl_3$ 77.16; DMSO 39.52). The NMR data is processed as follows: chemical shift, multiplicity (br s = broad singlet, s = singlet, d = doublet, dd = double doublet, t = triplet, dt = double triplet, q = quartet, tt = triple triplet,

m = multiplet), integration, coupling constants (*J*, reported in Hz) and a number of nuclei. NMR spectra were analyzed and processed using MestreNova version 14.2.0.

Bacterial strains

The ATCC reference strains used in this study, as well as *E.coli* JW 3594 ΔrfaD, *E.coli* JW 0588 ΔentA, *E.coli* JW 0585 ΔentC, *E.coli* JW 0586 ΔfepA, *A. baumannii KML-11668* were commercially obtained or provided by Leiden University Medical Center (Leiden, Netherlands). The standard lab strain *E. coli* BW 25113 was provided by University Medical Center Utrecht (Utrecht, Netherlands). *E. coli* BW25113 ΔbamBΔtolC double deletion strain was provided by McMaster University (Hamilton, Canada). *E. coli* pRIVM_C029515_2 and *K. pneumoniae* RIVM_C019741, *A. baumannii* 2018-006 (NDM/OXA-023/OXA-051) were supplied by the National Institute for Public Health and the Environment (Bilthoven, Netherlands). *K. pneumoniae* 1124 was provided by VU University Medical Center Amsterdam (Amsterdam, Netherlands). *P. aeruginosa* NRZ03961 was provided by The National Reference Centre (Bochum, Germany).

Antibacterial assay against Gram-negative and Gram-positive bacteria

From glycerol stocks, bacterial strains were cultured on blood agar plates and incubated overnight at 37 °C. Following incubation, 3 mL of tryptic soy broth (TSB) was inoculated with an individual colony. The cultures were grown to exponential phase (OD_{600nm} = 0.5) at 37 °C. The bacterial suspensions were then diluted 100-fold in cation-adjusted Mueller Hinton Broth (CAMHB) or iron-depleted cation-adjusted Mueller Hinton Broth (ID-CAMHB) to reach a bacterial cell density of 10^6 CFU mL^{-1}. In polypropylene 96-well microtiter plates, test compounds in assay media (e.g., CAMHB or ID-CAMHB) were added in triplicate and two-fold serially diluted to achieve a final volume of 50 µL per well. An equal volume of bacterial suspension (50 µL, 10^6 CFU mL^{-1}) was added to the wells. The plates were sealed with breathable membranes and incubated at 37 °C for 18-22 h with constant shaking (600 rpm). The minimal inhibitory concentrations (MIC) were determined by visual inspection as the median of a minimum of triplicates.

ID-CAMHB was prepared as follows: one liter of autoclaved Mueller Hinton broth was incubated with 100 g of cation binding resin Chelex 100 to remove cations, including iron, from the medium and filtered, and the pH was adjusted to 7.3 with hydrochloric acid. The medium was filtered again and supplemented with 20 to 25 mg/L of Ca^{2+} and 10 to 12.5 mg/L of Mg^{2+}, according to CLSI recommendations.

Hemolysis Assay

The hemolytic activity of compounds was assessed in triplicate. Red blood cells from defibrillated sheep blood obtained from Thermo Fisher were centrifuged (400 g for 15 min at 4 °C) and washed with Phosphate-Buffered Saline (PBS) containing 0.002% Tween20 (buffer) five times. Then, the red blood cells were normalized to obtain a positive control read-out of 2.5 at 415 nm to stay within the linear range with the maximum sensitivity. A serial dilution of the compounds (64 to 2 µg/mL, 75 µL) was prepared in a 96-well plate. The outer border of the plate was filled with 75 µL buffer, the plate also contained a positive control (0.1% Triton-X final concentration, 75 µL) and a negative control (buffer, 75 µL) in triplicate. The normalized blood cells (75 µL) were added, and the plates were incubated at 37 °C for 1 h while shaking at 500 rpm. A flat-bottom plate of polystyrene with 100 µL buffer in each well was prepared. After incubation, the plate was centrifuged (800 g for 5 min at room temperature), and 25 µL of the supernatant was transferred to their respective wells in the flat-bottom plate. The values obtained from a read-out at 415 nm were corrected for background (negative control) and transformed to a percentage relative to the positive control (0.1% Triton-X).

Synthesis methods and analytical data

Synthesis of rifampicin alkyne 2

Rifampicin alkyne **2** was synthesized following previously published methods with minor changes**.** Spectral data obtained for all intermediates and rifampicin alkyne itself are in agreement with those reported in the literature.[1,2]

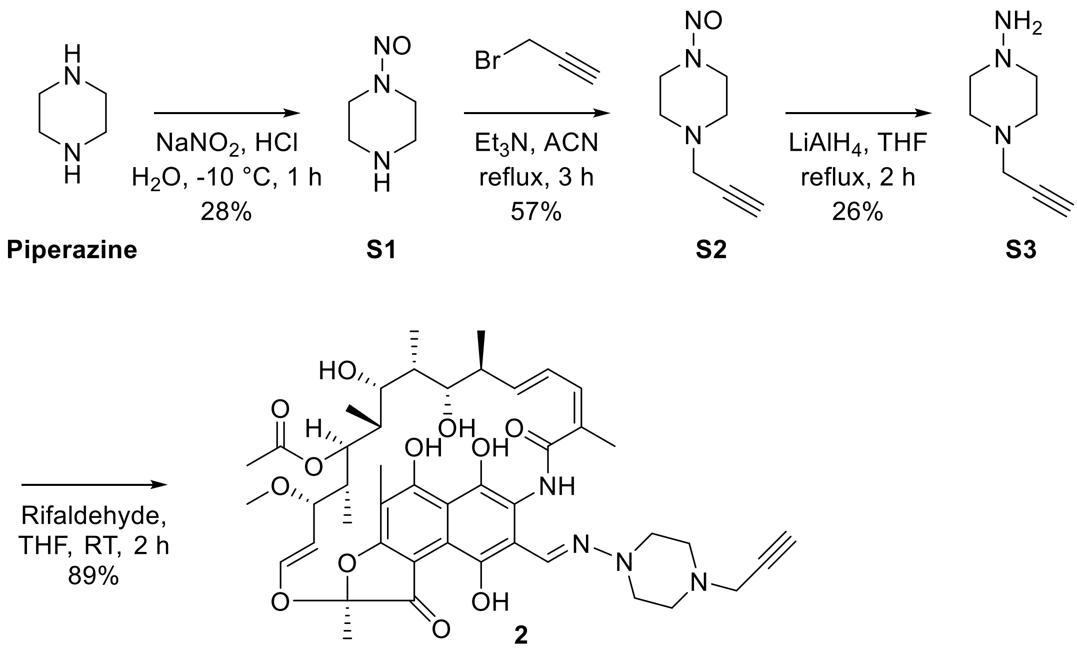

Scheme S1**. Synthesis of Rifampicin alkyne **2

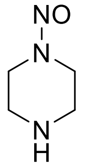

1-nitrosopiperazine (S1). Piperazine (5.16 g, 60 mmol) was dissolved in 6M HCl (36 mL) and cooled to -10 °C. A solution of $NaNO_2$ (4.14 g, 60 mmol) in H_2O (72 mL) was added slowly by an addition funnel over 2 hours. The reaction mixture was adjusted to pH 10 with 3M NaOH and extracted with DCM (3 × 100 mL). The combined organic extracts were dried over anhydrous Na_2SO4, concentrated in vacuo, and purified by flash column chromatography (SiO_2, 5% MeOH in DCM) to yield the compound **S1** as a yellow oil (1.98 g, 28%).

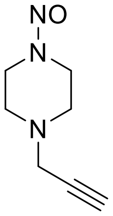

1-nitroso-4-propargylpiperazine (S2). 1-Nitrosopiperazine (**S1**) (1.2 g, 10.42 mmol) and propargyl bromide (1.16 mL of 80% solution in toluene, 10.42 mmol) were dissolved in dry ACN (30 mL) and Et_3N (2.91 mL, 20.85 mmol). The reaction mixture was stirred at 75 °C for 3 hours and concentrated in vacuo. The crude was then dissolved in 10% NaOH (100 mL) and extracted with DCM (3 × 60 mL). The organic phase was dried over anhydrous Na_2SO_4, concentrated in vacuo, and purified by column chromatography (SiO_2, EtOAc/PE= 2:1) to yield the compound **S2** as an orange oil (900 mg, 57%).

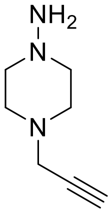

1-amino-4-propargylpiperazine (S3). 1-Nitroso-4-propargylpiperazine (**S2**) was dissolved in dry THF and cooled to 0 °C. $LiAlH_4$ (1 M in THF, 6.00 mL, 2.94 mmol) was added slowly and was stirred at 35 °C for 2 hours. The reaction mixture was then cooled, quenched with 2 M HCl (5 mL), and filtered through celite. The celite was subsequently washed with 2 M HCl (3 × 10 mL), and the filtrate was adjusted to pH 10 with 10% aq. NaOH. The aqueous phase was extracted with 3:1 $CHCl_3$/i-PrOH (4 × 350 mL), and the organic phase dried over anhydrous Na_2SO_4 and concentrated in vacuo. The crude product was purified by column chromatography (SiO_2, 10% MeOH in DCM) to yield the alkyne **S3** as an off-white solid (107 mg, 26%).

^{1}H NMR (400 MHz, $CDCl_3$) δ 3.30 (d, J = 2.5 Hz, 2H), 3.02 (s, 2H), 2.65 (s, 6H), 2.25 (t, J = 2.4 Hz, 1H).

^{13}C{^{1}H} NMR (101 MHz, $CDCl_3$) δ 78.7, 73.4, 59.3, 51.8, 46.6.

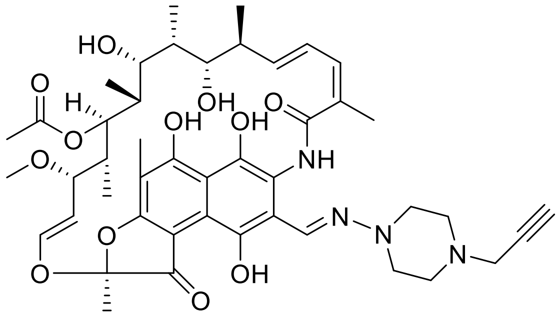

Rifampicin alkyne (2). Rifaldehyde (130 mg, 0.18 mmol) and 1-amino-4-propargylpiperazine (**S3**) (30 mg, 0.21 mmol) were dissolved in dry THF (3 mL), and the mixture was stirred vigorously for 2 hours. The mixture was then diluted with DCM (8 mL) and washed with 8 mL of a solution of ascorbic acid (2 g) in 3:1 H_2O/brine (40 mL). The aqueous phase was then extracted with DCM (2 × 8 mL), and the combined DCM extracts were dried over anhydrous Na_2SO_4 and concentrated in vacuo to yield rifampicin alkyne **2** as a red solid (136 mg, 89%).

^{1}H NMR (400 MHz, $CDCl_3$) δ 12.04 (s, 1H), 8.30 (s, 1H), 6.64 – 6.52 (m, 1H), 6.39 (d, J = 11.1 Hz, 1H), 6.21 (dd, J = 12.7, 1.1 Hz, 1H), 5.94 (dd, J = 15.5, 5.0 Hz, 1H), 5.10 (dd, J = 12.7, 6.8 Hz, 1H), 4.94 (d, J = 10.6 Hz, 1H), 3.78 (s, 1H), 3.61 (d, J = 4.9 Hz, 1H), 3.51 – 3.44 (m, 2H), 3.42 (s, 1H), 3.23 (s, 1H), 3.14 (s, 1H), 3.04 (s, 3H), 3.03 – 2.99 (m, 1H), 2.77 (s, 2H), 2.38 (d, J = 7.0 Hz, 1H), 2.32 (s, 1H), 2.23 (s, 3H), 2.07 (d, J = 7.5 Hz, 6H), 1.80 (s, 3H), 1.74 – 1.68 (m, 1H), 1.59 – 1.50 (m, 1H), 1.41 – 1.31 (m, 1H), 1.02 (d, J = 7.0 Hz, 3H), 0.89 (d, J = 7.0 Hz, 3H), 0.60 (d, J = 6.9 Hz, 3H), -0.31 (d, J = 6.9 Hz, 3H).

HRMS (ESI) m/z: $[M+H]^+$ calcd for $C_{45}H_{58}N_4O_{12}+H^+$: 847.4124; found: 847.4134.

<u>**Synthesis of azide-containing building blocks**</u>

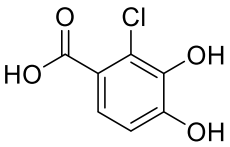

2-chloro-3,4-dihydroxybenzoic acid (6). Into a solution of 2-chloro-3,4-dimethoxybenzoic acid (**5**) (2 g, 9.23 mmol) in DCM (30 mL) was added a 1M solution of BBr_3 in DCM (36.93 mL, 36.93 mmol) over 10 min at 0°C under Ar. After that, the reaction mixture was allowed to warm up to RT and was left stirring for an additional 3 h before it was poured into cold 2M HCl (100 mL) and then extracted with EtOAc (2 × 100 mL). The organic layers were combined and washed with H_2O (100 mL), brine (50 mL), dried over Na_2SO_4, and concentrated in vacuo to give compound **6** (1.63 g, 94% yield).

^{1}H NMR (400 MHz, DMSO-*d6*) δ 12.65 (br s, 1H), 10.41 (s, 1H), 9.31 (br s, 1H), 7.23 (d, *J* = 8.5 Hz, 1H), 6.76 (d, *J* = 8.5 Hz, 1H).

^{13}C{^{1}H} NMR (101 MHz, DMSO-*d6*) δ 166.6, 149.6, 142.6, 122.5, 121.6, 120.3, 112.8.

HRMS (ESI) m/z: $[M-H]^-$ calcd for $C_7H_5ClO_4$-H^+: 186.9803; found: 186.9805.

CAUTION! Azides with a high Nitrogen/Carbon ratio can be explosive.

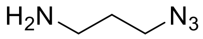

3-azidopropan-1-amine (4). To a solution of 3-chloropropan-1-amine hydrochloride salt (**3**) (3 g, 23.1 mmol) in water (15 ml) was added NaN_3 (4.5 g, 69.3 mmol), and the reaction was heated at 80 °C for 15 h. The solution was then basified with KOH (3.24 g, 57.8 mmol) and extracted with diethyl ether (3 × 30 mL). The combined organic layers were dried over anhydrous Na_2SO_4, filtered, and the solvent was removed in vacuo at 25°C to give compound **4** (1.8 g, 78% yield) as a colorless oil.

^{1}H NMR (400 MHz, $CDCl_3$) δ 3.38 (t, *J* = 6.7 Hz, 1H), 2.82 (t, *J* = 6.8 Hz, 1H), 1.74 (p, *J* = 6.8 Hz, 1H), 1.26 (br. s, 2H).

Spectral data are in agreement with those reported in the literature.[3–5]

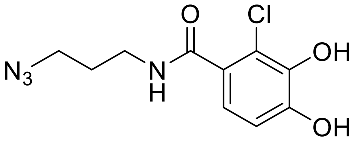

N-(3-azidopropyl)-2-chloro-3,4-dihydroxybenzamide (7). Into a solution of 3-azidopropan-1-amine (**4**) (265 mg, 2.65 mmol) in DCM (10 mL) and DMF (5 mL) were added HOBt (447.1 mg, 2.92 mmol), DIPEA (810 µL, 4.64 mmol), 2-chloro-3,4-dihydroxybenzoic acid (**6**) (250 mg, 1.33 mmol) and EDC*HCl (559.8 mg, 2.92 mmol), respectively, at RT. After stirring for 4 h, the reaction mixture was diluted with EtOAc (50 mL) and washed with sat. NH_4Cl (10 mL) and brine. The organic phase was dried over Na_2SO_4 and concentrated in vacuo to obtain the crude, which was purified using the HPLC method B to afford compound **7** (248 mg, 69% yield) as a colorless oil.

^{1}H NMR (400 MHz, DMSO-*d_6*) δ 9.89 (br s, 1H), 9.37 (br s, 1H), 8.21 (t, J = 5.7 Hz, 1H), 6.76 – 6.70 (m, 2H), 3.41 (t, *J* = 6.8 Hz, 2H), 3.23 (q, J = 6.4 Hz, 2H), 1.73 (p, J = 6.8 Hz, 2H).

^{13}C{^{1}H} NMR (101 MHz, DMSO-*d_6*) δ 166.9, 147.3, 142.1, 128.8, 118.8, 117.8, 113.1, 48.6, 36.4, 28.4.

HRMS (ESI) m/z: $[M+H]^+$ calcd for $C_{10}H_{11}ClN_4O_3$+H^+: 271.0593; found: 271.0595.

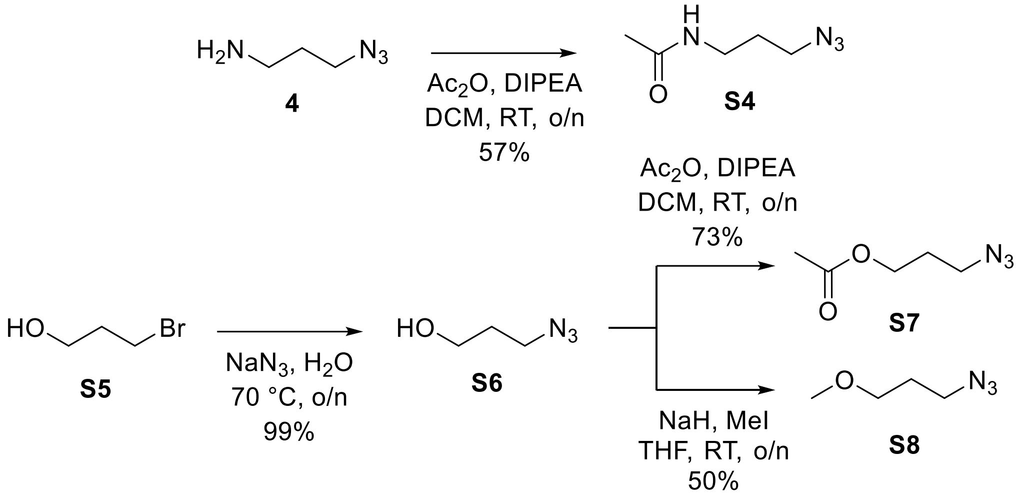

***Scheme S2**. Synthesis of the azide derivatives for the click-chemistry reaction with compound **2**.*

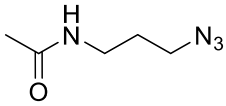

N-(3-azidopropyl)acetamide (S4). 3-Azidopropan-1-amine (**4**) (100 mg, 1 mmol) was dissolved in DCM (3 mL), and then DIPEA (174 µL, 1 mmol) was added, followed by Ac_2O (94.5 µL, 1 mmol), and the reaction was left stirring overnight under an Ar atmosphere. The next day, the solution was diluted with 1M HCl (5 mL) and extracted with DCM (5 mL). The organic layer was washed with 1M $NaHCO_3$ (5 mL), water (5 mL), and brine (5 mL), then dried over Na_2SO_4 and concentrated in vacuo at 25°C to obtain compound **S4** (81 mg, 57% yield) that was used as a substrate in the click-chemistry reaction without further purification.

^{1}H NMR (400 MHz, $CDCl_3$) δ 5.74 (br s, 1H), 3.40 – 3.28 (m, 4H), 1.98 (s, 3H), 1.85 – 1.70 (m, 2H).

Spectral data are in agreement with those reported in the literature.[6,7]

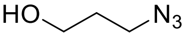

3-azidopropan-1-ol (S6). To a solution of 3-bromopropan-1-ol (**S5**) (3 g, 21.6 mmol) in water (15 mL) was added NaN_3 (4.21 g, 64.7 mmol), and the reaction was heated at 80 °C for 15 h. The reaction mixture was then extracted with diethyl ether (3 × 30 mL). The combined organic layers were dried over anhydrous Na_2SO_4, filtered, and the solvent was removed in vacuo at 25°C to give compound **S6** (2.2 g, 99% yield) as a yellow oil.

^{1}H NMR (400 MHz, $CDCl_3$) δ 3.76 (t, *J* = 6.0 Hz, 2H), 3.46 (t, *J* = 6.6 Hz, 2H), 1.84 (p, *J* = 6.4 Hz, 2H), 1.61 (s, 1H).

Spectral data are in agreement with those reported in the literature.[8–10]

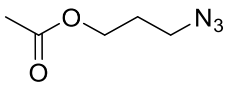

3-azidopropyl acetate (S7). 3-Azidopropan-1-ol (**S6**) (100 mg, 0.99 mmol) was dissolved in DCM (3 mL), and then DIPEA (172.3 µL, 0.99 mmol) was added, followed by Ac_2O (93.5 µL, 0.99 mmol), and the reaction was left stirring overnight under an Ar atmosphere. The next day, the solution was diluted with 1M HCl (5 mL) and extracted with DCM (5 mL). The organic layer was washed with 1M $NaHCO_3$ (5 mL), water (5 mL), and brine (5 mL), then dried over Na_2SO_4 and concentrated in vacuo at 25°C to obtain compound **S7** (104 mg, 73% yield) that was used as a substrate in the click-chemistry reaction without further purification.

^{1}**H NMR** (400 MHz, $CDCl_3$) δ 4.15 (t, *J* = 6.2 Hz, 2H), 3.39 (t, *J* = 6.7 Hz, 2H), 2.06 (s, 3H), 1.90 (p, *J* = 6.5 Hz, 2H).

Spectral data are in agreement with those reported in the literature.[11]

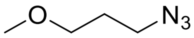

1-azido-3-methoxypropane (S8). 3-Azidopropan-1-ol (**S6**) (200 mg, 1.98 mmol) in THF (1 mL) was added dropwise to NaH (60% dispersion in mineral oil, 119 mg, 2.97 mmol) in DMF (3 mL) at 0 °C, and the reaction mixture was stirred for 30 min. After that, MeI (148 µL, 2.37 mmol) was added dropwise. The reaction mixture was allowed to warm to room temperature and then left stirring for an additional 3 h. The reaction was quenched by pouring into water (10 mL) and extracted with diethyl ether (20 mL). The organic layer was washed with water (3 × 10 mL) and brine (10 mL), dried over anhydrous Na_2SO_4, filtered, and concentrated in vacuo at 25°C to obtain the compound **S8** (115 mg, 50% yield), which was used as a substrate in the click-chemistry reaction without further purification.

^{1}**H NMR** (400 MHz, $CDCl_3$) δ 3.45 (t, *J* = 6.0 Hz, 2H), 3.38 (t, *J* = 6.7 Hz, 2H), 3.33 (s, 3H), 1.83 (p, *J* = 6.6 Hz, 2H).

Spectral data are in agreement with those reported in the literature.[12,13]

Synthesis of triazole-linked conjugates 1, 8-13

General Procedure A (conditions for the conjugation using click-chemistry approach). The azide (0.04 mmol) and rifampicin alkyne (**2**) (0.02 mmol) were dissolved in 1:1 H_2O/tBuOH (650 µL). Freshly prepared aqueous solutions of ascorbic acid (500 mM, 85 µL, 0.04 mmol) and $CuSO_4 \cdot 5H_2O$ (100 mM, 100 µL, 0.01 mmol) were added, and the mixture was stirred at 50 °C for 2 h. After that, the reaction mixture was spiked with ascorbic acid (500 mM, 85 µL, 0.04 mmol), and then it was directly purified using the preparative HPLC method B. The fractions containing the pure product, as judged by HPLC, were combined and lyophilized to yield the corresponding triazole conjugates.

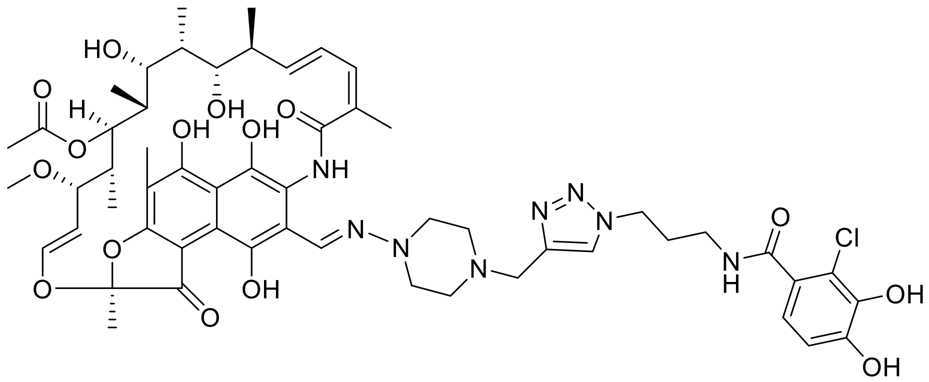

Conjugate 1. The compound was prepared according to General Procedure A, starting from rifampicin alkyne **2** (16.9 mg, 0.02 mmol) and azide **7** (110.8 mg, 0.04 mmol). Yield: 9 mg, 40%, orange powder.

HRMS (ESI) m/z: $[M+H]^+$ calcd for $C_{55}H_{69}ClN_8O_{15}+H^+$: 1117.4644; found: 1117.4633.

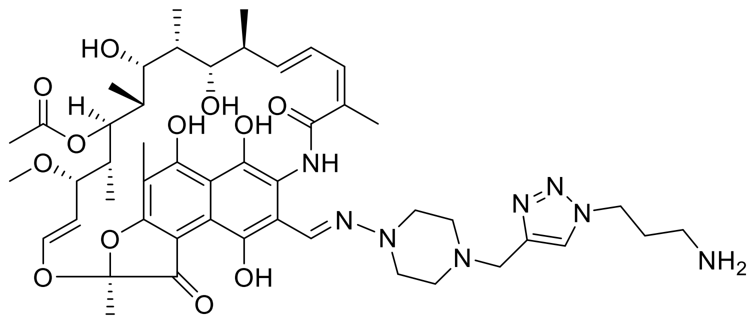

Conjugate 8. The compound was prepared according to General Procedure A, starting from rifampicin alkyne **2** (5 mg, 0.0059 mmol) and azide **4** (1.2 mg, 0.0118 mmol). Yield: 2.3 mg, 41%, orange powder.

HRMS (ESI) m/z: $[M+H]^+$ calcd for $C_{48}H_{66}N_8O_{12}+H^+$: 947.4873; found: 947.4864.

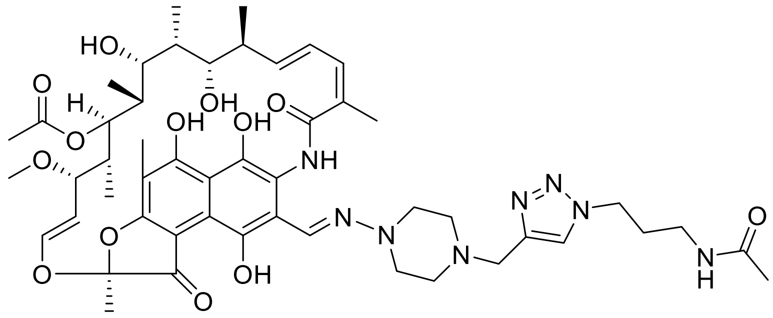

Conjugate 9. The compound was prepared according to General Procedure A, starting from rifampicin alkyne **2** (5 mg, 0.0059 mmol) and azide **S4** (1.7 mg, 0.0118 mmol). Yield: 2.5 mg, 43%, orange powder.

HRMS (ESI) m/z: $[M+H]^+$ calcd for $C_{50}H_{68}N_8O_{13}+H^+$: 989.4979; found: 989.4972.

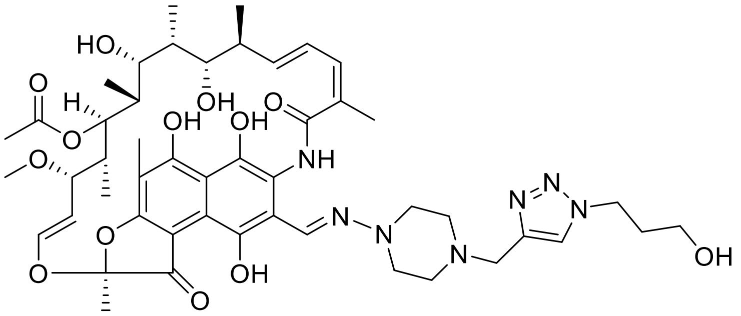

Conjugate 10. The compound was prepared according to General Procedure A, starting from rifampicin alkyne **2** (5 mg, 0.0059 mmol) and azide **S6** (1.2 mg, 0.0118 mmol). Yield: 2.6 mg, 47%, orange powder.

HRMS (ESI) m/z: $[M+H]^+$ calcd for $C_{48}H_{65}N_7O_{13}+H^+$: 948.4713; found: 948.4708.

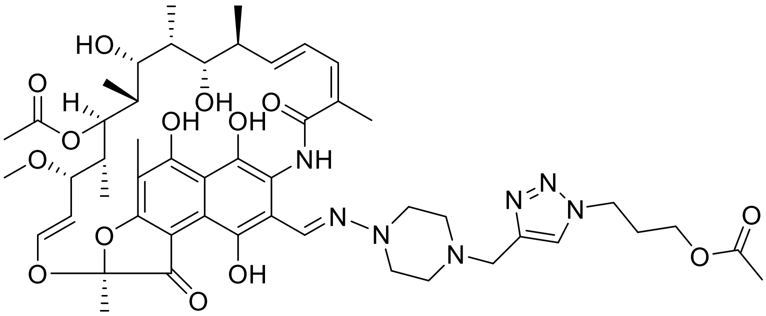

Conjugate 11. The compound was prepared according to General Procedure A, starting from rifampicin alkyne **2** (5 mg, 0.0059 mmol) and azide **S7** (1.7 mg, 0.0118 mmol). Yield: 2.3 mg, 39%, orange powder.

HRMS (ESI) m/z: $[M+H]^+$ calcd for $C_{50}H_{67}N_7O_{14}+H^+$: 990.4819; found: 990.4813.

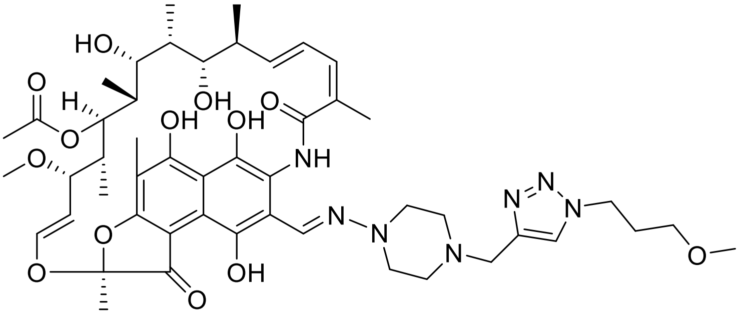

Conjugate 12. The compound was prepared according to General Procedure A, starting from rifampicin alkyne **2** (5 mg, 0.0059 mmol) and azide **S8** (1.4 mg, 0.0118 mmol). Yield: 2.4 mg, 42%, orange powder.

HRMS (ESI) m/z: $[M+H]^+$ calcd for $C_{49}H_{67}N_7O_{13}+H^+$: 962.4870; found: 962.4869.

Synthesis of benzyl-protected catechols

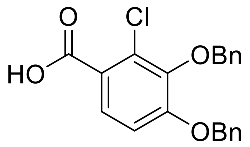

3,4-bis(benzyloxy)-2-chlorobenzoic acid (25). To a solution of 2-chloro-3,4-dihydroxybenzoic acid (**6**) (1.57 g, 8.33 mmol) and benzyl bromide (4.98 g, 29.1 mmol) in DMF (20 mL) was added pulverized K_2CO_3 (5.75 g, 41.6 mmol). After being stirred for 16 h, the reaction was quenched with 100 mL of distilled water and extracted with EtOAc (2 × 70 mL). The combined organic layers were washed with sat. NH_4Cl (70 mL), water (2 × 70 mL), and brine (50 mL), dried over anhydrous Na_2SO_4 and concentrated in vacuo. The obtained crude was then dissolved in the mixture of MeOH (30 mL), THF (10 mL), and 6 M aq. NaOH (10 mL) and was left stirring for 15 h at RT. After that, MeOH was evaporated, and the mixture was diluted with water (50 mL), washed with Et_2O (2 × 60 mL), and acidified with aq. HCl to form a white precipitate, which was filtered, washed with water (2 x 10 mL), petroleum ether (2 x 15 mL), and then dried to obtain compound **25** (2.85 g, 93% yield) as a white solid.

^{1}H NMR (400 MHz, DMSO-*d6*) δ 13.09 (s, 1H), 7.65 (d, *J* = 8.8 Hz, 1H), 7.54 – 7.47 (m, 2H), 7.46 – 7.29 (m, 8H), 7.24 (d, *J* = 8.9 Hz, 1H), 5.26 (s, 2H), 4.97 (s, 2H).

^{13}C{^{1}H} NMR (101 MHz, DMSO-*d6*) δ 166.1, 155.1, 144.3, 136.8, 136.2, 128.6, 128.5, 128.3, 128.2, 128.2, 128.0, 127.7, 127.2, 123.7, 112.0, 74.2, 70.4.

HRMS (ESI) m/z: $[M+H]^+$ calcd for $C_{21}H_{17}ClO_4+H^+$: 369.0888; found: 369.0892.

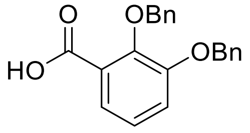

2,3-bis(benzyloxy)benzoic acid (26). To a solution of 2,3-dihydroxybenzoic acid (**24**) (5 g, 32.4 mmol) and benzyl bromide (19.4 g, 113.4 mmol) in DMF (40 mL), pulverized K_2CO_3 (22.4 g, 162 mmol) was added. After being stirred for 16 h, the reaction was quenched with 200 mL of water and extracted with EtOAc (2 × 150 mL). The combined organic layers were washed with sat. NH_4Cl (150 mL), water (2 × 150 mL), and brine (100 mL), dried over anhydrous Na_2SO_4 and concentrated in vacuo. The obtained crude was then dissolved in the mixture of MeOH (150 mL), THF (20 mL), and 6 M aq. NaOH (30 mL) and was left stirring for 15 h at RT. After that, MeOH was evaporated, and the mixture was diluted with water (100 mL), washed with Et_2O (2 × 60 mL), and acidified with aq. HCl to form a white precipitate, which was filtered, washed with water, petroleum ether, and then dried to obtain compound **26** (10.2 g, 94% yield) as a white solid.

^{1}H NMR (400 MHz, DMSO-*d6*) δ 12.95 (br s, 1H), 7.53 – 7.46 (m, 2H), 7.44 – 7.25 (m, 9H), 7.25 – 7.11 (m, 2H), 5.19 (s, 2H), 5.00 (s, 2H).

^{13}C{^{1}H} NMR (101 MHz, DMSO-*d6*) δ 167.5, 152.3, 146.6, 137.5, 136.8, 128.5, 128.2, 128.1, 128.0, 127.8, 127.8, 124.2, 121.5, 117.0, 74.7, 70.2.

HRMS (ESI) m/z: $[M+H]^+$ calcd for $C_{21}H_{18}O_4+H^+$: 335.1278; found: 335.1282.

Synthesis of piperazine-containing building blocks

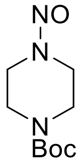

tert-butyl 4-nitrosopiperazine-1-carboxylate (14). tert-Butyl piperazine-1-carboxylate (**13**) (10 g, 53.69 mmol) and tert-butyl nitrite (13.84 g, 134.23 mmol) were mixed together in a round-bottom flask, and the mixture was left stirring at 50°C under an Ar atmosphere for 24 h. After that, the excess of tert-butyl nitrite was evaporated under reduced pressure to obtain compound **14** (11.56 g, quantitative yield) as yellow crystals.

^{1}H NMR (400 MHz, $CDCl_3$) δ 4.28 – 4.21 (m, 2H), 3.83 – 3.76 (m, 2H), 3.70 – 3.63 (m, 2H), 3.47 – 3.40 (m, 2H), 1.47 (s, 9H).

^{13}C{^{1}H} NMR (101 MHz, $CDCl_3$) δ 154.4, 81.0, 49.4, 39.8, 28.4.

HRMS (ESI) m/z: $[M+H]^+$ calcd for $C_9H_{17}N_3O_3+H^+$: 216.1343; found: 216.1346.

Spectral data are in agreement with those reported in the literature.[14]

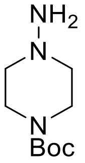

tert-butyl 4-aminopiperazine-1-carboxylate (15). To a stirred solution of tert-butyl 4-nitrosopiperazine-1-carboxylate (**14**) (16 g, 74.33 mmol) in THF/H_2O (1:1, 500 mL), NH_4Cl (64 g, 1.19 mol) was added, followed by portion-wise addition of Zn dust (39 g, 0.59 mol). After the addition was completed, the reaction mixture was left stirring at room temperature overnight. After that, the reaction mixture was filtered, and the filtrate was extracted with MTBE (2 × 300 mL). Organic layers were combined, washed with brine (100 mL), dried over anhydrous Na_2SO_4, and concentrated under reduced pressure to obtain compound **15** (7.18 g, 48% yield) as a white solid.

^{1}H NMR (400 MHz, $CDCl_3$) δ 3.46 (br s, 4H), 3.05 (br s, 2H), 2.55 (br s, 4H), 1.44 (s, 9H).

^{13}C{^{1}H} NMR (101 MHz, $CDCl_3$) δ 154.7, 80.0, 59.2, 46.0, 28.5.

HRMS (ESI) m/z: $[M+H]^+$ calcd for $C_9H_{19}N_3O_2+H^+$: 202.1550; found: 202.1553.

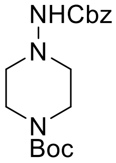

tert-butyl 4-(((benzyloxy)carbonyl)amino)piperazine-1-carboxylate (16). To a stirred solution of tert-butyl 4-aminopiperazine-1-carboxylate (**15**) (6.34 g, 31.5 mmol) in THF (120 mL) was added Et_3N (8.78 mL, 63 mmol), followed by dropwise addition of CbzCl (4.5 mL, 31.5 mmol). After the addition was completed, the reaction mixture was left stirring at room temperature overnight. After that, the reaction mixture was diluted with water (100 mL) and extracted with MTBE (200 mL). The organic layer was separated, washed with water (100 mL) and brine (50 mL), dried over anhydrous Na_2SO_4, and concentrated under reduced pressure. The residue was triturated with petroleum ether (50 mL) and filtered. The precipitate was washed with petroleum ether (2 × 30 mL) and dried to obtain compound **16** (9.17 g, 87% yield) as white crystals.

^{1}**H NMR** (400 MHz, $CDCl_3$) δ 7.41 – 7.27 (m, 5H), 5.74 (br s, 1H), 5.13 (s, 2H), 3.57 – 3.50 (m, 4H), 2.79 – 2.72 (m, 4H), 1.45 (s, 9H).

^{13}**C{^{1}H} NMR** (101 MHz, $CDCl_3$) δ 154.6, 136.2, 128.7, 128.4, 80.2, 67.2, 56.0, 55.7, 43.3, 28.5.

HRMS (ESI) m/z: $[M+H]^+$ calcd for $C_{17}H_{25}N_3O_4+H^+$: 336.1918; found: 336.1930.

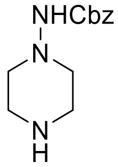

benzyl piperazin-1-ylcarbamate (17). TFA (15 mL) was added to a stirred solution of tert-butyl 4-(((benzyloxy)carbonyl)amino)piperazine-1-carboxylate (**16**) (9.1 g, 27.13 mmol) in DCM (15 mL), and the mixture was left stirring for 1 h. The volatile solvents were evaporated, and the residue was triturated with MTBE (30 mL) to obtain the precipitate, which was filtered, washed with MTBE (2 × 30 mL), and dried to give the compound **17** (3.32 g, 52% yield, calculated for 2 × TFA counterions) as a white powder.

^{1}**H NMR** (400 MHz, DMSO-d_6) δ 9.03 (br s, 2H), 8.90 (br s, 1H), 7.41 – 7.27 (m, 5H), 5.03 (s, 2H), 3.19 – 3.12 (m, 4H), 3.01 – 2.89 (m, 4H).

^{13}**C{^{1}H} NMR** (101 MHz, DMSO-d_6) δ 154.8, 136.9, 128.5, 128.0, 127.9, 65.5, 51.3, 42.8.

HRMS (ESI) m/z: $[M+H]^+$ calcd for $C_{12}H_{17}N_3O_2+H^+$: 236.1394; found: 236.1399.

General Procedure B (alkylation of piperazines). Bromoalcohol (4.32 mmol) was added to a stirred suspension of piperazine **17** (1 g, 2.16 mmol) and K_2CO_3 (1.5 g, 10.79 mmol) in ACN (15 mL), and the mixture was left stirring at 80°C overnight. The next day, the solvent was evaporated, and the residue was redissolved in water (30 mL), followed by extraction with EtOAc (2 × 30 mL). The combined organic layers were washed with brine (15 mL), dried over anhydrous Na_2SO_4, and concentrated under reduced pressure. The residue was triturated with petroleum ether (10 mL) and filtered. The precipitate was washed with petroleum ether (2 × 10 mL) and dried to obtain the product.

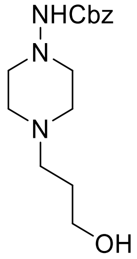

benzyl (4-(3-hydroxypropyl)piperazin-1-yl)carbamate (18). The compound was prepared according to General Procedure B, starting from piperazine **17** (1 g, 2.16 mmol) and 3-bromopropanol (0.6 g, 4.32 mmol). Yield: 0.42 g, 66%, white powder. For analytical purposes, the compound was purified via the preparative HPLC method A. NMR data are provided for the TFA salt.

^{1}**H NMR** (400 MHz, DMSO-d_6) δ 9.75 (br s, 1H), 8.89 (br s, 1H), 7.42 – 7.27 (m, 5H), 5.04 (s, 2H), 3.46 (t, J = 5.9 Hz, 4H), 3.16 – 2.90 (m, 8H), 1.82 – 1.71 (m, 2H).

^{13}**C{^{1}H} NMR** (101 MHz, DMSO-d_6) δ 154.7, 136.8, 128.4, 128.0, 127.9, 65.5, 58.0, 53.6, 51.2, 50.8, 26.7.

HRMS (ESI) m/z: $[M+H]^+$ calcd for $C_{15}H_{23}N_3O_3+H^+$: 294.1812; found: 294.1826.

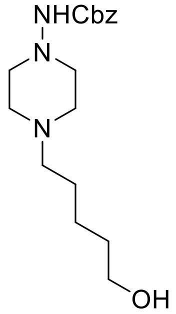

benzyl (4-(5-hydroxypentyl)piperazin-1-yl)carbamate (19). The compound was prepared according to General Procedure B, starting from piperazine **17** (1 g, 2.16 mmol) and 5-bromopropanol (0.72 g, 4.32 mmol). Yield: 0.48 g, 69%, white powder. For analytical purposes, the compound was purified via the preparative HPLC method A. NMR data are provided for the TFA salt.

^{1}H NMR (400 MHz, DMSO-d_6) δ 9.63 (br s, 1H), 8.89 (br s, 1H), 7.42 – 7.27 (m, 5H), 5.04 (s, 2H), 3.46 (d, J = 11.5 Hz, 2H), 3.39 (t, J = 6.3 Hz, 2H), 3.15 – 2.90 (m, 8H), 1.67 – 1.54 (m, 2H), 1.48 – 1.37 (m, 2H), 1.37 – 1.23 (m, 2H).

^{13}C{^{1}H} NMR (101 MHz, DMSO-d_6) δ 154.8, 136.8, 128.4, 128.0, 127.9, 65.5, 60.3, 55.4, 51.1, 50.7, 31.8, 23.2, 22.6.

HRMS (ESI) m/z: $[M+H]^+$ calcd for $C_{17}H_{27}N_3O_3+H^+$: 322.2125; found: 322.2134.

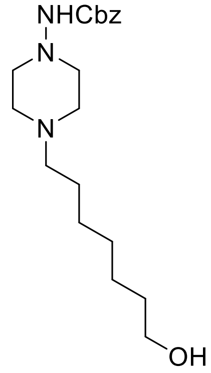

benzyl (4-(7-hydroxypentyl)piperazin-1-yl)carbamate (20). The compound was prepared according to General Procedure B, starting from piperazine **17** (1 g, 2.16 mmol) and 7-bromopropanol (0.95 g, 4.32 mmol). Yield: 0.867 g, 79%, white powder. For analytical purposes, the compound was purified via the preparative HPLC method A. NMR data are provided for the TFA salt.

^{1}H NMR (400 MHz, DMSO-d_6) δ 9.75 (br s, 1H), 8.89 (br s, 1H), 7.42 – 7.27 (m, 5H), 5.04 (s, 2H), 3.46 (d, J = 11.5 Hz, 2H), 3.37 (t, J = 6.5 Hz, 2H), 3.16 – 2.89 (m, 8H), 1.64 – 1.54 (m, 2H), 1.44 – 1.36 (m, 2H), 1.34 – 1.22 (m, 6H).

^{13}C{^{1}H} NMR (101 MHz, DMSO-d_6) δ 154.8, 136.8, 128.4, 128.0, 127.9, 65.5, 60.6, 55.3, 51.2, 50.7, 32.4, 28.4, 26.0, 25.3, 23.3.

HRMS (ESI) m/z: $[M+H]^+$ calcd for $C_{19}H_{31}N_3O_3+H^+$: 350.2438; found: 350.2457.

General Procedure C (esterification of catecholates with piperazine-containing alcohols). The alcohol (0.343 mmol) and the protected catechol (0.343 mmol) were mixed together in dry DCM prior to the addition of DIC (161 µL, 1.02 mmol) and DMAP (4.2 mg, 0.034 mmol). The obtained mixture was left stirring under an Ar atmosphere overnight. The following day, the solvent was evaporated, and the residue was extracted with water (10 mL) and MTBE (20 mL). The organic layer was separated, washed with sat. $NaHCO_3$ (10 mL), water (10 mL), brine (5 mL), dried over anhydrous Na_2SO_4, and concentrated under reduced pressure. The obtained crude was dissolved in THF (10 mL), followed by the addition of Pd/C (25 mg). The reaction mixture was left stirring overnight under an H_2 atmosphere (1 bar, balloon).

The next day, the suspension was filtered, and the filtrate was concentrated in vacuo to obtain the desired product.

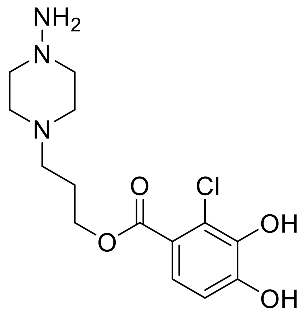

3-(4-aminopiperazin-1-yl)propyl 2-chloro-3,4-dihydroxybenzoate (27). The compound was prepared according to General Procedure C, starting from alcohol **18** (100 mg, 0.343 mmol) and catechol **25** (126 mg, 0.343 mmol). Yield: 52 mg, 46%, white powder. For analytical purposes, the compound was purified via the preparative HPLC method A. NMR data are provided for the TFA salt.

^{1}H NMR (400 MHz, DMSO-d_6) δ 10.81 (br s, 1H), 9.84 (br s, 3H), 9.45 (br s, 1H), 7.29 (d, *J* = 8.5 Hz, 1H), 6.82 (d, *J* = 8.5 Hz, 1H), 4.25 (t, *J* = 5.9 Hz, 3H), 3.85 – 2.80 (m, 10H), 2.12 – 2.00 (m, 2H).

^{13}C{^{1}H} NMR (101 MHz, DMSO-d_6) δ 164.8, 150.3, 142.8, 122.8, 120.3, 120.1, 112.9, 61.8, 52.8, 50.4, 50.2, 23.2.

HRMS (ESI) m/z: [M+H]$^+$ calcd for $C_{14}H_{20}ClN_3O_4$+H$^+$: 330.1215; found: 330.1218.

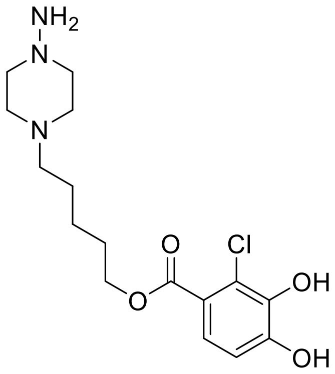

5-(4-aminopiperazin-1-yl)propyl 2-chloro-3,4-dihydroxybenzoate (28). The compound was prepared according to General Procedure C, starting from alcohol **19** (110 mg, 0.343 mmol) and catechol **25** (126 mg, 0.343 mmol). Yield: 61 mg, 50%, white powder. For analytical purposes, the compound was purified via the preparative HPLC method A. NMR data are provided for the TFA salt.

^{1}H NMR (400 MHz, DMSO-d_6) δ 10.76 (br s, 1H), 9.89 (br s, 3H), 9.44 (s, 1H), 7.22 (d, *J* = 8.5 Hz, 1H), 6.82 (d, *J* = 8.5 Hz, 1H), 4.19 (t, *J* = 6.3 Hz, 2H), 3.59 (br s, 2H), 3.32 (br s, 2H), 3.20 – 3.01 (m, 4H), 2.96 (s, 2H), 1.76 – 1.60 (m, 4H), 1.40 (p, *J* = 7.6 Hz, 2H).

^{13}C{^{1}H} NMR (101 MHz, DMSO-d_6) δ 165.1, 150.1, 142.8, 122.4, 120.7, 120.1, 112.9, 64.1, 55.1, 50.2, 50.0, 27.6, 22.9, 22.6.

HRMS (ESI) m/z: [M+H]$^+$ calcd for $C_{16}H_{24}ClN_3O_4$+H$^+$: 358.1528; found: 358.1537.

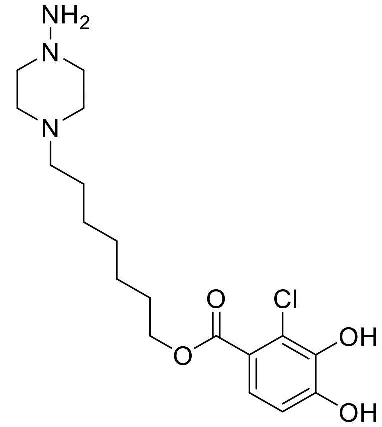

5-(4-aminopiperazin-1-yl)propyl 2-chloro-3,4-dihydroxybenzoate (29). The compound was prepared according to General Procedure C, starting from alcohol **20** (120 mg, 0.343 mmol) and catechol **25** (126 mg, 0.343 mmol). Yield: 53 mg, 40%, white powder. For analytical purposes, the compound was purified via the preparative HPLC method A. NMR data are provided for the TFA salt.

^{1}H NMR (400 MHz, DMSO-*d$_6$*) δ 10.71 (br s, 1H), 9.85 (br s, 3H), 9.43 (br s, 1H), 7.20 (d, *J* = 8.5 Hz, 1H), 6.81 (d, *J* = 8.5 Hz, 1H), 4.18 (t, *J* = 6.5 Hz, 2H), 3.55 (br s, 2H), 3.32 (br s, 2H), 3.19 – 3.00 (m, 4H), 2.95 (br s, 2H), 1.72 – 1.53 (m, 4H), 1.44 – 1.20 (m, 6H).

^{13}C{^{1}H} NMR (101 MHz, DMSO-*d$_6$*) δ 165.2, 150.0, 142.7, 122.3, 120.8, 120.1, 112.9, 64.5, 55.2, 50.2, 50.0, 28.1, 28.0, 25.9, 25.3, 23.2.

HRMS (ESI) m/z: $[M+H]^+$ calcd for $C_{18}H_{28}ClN_3O_4+H^+$: 386.1841; found: 386.1853.

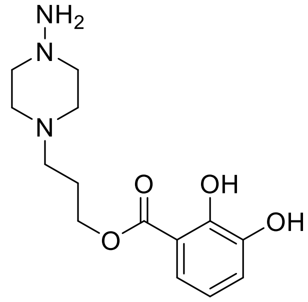

3-(4-aminopiperazin-1-yl)propyl 2,3-dihydroxybenzoate (30). The compound was prepared according to General Procedure C, starting from alcohol **18** (100 mg, 0.343 mmol) and catechol **26** (126 mg, 0.343 mmol). Yield: 14 mg, 14%, white powder. For analytical purposes, the compound was purified via the preparative HPLC method A. NMR data are provided for the TFA salt.

^{1}H NMR (400 MHz, DMSO-*d$_6$*) δ 10.33 (s, 1H), 9.98 (br s, 1H), 9.65 (br s, 2H), 9.53 (br s, 1H), 7.27 (dd, *J* = 8.0, 1.6 Hz, 1H), 7.04 (dd, *J* = 7.8, 1.6 Hz, 1H), 6.75 (t, *J* = 7.9 Hz, 1H), 4.36 (t, *J* = 5.9 Hz, 2H), 3.41 – 3.04 (m, 8H), 2.92 (br s, 2H), 2.18 – 2.01 (m, 2H).

^{13}C{^{1}H} NMR (101 MHz, DMSO-*d$_6$*) δ 169.2, 149.4, 146.1, 120.7, 119.8, 118.9, 113.3, 62.4, 52.7, 50.2, 23.1.

HRMS (ESI) m/z: $[M+H]^+$ calcd for $C_{14}H_{21}N_3O_4+H^+$: 296.1605; found: 296.1612.

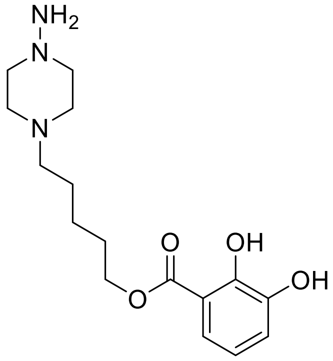

5-(4-aminopiperazin-1-yl)propyl 2,3-dihydroxybenzoate (31). The compound was prepared according to General Procedure C, starting from alcohol **19** (110 mg, 0.343 mmol) and catechol **26** (114 mg, 0.343 mmol). Yield: 64 mg, 58%, white powder. For analytical purposes, the compound was purified via the preparative HPLC method A. NMR data are provided for the TFA salt.

^{1}H NMR (400 MHz, DMSO-d_6) δ 10.46 (s, 1H), 9.92 (br s, 3H), 9.57 (br s, 1H), 7.23 (dd, *J* = 8.0, 1.6 Hz, 1H), 7.04 (dd, *J* = 7.8, 1.6 Hz, 1H), 6.74 (t, *J* = 7.9 Hz, 1H), 4.30 (t, *J* = 6.4 Hz, 2H), 3.58 (br s, 2H), 3.33 (br s, 2H), 3.2 – 3.05 (m, 4H), 2.96 (br s, 2H), 1.79 – 1.61 (m, 4H), 1.41 (p, *J* = 7.6 Hz, 2H).

^{13}C{^{1}H} NMR (101 MHz, DMSO-d_6) δ 169.6, 149.6, 146.2, 120.8, 119.5, 119.0, 113.2, 64.7, 55.1, 50.2, 50.0, 27.5, 22.9, 22.5.

HRMS (ESI) m/z: $[M+H]^+$ calcd for $C_{16}H_{25}N_3O_4+H^+$: 324.1918; found: 324.1928.

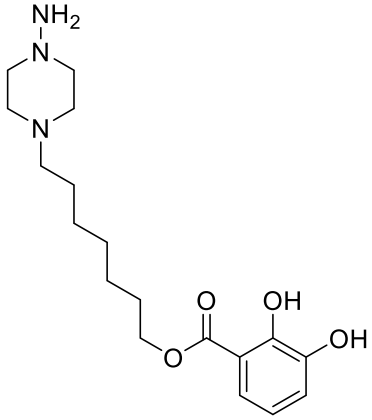

7-(4-aminopiperazin-1-yl)propyl 2,3-dihydroxybenzoate (32). The compound was prepared according to General Procedure C, starting from alcohol **20** (120 mg, 0.343 mmol) and catechol **26** (114 mg, 0.343 mmol). Yield: 49 mg, 41%, white powder. For analytical purposes, the compound was purified via the preparative HPLC method A. NMR data are provided for the TFA salt.

^{1}H NMR (400 MHz, DMSO-d_6) δ 10.48 (s, 1H), 9.86 (s, 3H), 9.52 (s, 1H), 7.22 (dd, *J* = 8.0, 1.6 Hz, 1H), 7.04 (dd, *J* = 7.8, 1.5 Hz, 1H), 6.75 (t, *J* = 7.9 Hz, 1H), 4.30 (t, *J* = 6.5 Hz, 2H), 3.57 (br s, 2H), 3.39 – 3.21 (m, 2H), 3.18 – 3.00 (m, 4H), 2.95 (br s, 2H), 1.71 (p, *J* = 6.6 Hz, 2H), 1.66 – 1.56 (m, 2H), 1.44 – 1.23 (m, 6H).

^{13}C{^{1}H} NMR (101 MHz, DMSO-d_6) δ 169.6, 149.6, 146.2, 120.8, 119.4, 119.0, 113.1, 65.1, 55.2, 50.2, 50.0, 28.1, 27.9, 25.8, 25.2, 23.2.

HRMS (ESI) m/z: $[M+H]^+$ calcd for $C_{18}H_{29}N_3O_4+H^+$: 352.2231; found: 352.2242.

Synthesis of amide S12

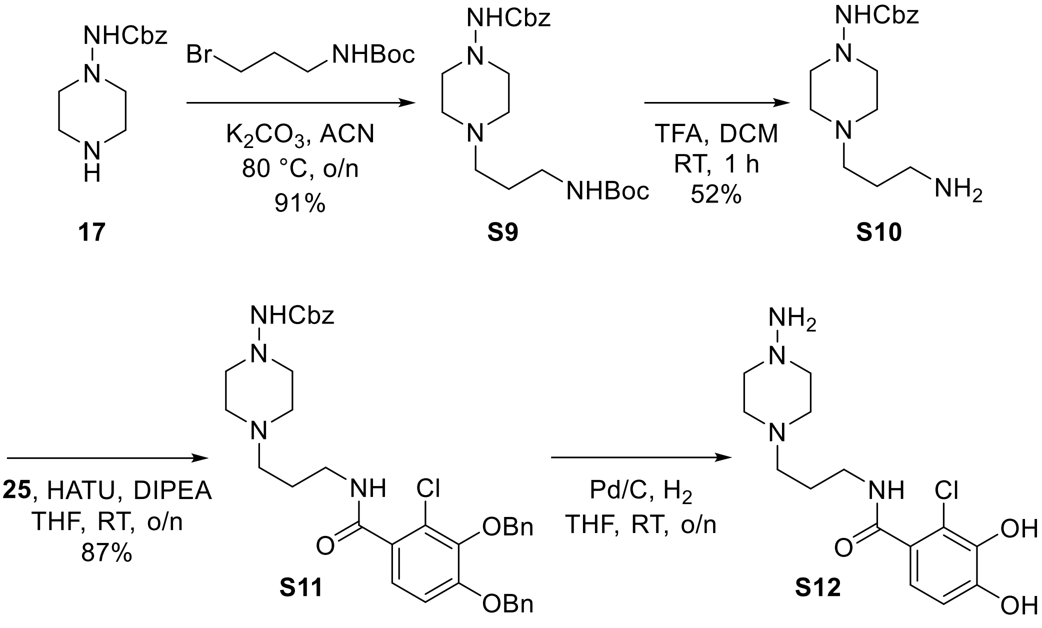

Scheme S3. *Synthesis of the amide* ***S12****.*

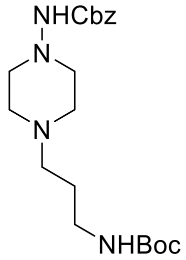

benzyl (4-(3-((tert-butoxycarbonyl)amino)propyl)piperazin-1-yl)carbamate (S9). tert-Butyl (3-bromopropyl)carbamate (284 mg, 1.19 mmol) was added to a stirred suspension of piperazine **17** (500 mg, 1.08 mmol) and K_2CO_3 (750 mg, 5.40 mmol) in ACN (10 mL), and the mixture was left stirring at 80°C overnight. The next day, the solvent was evaporated, and the residue was redissolved in water (20 mL), followed by extraction with MTBE (2 × 25 mL). The combined organic layers were washed with brine (15 mL), dried over anhydrous Na_2SO_4, and concentrated under reduced pressure. The residue was purified via column chromatography (SiO_2, DCM/MeOH/Et_3N = 99:1:0.1, R_f = 0.3) to afford compound **S9** (386 mg, 91% yield) as a white powder.

^{1}H NMR (400 MHz, $CDCl_3$) δ 7.38 – 7.27 (m, 5H), 5.64 (br s, 1H), 5.26 (br s, 1H), 5.12 (s, 2H), 3.17 (q, *J* = 6.0 Hz, 2H), 2.83 (br s, 4H), 2.58 (br s, 4H), 2.42 (t, *J* = 6.8 Hz, 2H), 1.63 (p, *J* = 6.7 Hz, 2H), 1.42 (s, 9H).

^{13}C{^{1}H} NMR (101 MHz, $CDCl_3$) δ 156.2, 136.3, 128.7, 128.4, 67.1, 56.3, 56.1, 52.4, 39.8, 28.6, 26.7.

HRMS (ESI) m/z: $[M+H]^+$ calcd for $C_{20}H_{32}N_4O_4+H^+$: 393.2497; found: 393.2512.

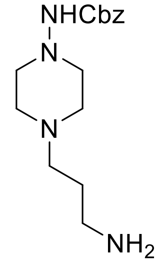

benzyl (4-(3-aminopropyl)piperazin-1-yl)carbamate (S10). TFA (5 mL) was poured into the solution of compound **S9** (386 mg, 0.985 mmol) in DCM (5 mL), and the reaction was left stirring at RT for 1 h. After that, the mixture was evaporated, and the residue was triturated with THF (5 mL) to obtain the precipitate, which was filtered and washed with MTBE (2 × 5 mL) to afford TFA salt of the compound **S10** (267 mg, 52% yield, calculated for 2 × TFA salt) as a white powder.

^{1}H NMR (400 MHz, DMSO-*d*$_6$) δ 10.13 (br s, 1H), 8.91 (br s, 1H), 8.01 (s, 3H), 7.42 – 7.27 (m, 5H), 5.04 (s, 2H), 3.54 – 3.34 (m, 2H), 3.22 – 2.77 (m, 10H), 1.92 (p, *J* = 8.3 Hz, 2H).

^{13}C{^{1}H} NMR (101 MHz, DMSO-*d*$_6$) δ 154.8, 136.8, 128.4, 128.0, 127.9, 65.5, 52.6, 51.2, 50.8, 36.3, 21.8.

HRMS (ESI) m/z: [M+H]$^+$ calcd for $C_{15}H_{24}N_4O_2$+H$^+$: 293.1972; found: 293.1982.

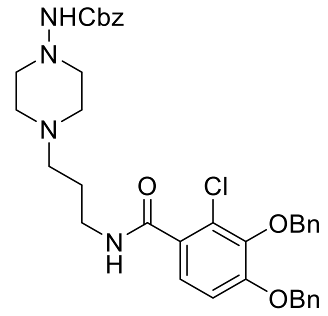

benzyl (4-(3-(3,4-bis(benzyloxy)-2-chlorobenzamido)propyl)piperazin-1-yl)carbamate (S11). The amine **S10** (262 mg, 0.503 mmol), the protected catechol **25** (186 mg, 0.503 mmol), and DIPEA (880 µl, 5.03 mmol) were mixed together in dry DCM prior to the addition of HATU (191 mg, 0.503 mmol). The obtained mixture was left stirring under an Ar atmosphere overnight. The following day, the solvent was evaporated, and the residue was diluted with water (10 mL) and MTBE (10 mL) to form the precipitate, which was filtered, washed with MTBE (10 mL), PE (10 mL), and dried to afford compound **S11** (281 mg, 87% yield) as an off-yellow powder.

^{1}H NMR (400 MHz, $CDCl_3$) δ 7.48 – 7.28 (m, 17H), 6.93 (d, *J* = 8.7 Hz, 1H), 5.52 (br s, 1H), 5.15 (s, 2H), 5.10 (s, 2H), 5.03 (s, 2H), 3.53 (q, *J* = 5.9 Hz, 2H), 2.88 – 2.54 (m, 8H), 2.51 (t, *J* = 6.3 Hz, 2H), 1.76 (p, *J* = 6.2 Hz, 2H).

^{13}C{^{1}H} NMR (101 MHz, $CDCl_3$) δ 166.3, 154.3, 144.9, 136.9, 136.1, 129.4, 128.8, 128.7, 128.7, 128.5, 128.4, 128.4, 127.6, 126.2, 125.4, 112.3, 75.1, 71.1, 67.0, 56.8, 56.0, 52.4, 39.8, 25.5.

HRMS (ESI) m/z: [M+H]$^+$ calcd for $C_{36}H_{39}ClN_4O_5$+H$^+$: 643.2682; found: 643.2702.

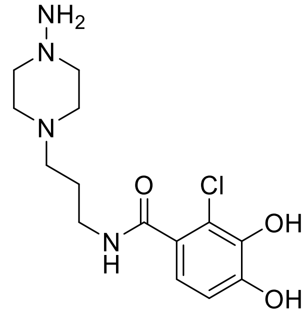

***N*-(3-(4-aminopiperazin-1-yl)propyl)-2-chloro-3,4-dihydroxybenzamide (S12)**. The amide **S11** (281 mg, 0.437 mmol) was dissolved in THF (20 mL), followed by the addition of Pd/C (50 mg). The reaction mixture was left stirring for 2 days under an H_2 atmosphere (1 bar, balloon). The next day, the suspension was filtered, and the filtrate was concentrated in vacuo to obtain compound **S12** (142 mg, 99% yield, unpurified) as a white powder that was used in the next step without further purification.

HRMS (ESI) m/z: $[M+H]^+$ calcd for $C_{14}H_{21}ClN_4O_3+H^+$: 329.1375; found: 329.1378.

Synthesis of alcohols 21-23, ester-linked conjugates 33-38, and amide 39

General Procedure D (conjugation of piperazine-containing alcohols with rifaldehyde. The alcohol (0.028 mmol) was dissolved in THF (5 mL), followed by the addition of Pd/C (10 mg). The reaction mixture was left stirring overnight under an H_2 atmosphere (1 bar, balloon). The next day, the suspension was filtered, and the filtrate was concentrated and directly dissolved in THF (5 mL). Rifaldehyde (10 mg, 0.014 mmol) was added, and the resulting solution was left stirring for 2 h. After that, the reaction mixture was evaporated and then directly purified using the preparative HPLC method B. The fractions containing the pure product, as judged by HPLC, were combined and lyophilized to yield the desired product.

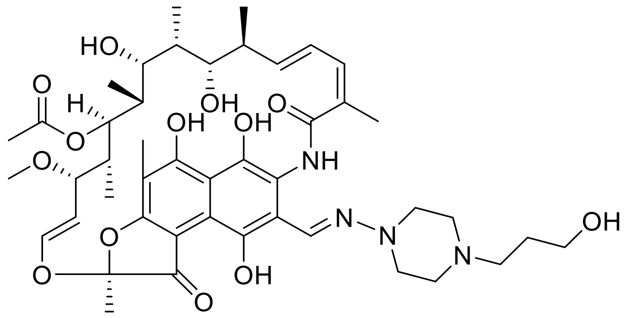

Compound 21. The compound was prepared according to General Procedure D, starting from alcohol **18** (4.5 mg, 0.028 mmol) and rifaldehyde (10 mg, 0.014 mmol). Yield: 3.4 mg, 28%, orange powder.

HRMS (ESI) m/z: $[M+H]^+$ calcd for $C_{45}H_{62}N_4O_{13}+H^+$: 867.4387; found: 867.4404.

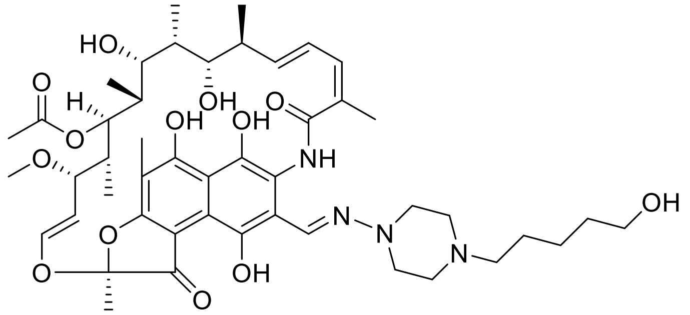

Compound 22. The compound was prepared according to General Procedure D, starting from alcohol **19** (5.3 mg, 0.028 mmol) and rifaldehyde (10 mg, 0.014 mmol). Yield: 4.4 mg, 35%, orange powder.

HRMS (ESI) m/z: $[M+H]^+$ calcd for $C_{47}H_{66}N_4O_{13}+H^+$: 895.4699; found: 895.4715.

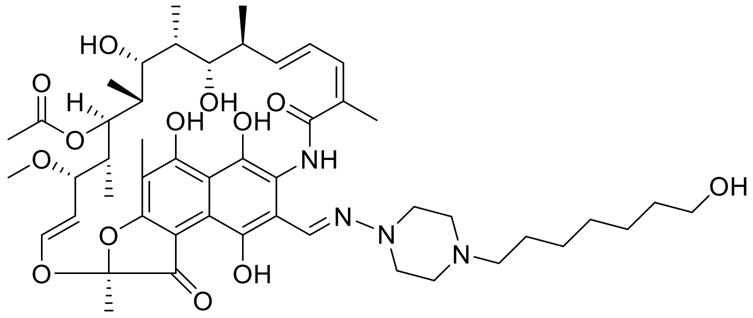

Compound 23. The compound was prepared according to General Procedure D, starting from alcohol **20** (6.1 mg, 0.028 mmol) and rifaldehyde (10 mg, 0.014 mmol). Yield: 4.4 mg, 34%, orange powder.

HRMS (ESI) m/z: $[M+H]^+$ calcd for $C_{49}H_{70}N_4O_{13}+H^+$: 923.5012; found: 923.5012.

General Procedure E (conjugation of piperazine-catechols with rifaldehyde). Rifaldehyde (10 mg, 0.014 mmol) and piperazine-catechol (0.028 mmol) were dissolved in dry THF (1 mL), and the resulting solution was left stirring for 2 h. After that, the reaction mixture was evaporated and then directly purified using the preparative HPLC method B. The fractions containing the pure product, as judged by HPLC, were combined and lyophilized to yield the desired conjugate.

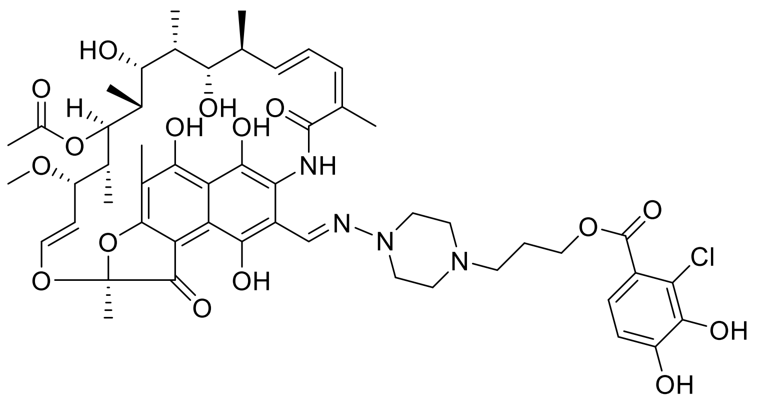

Conjugate 33. The compound was prepared according to General Procedure D, starting from piperazine-catechol **27** (9.2 mg, 0.028 mmol) and rifaldehyde (10 mg, 0.014 mmol). Yield: 7.6 mg, 52%, orange powder.

^{1}H NMR (600 MHz, DMSO-*d*$_6$) δ 12.38 (br s, 1H), 10.59 (br s, 1H), 10.17 (br s, 1H), 9.60 (br s, 1H), 9.43 (br s, 1H), 8.15 (s, 1H), 7.29 (d, *J* = 8.5 Hz, 1H), 6.98 – 6.88 (m, 1H), 6.81 (d, *J* = 8.5 Hz, 1H), 6.35 (d, *J* = 10.8 Hz, 1H), 6.26 (d, *J* = 12.7 Hz, 1H), 5.94 (dd, *J* = 15.8, 6.2 Hz, 1H), 5.05 (d, *J* = 10.9 Hz, 1H), 4.95 (dd, *J* = 12.8, 8.2 Hz, 1H), 4.27 (t, *J* = 6.1 Hz, 2H), 3.76 – 3.72 (m, 1H), 3.70 – 3.55 (m, 8H), 3.30 – 3.24 (m, 3H), 2.97 – 2.84 (m, 2H), 2.90 (s, 3H), 2.88 – 2.82 (m, 1H), 2.26 – 2.17 (m, 1H), 2.14 – 2.06 (m, 2H), 2.00 (s, 3H), 1.98 (s, 3H), 1.95 (s, 3H), 1.68 (s, 3H), 1.60 – 1.54 (m, 1H), 1.34 – 1.26 (m, 1H), 1.04 – 0.96 (m, 1H), 0.89 (d, *J* = 7.0 Hz, 3H), 0.82 (d, *J* = 6.9 Hz, 3H), 0.45 (d, *J* = 6.8 Hz, 3H), -0.33 (d, *J* = 6.7 Hz, 3H).

^{13}C{^{1}H} NMR (151 MHz, DMSO-*d*$_6$) δ 172.5, 169.5, 167.2, 164.8, 150.2, 146.2, 143.0, 142.8, 137.3, 133.1, 131.0, 122.8, 120.4, 120.2, 118.3, 117.2, 117.1, 116.5, 115.2, 115.1, 113.7, 112.8, 108.8, 103.2, 102.8, 76.2, 76.0, 73.4, 71.5, 61.8, 55.7, 52.9, 49.9, 49.3, 48.6, 47.6, 47.4, 40.1, 38.4, 38.0, 34.4, 32.8, 23.1, 21.9, 20.7, 20.4, 17.6, 11.2, 8.8, 8.6, 7.5.

HRMS (ESI) m/z: [M+H]$^+$ calcd for $C_{52}H_{65}ClN_4O_{16}+H^+$: 1037.4121; found: 1037.4161.

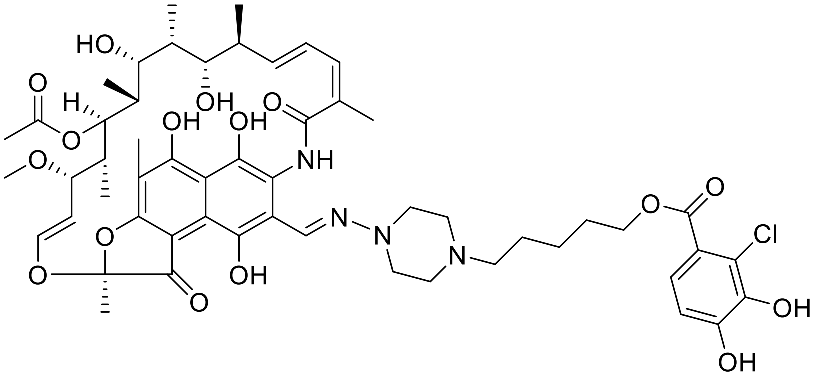

Conjugate 34. The compound was prepared according to General Procedure D, starting from piperazine-catechol **28** (10 mg, 0.028 mmol) and rifaldehyde (10 mg, 0.014 mmol). Yield: 6.8 mg, 46%, orange powder.

HRMS (ESI) m/z: [M+H]$^+$ calcd for $C_{54}H_{69}ClN_4O_{16}+H^+$: 1065.4470; found: 1065.4470.

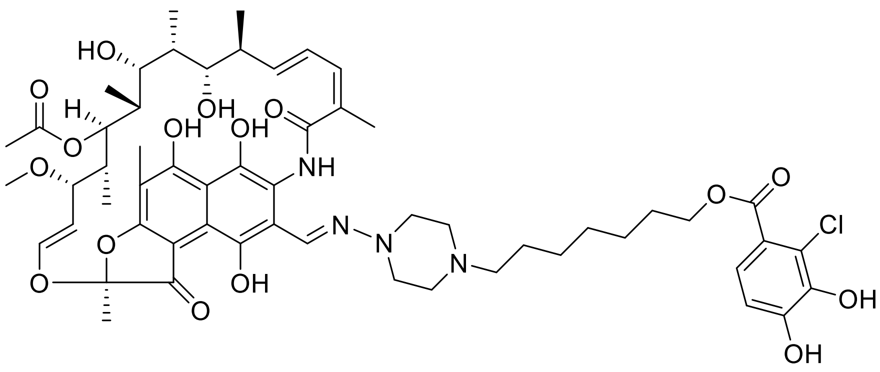

Conjugate 35. The compound was prepared according to General Procedure D, starting from piperazine-catechol **29** (10.8 mg, 0.028 mmol) and rifaldehyde (10 mg, 0.014 mmol). Yield: 5.8 mg, 38%, orange powder.

HRMS (ESI) m/z: $[M+H]^+$ calcd for $C_{56}H_{73}ClN_4O_{16}+H^+$: 1093.4783; found: 1093.4780.

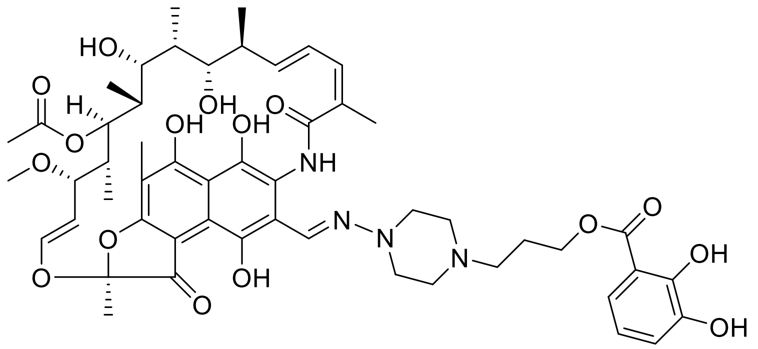

Conjugate 36. The compound was prepared according to General Procedure D, starting from piperazine-catechol **30** (8.2 mg, 0.028 mmol) and rifaldehyde (10 mg, 0.014 mmol). Yield: 5.1 mg, 36%, orange powder.

HRMS (ESI) m/z: $[M+H]^+$ calcd for $C_{52}H_{66}N_4O_{16}+H^+$: 1003.4547; found: 1003.4556.

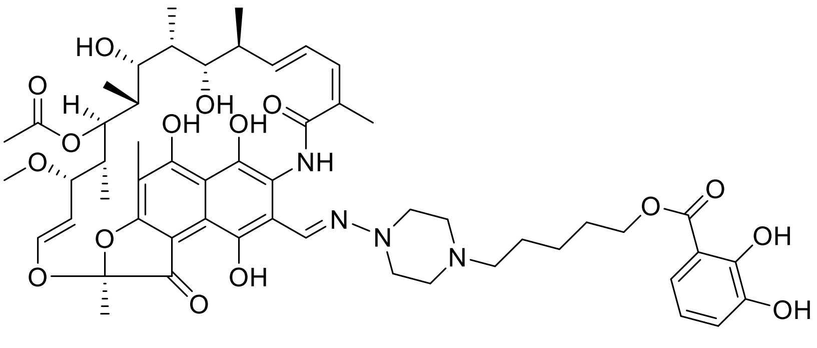

Conjugate 37. The compound was prepared according to General Procedure D, starting from piperazine-catechol **31** (9 mg, 0.028 mmol) and rifaldehyde (10 mg, 0.014 mmol). Yield: 4.7 mg, 33%, orange powder.

HRMS (ESI) m/z: $[M+H]^+$ calcd for $C_{54}H_{70}N_4O_{16}+H^+$: 1031.4860; found: 1031.4866.

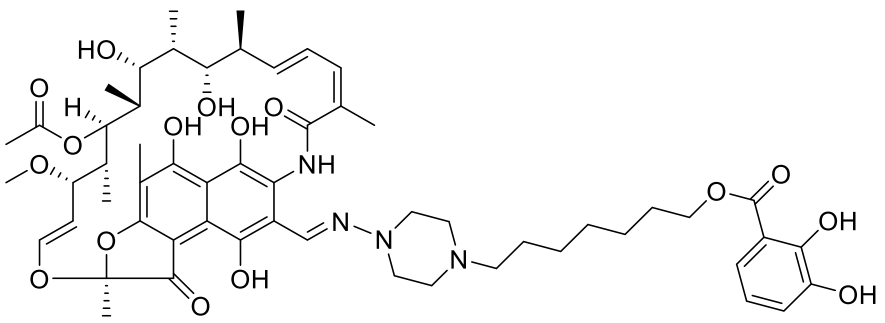

Conjugate 38. The compound was prepared according to General Procedure D, starting from piperazine-catechol **32** (9.8 mg, 0.028 mmol) and rifaldehyde (10 mg, 0.014 mmol). Yield: 4.4 mg, 30%, orange powder.

HRMS (ESI) m/z: $[M+H]^+$ calcd for $C_{56}H_{74}N_4O_{16}+H^+$: 1059.5173; found: 1059.5181.

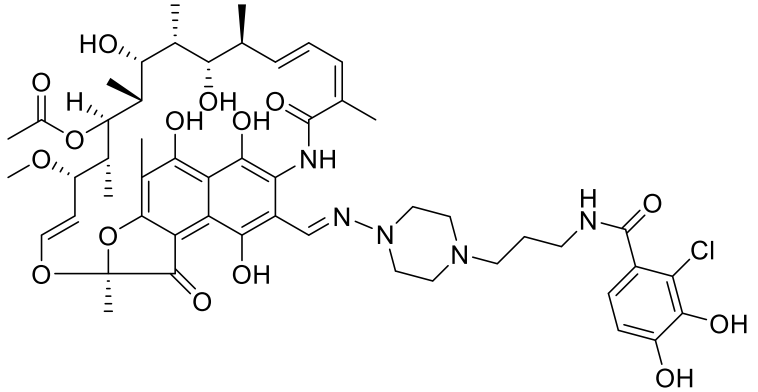

Conjugate 39. The compound was prepared according to General Procedure D, starting from piperazine-catechol **S12** (9.2 mg, 0.028 mmol) and rifaldehyde (10 mg, 0.014 mmol). Yield: 7.2 mg, 50%, orange powder.

HRMS (ESI) m/z: $[M+H]^+$ calcd for $C_{52}H_{66}ClN_5O_{15}+H^+$: 1036.4317; found: 1036.4310.

Synthesis of enterobactin

Enterobactin was synthesized following previously published methods with minor changes. Spectral data for all intermediates and enterobactin itself were in agreement with those reported in the literature.[15–18]

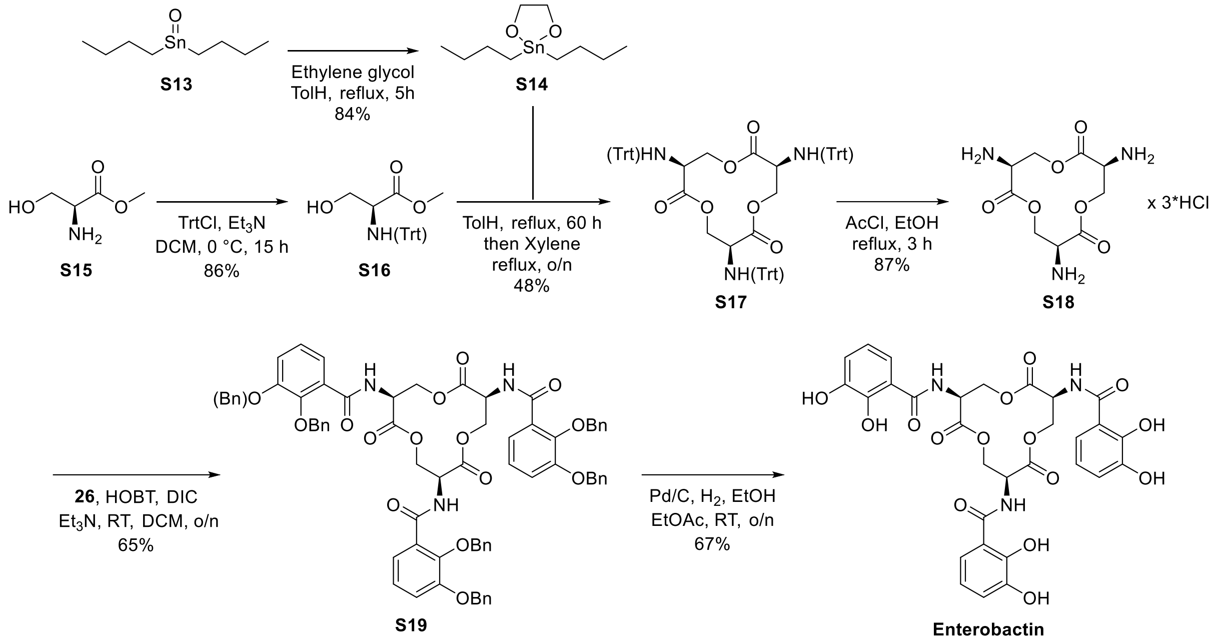

***Scheme S4**. Synthesis of enterobactin*

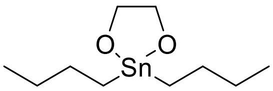

2,2-dibutyl-1,3,2-dioxastannolane (S14). Ethylene glycol (11.23 mL, 200 mmol) was added to a solution of dibutylstannanone (**S13**) (10 g, 40.2 mmol) in toluene (50 mL) at 25 °C. The reaction mixture was stirred at reflux for 5 hours. After gradually cooling from 110 °C to 20 °C, the precipitate was formed, filtered, washed with toluene, and dried at 40 °C under vacuum to give compound **S14** (9.9 g, 84% yield).

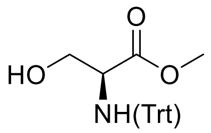

Methyl trityl-L-serinate (S16). To a suspension of L-Serine methyl ester hydrochloride (**S15**) (5 g, 32.2 mmol) in 50 mL of DCM at 0 °C, triethylamine (9 mL, 64.4 mmol) was added dropwise, followed by triphenylmethyl chloride (8.96 g, 32.2 mmol) in 20 mL of DCM. After stirring at RT for 15 h, the white precipitate formed was filtered off, and the filtrate evaporated in vacuo to yield a white solid, which was dissolved in EtOAc (200 mL) and washed with 10% citric acid (2 × 100 mL), saturated $NaHCO_3$ (100 mL), and water (2 × 100 mL). The separated organic layer was dried over Na_2SO_4, filtered, and concentrated in vacuo. The crude product was recrystallized from hexane to give compound **S16** (10.0 g, 86% yield) as a white solid.

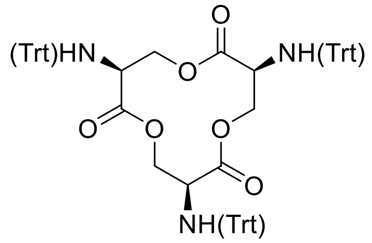

(3S,7S,11S)-3,7,11-tris(tritylamino)-1,5,9-trioxacyclododecane-2,6,10-trione (S17). Molecular sieves (20 g) and 2,2-dibutyl-1,3,2-dioxastannolane (**S14**) (0.41 g, 1.4 mmol) were added to a solution of methyl trityl-L-serine (**S16**) in 150 mL of dry toluene, and the mixture was stirred at reflux for 60 h. Then, toluene was evaporated, and the residue was dissolved in xylene and stirred overnight at reflux. The resulting mixture was cooled to RT, filtered through a pad of Celite, and washed with 100 mL of toluene. Then, the filtrate was discarded, and the celite was washed with warm DCM (5 × 150 mL). The filtrate was evaporated, and the crude was diluted with MTBE (25 mL). The precipitate was formed, filtered, and washed with MTBE (10 mL) to obtain compound **S17** (2.2 g, 48% yield).

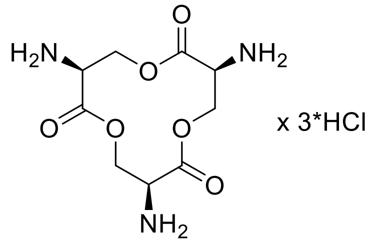

(3S,7S,11S)-3,7,11-triamino-1,5,9-trioxacyclododecane-2,6,10-trione trihydrochloride (S18). A dry solution of HCl was prepared by the reaction of acetyl chloride (0.35 mL, 4.86 mmol) with dry ethanol (30 mL). Then, a solution of the (3S,7S,11S)-3,7,11-tris(tritylamino)-1,5,9-trioxacyclododecane-2,6,10-trione (**S17**) (1.2 g, 1.21 mmol) in dry ethanol (10 mL) was added to the HCl solution and then refluxed for 30 minutes. The resulting mixture was concentrated to 15 mL under vacuum and cooled in an ice bath. The resulting solid was filtered, washed with cold, dry ethanol (5 mL), $CHCl_3$ (15 mL), and then Et_2O (2 × 15 mL), and dried to obtain compound **S18** (0.39 g, 87% yield) as a beige powder.

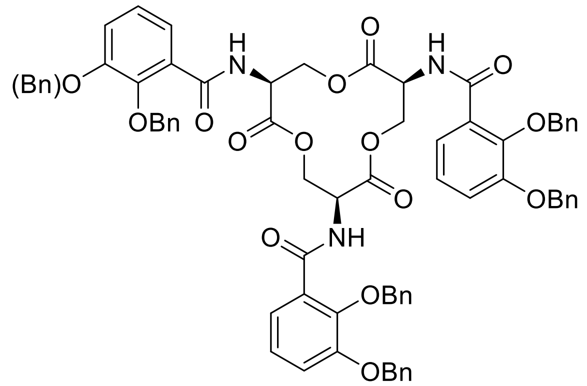

N,N',N''-((3S,7S,11S)-2,6,10-trioxo-1,5,9-trioxacyclododecane-3,7,11-triyl)-tris(2,3-bis(benzyloxy)-benzamide) (S19). (3S,7S,11S)-3,7,11-triamino-1,5,9-trioxacyclododecane-2,6,10-trione tris hydrochloride (**S18**) (0.38 g, 1.03 mmol) was suspended in anhydrous DCM (12 mL) and treated with dry triethylamine (0.5 mL, 4.61 mmol) under Ar. The reaction mixture was stirred at room temperature for 10 min. Then, in a separate round-bottom flask, a mixture of 2,3-bis(benzyloxy)benzoic acid (**26**) (1.2 g, 3.59 mmol) and HOBt × H_2O (0.785 g, 5.13 mmol) was dissolved in anhydrous DCM (20 mL), followed by addition of DIC (0.65 g, 5.13 mmol) and stirred at room temperature for 20 min under Ar. Then, the solution of triseryl amine was added to the activated benzoic acid and stirred overnight at RT. The mixture was diluted with 20 mL of DCM and 50 mL of water. After extraction, the organic layer was separated, washed with 1M HCl solution (25 mL), saturated $NaHCO_3$ solution (25 mL), water (25 mL),

and brine (15 mL), then dried over Na_2SO_4 and concentrated in vacuo. The resulting residue was purified by silica gel column chromatography (SiO_2, EtOAc/PE) to afford compound **S19** (0.8 g, 65% yield) as a colorless liquid.

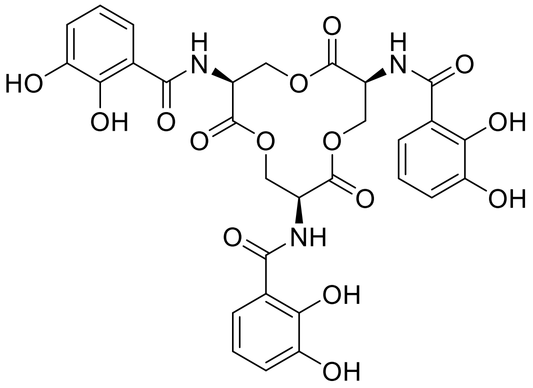

Enterobactin. N,N',N''-((3S,7S,11S)-2,6,10-trioxo-1,5,9-trioxacyclododecane-3,7,11-triyl)tris(2,3-bis(benzyloxy)benzamide) (**S19**) (0.75 g, 0.62 mmol) was dissolved in a mixture of EtOH (5 mL) and EtOAc (5 mL) and hydrogenated in a round bottom flask under balloon of hydrogen (1 bar) in presence of 10 % Pd/C (75 mg) overnight. The catalyst was filtered off, and the solvents were removed in vacuo to give the crude, which was purified by HPLC using method B to yield enterobactin (278 mg, 67% yield).

^{1}H NMR (400 MHz, DMSO-*d$_6$*) δ 11.62 (s, 3H), 9.44 (br s, 3H), 9.11 (d, J = 6.9 Hz, 3H), 7.33 (dd, J = 8.2, 1.4 Hz, 3H), 6.96 (dd, J = 7.8, 1.4 Hz, 3H), 6.73 (t, J = 8.0 Hz, 3H), 4.89 (ddd, J = 9.2, 6.9, 4.7 Hz, 3H), 4.68 – 4.59 (m, 3H), 4.39 (dd, J = 10.9, 4.5 Hz, 3H).

^{13}C{^{1}H} NMR (101 MHz, DMSO-*d$_6$*) δ 169.5, 169.0, 148.6, 146.2, 119.3, 118.5, 118.3, 115.29, 63.5, 51.3.

HRMS (ESI) m/z: $[M+H]^+$ calcd for $C_{30}H_{27}N_3O_{15}+H^+$: 670.1515; found: 670.1520.

Supplementary MIC results

Table S1. Activity of conjugate **33** and rifampicin in ID-CAMHB (iron-poor medium) against multiple wild-type *E. coli* BW 25113 strain and strains with different knockouts.

Strain	MIC (µg/mL)	
	**33**	Rifampicin
E. coli BW 25113	1	8
E. coli ΔtolC	1	4
E. coli ΔfepA	2	8
E. coli ΔfepB	1	4
E. coli ΔfepD	1	4
E. coli ΔfhuA	1	8
E. coli ΔfhuB	1	8
E. coli ΔfhuC	1	8
E. coli ΔfhuD	1	8
E. coli ΔfhuE	1	8
E. coli ΔfhuF	1	8
E. coli ΔentA	2	8
E. coli ΔentC	1	8

Hemolysis

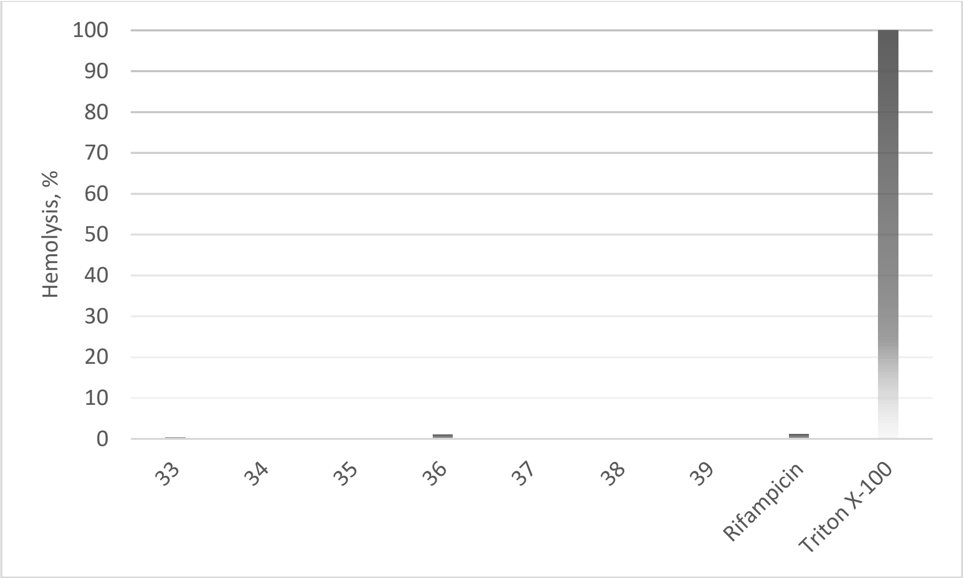

Figure S1. Hemolysis data (in %) for compounds **33-39** and rifampicin at a concentration of 64 µg/mL after 1 hour, compared to Triton X-100 (100%). The obtained values were used as the average of n = 3 technical replicates.

NMR Spectra

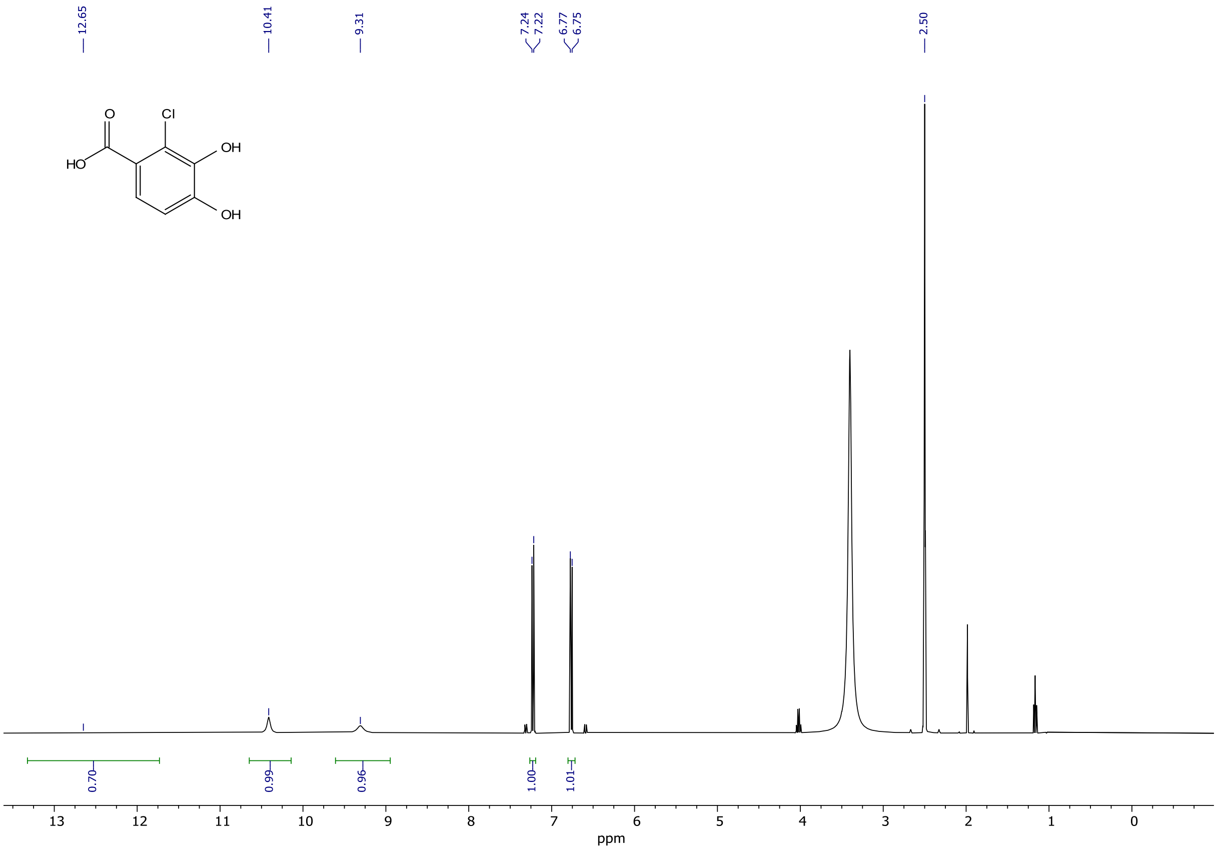

Figure S2. ^{1}H-NMR (400 MHz, DMSO-d_6) of compound **6**.

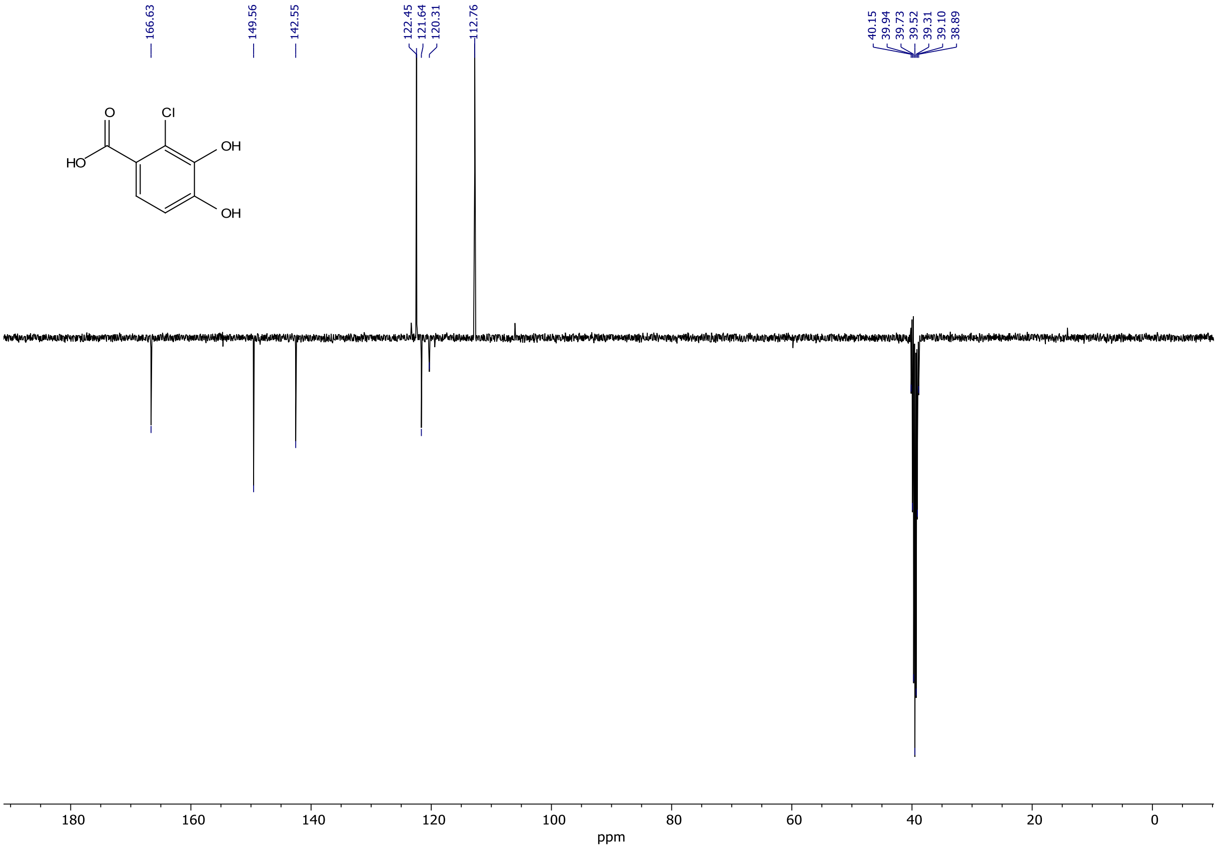

Figure S3. ^{13}C{^{1}H} NMR (101 MHz, DMSO-d_6) of the compound **6**.

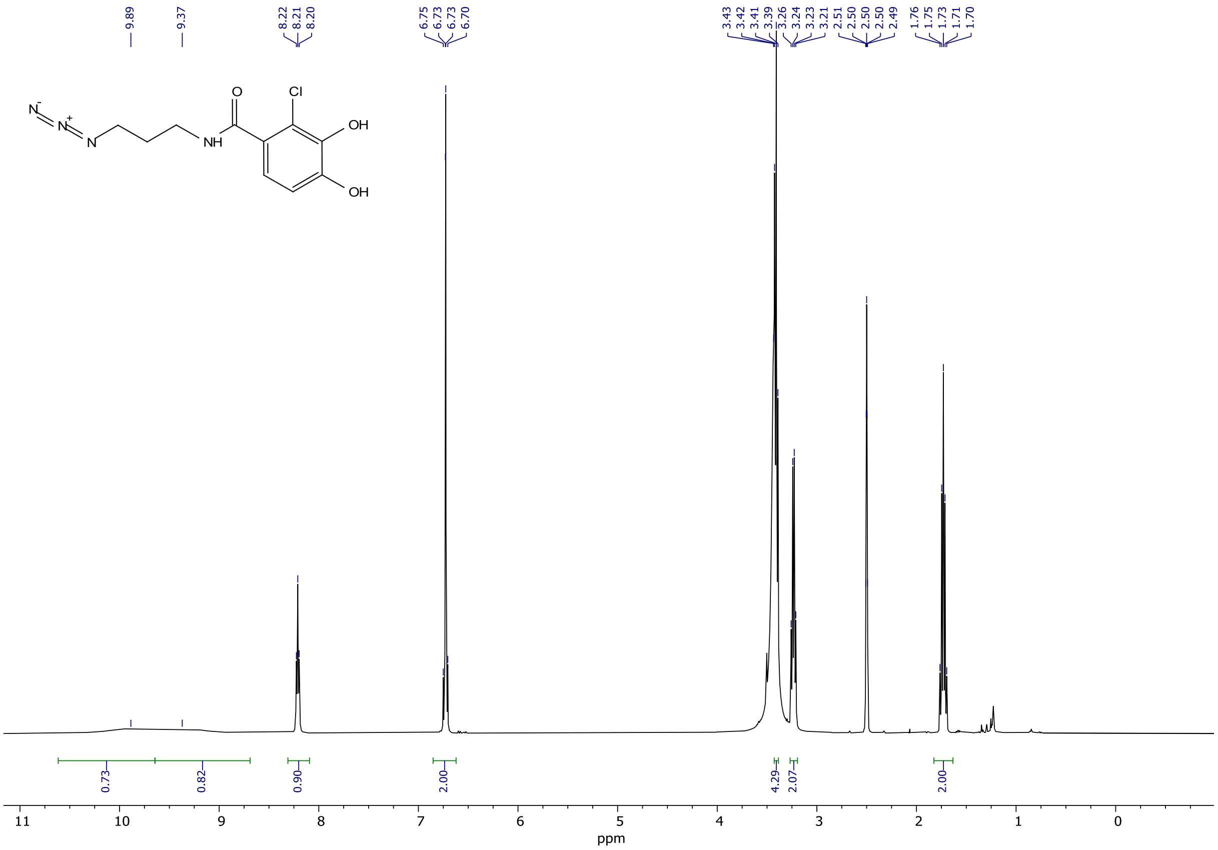

Figure S4. ^{1}H-NMR (400 MHz, DMSO-d_6) of compound **7**.

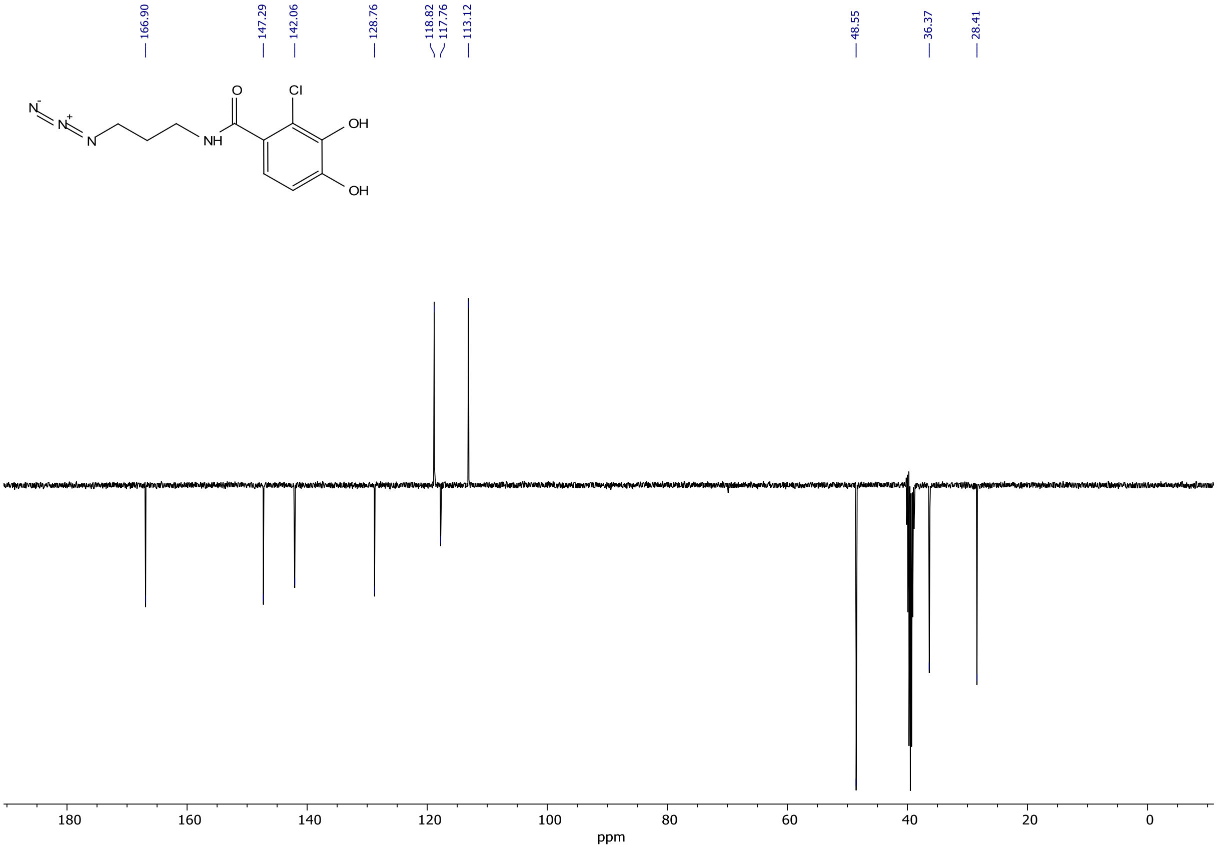

Figure S5. $^{13}C\{^{1}H\}$ NMR (101 MHz, DMSO-d_6) of the compound **7**.

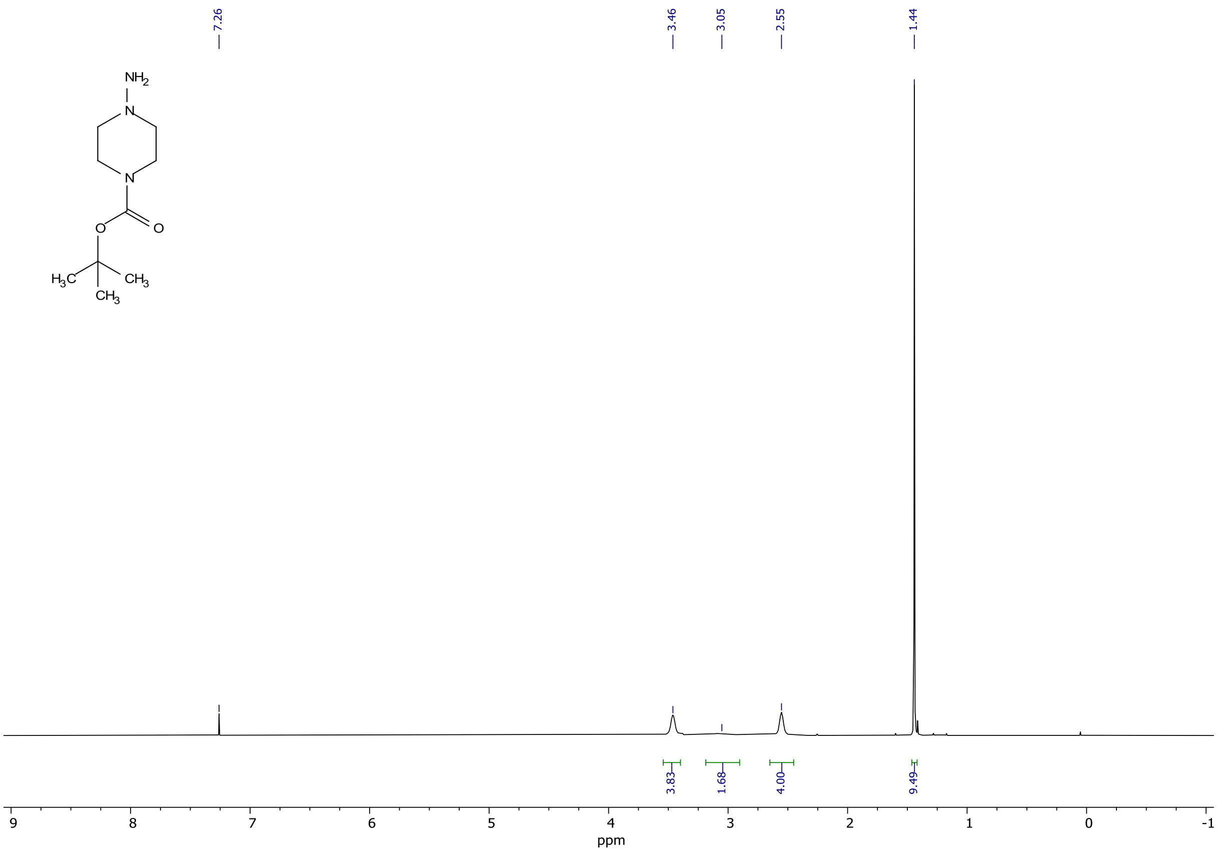

Figure S6. ^{1}H-NMR (400 MHz, $CDCl_3$) of compound **15**.

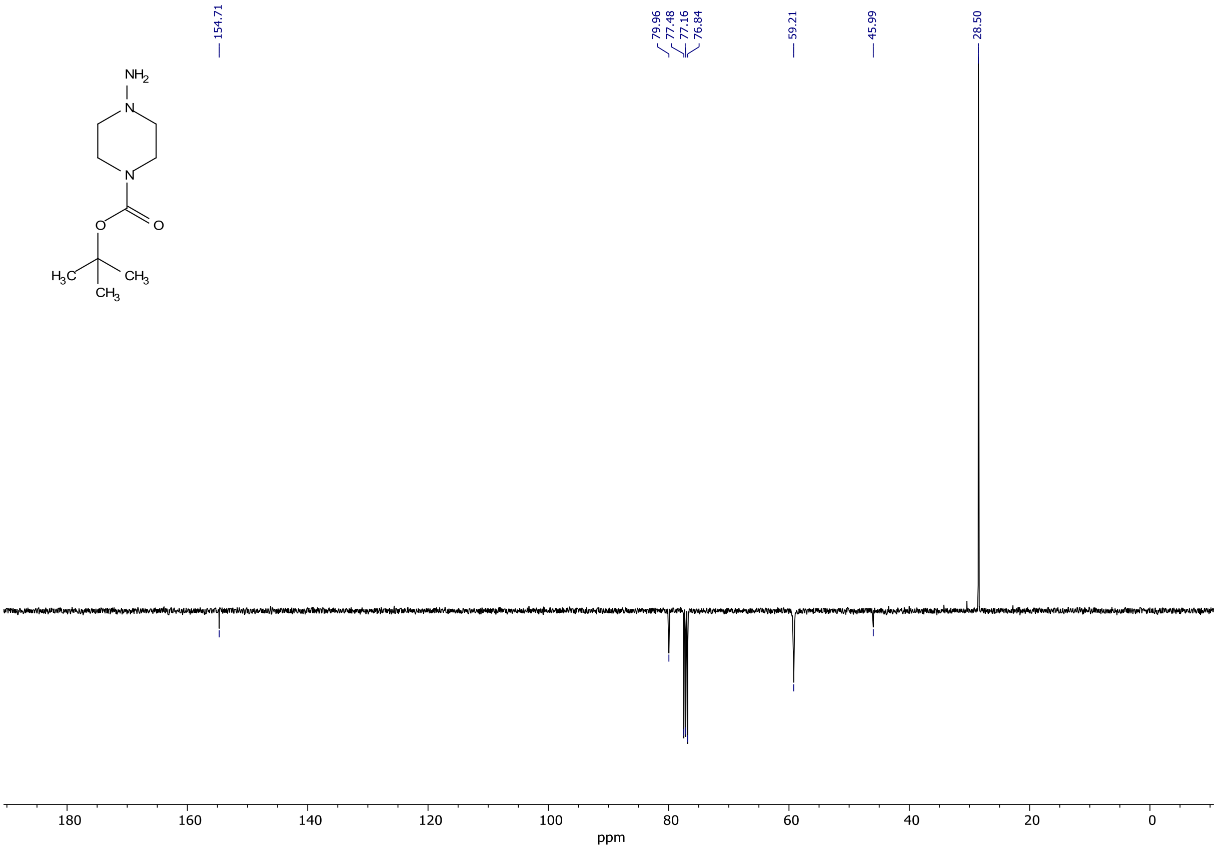

Figure S7. $^{13}C\{^{1}H\}$ NMR (101 MHz, $CDCl_3$) of the compound **15**.

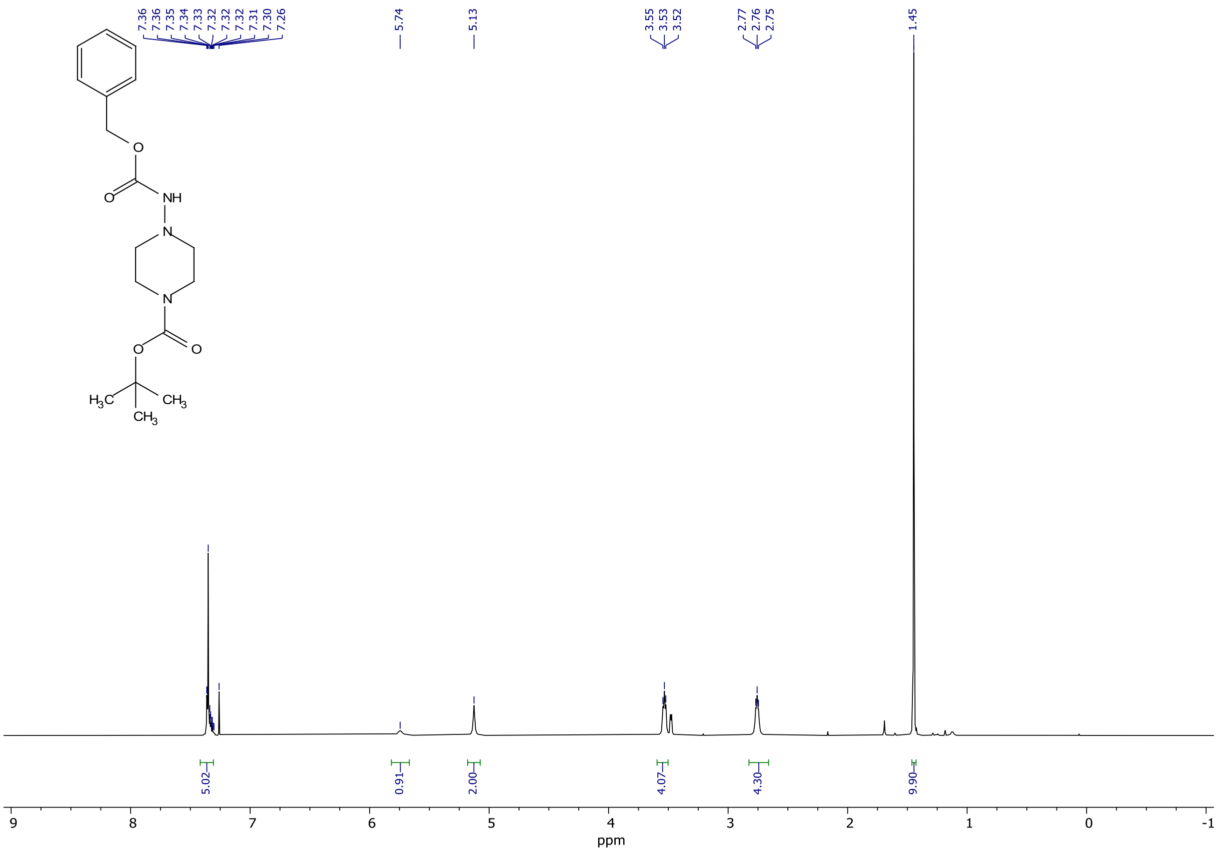

Figure S8. ^{1}H-NMR (400 MHz, $CDCl_3$) of compound **16**.

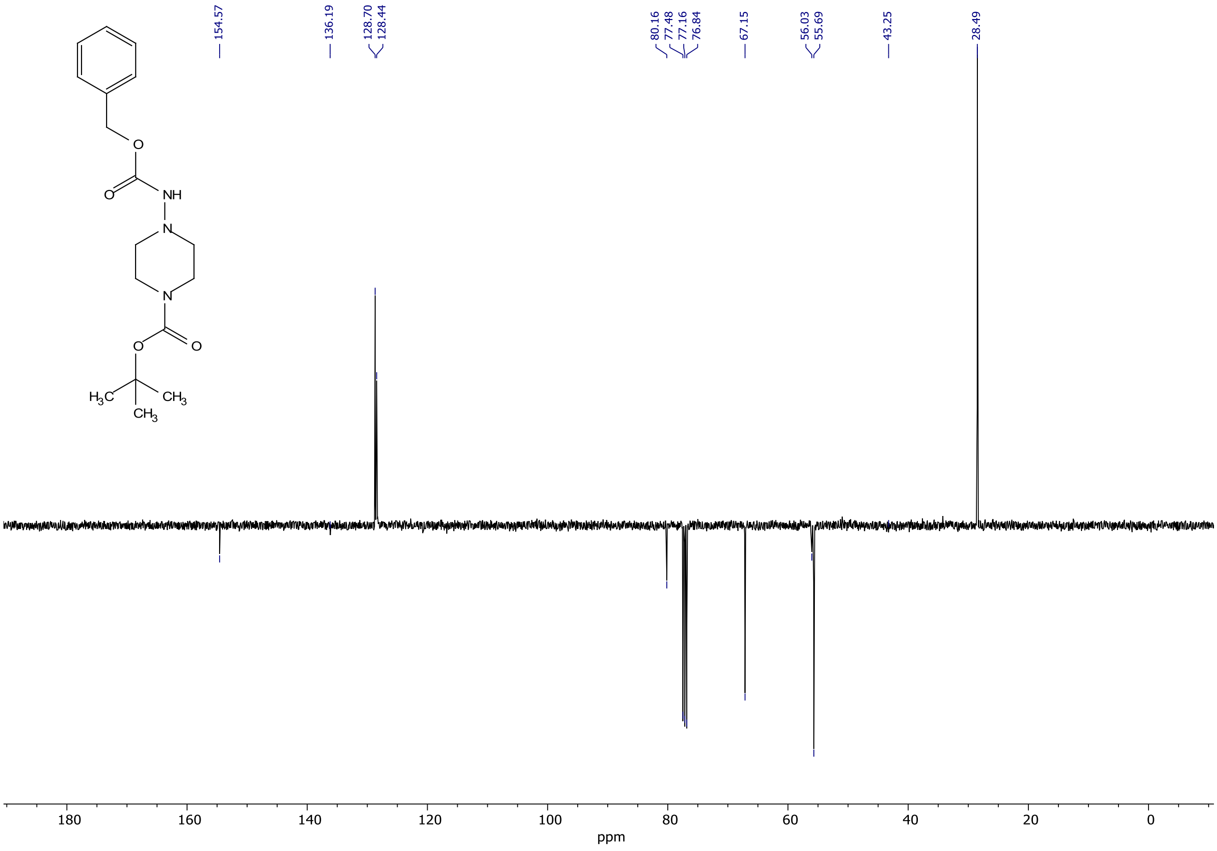

Figure S9. $^{13}C\{^{1}H\}$ NMR (101 MHz, $CDCl_3$) of the compound **16**.

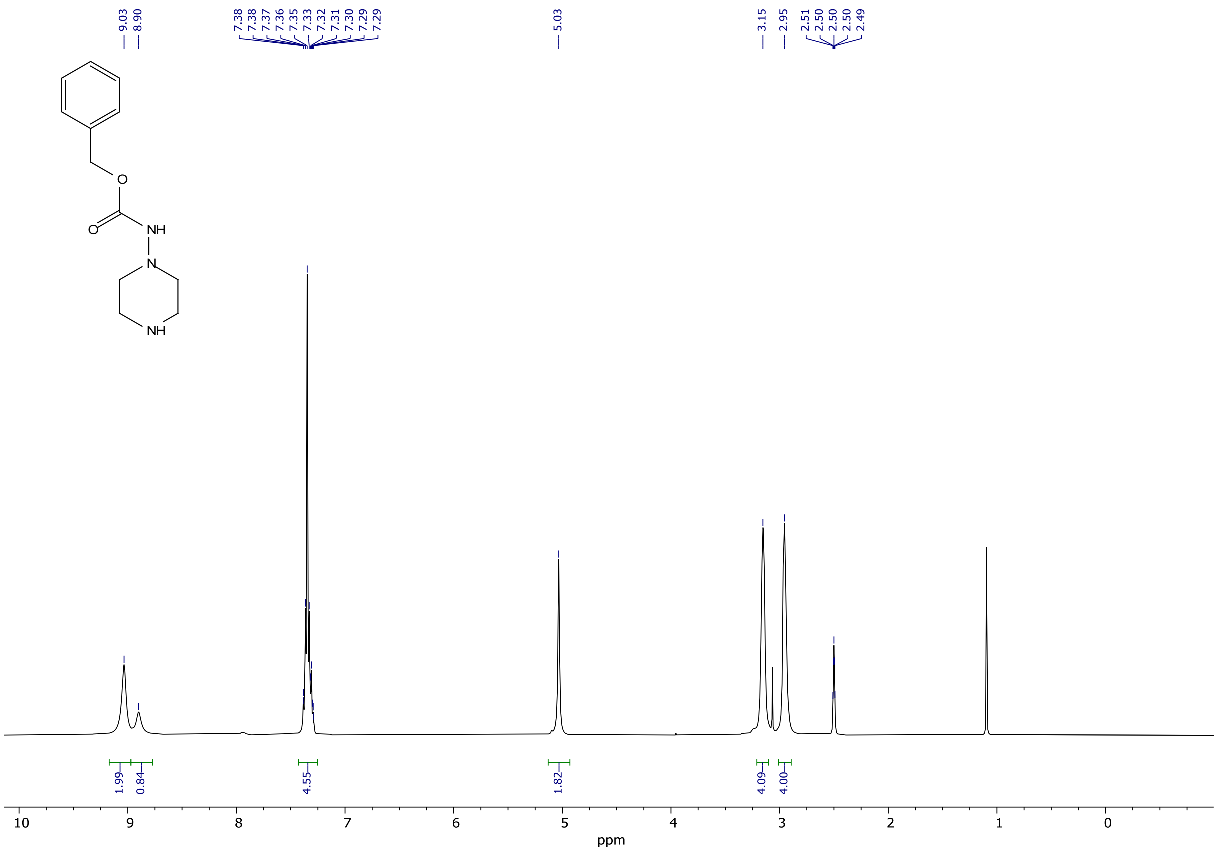

Figure S10. ^{1}H-NMR (400 MHz, DMSO-d_6) of compound **17**.

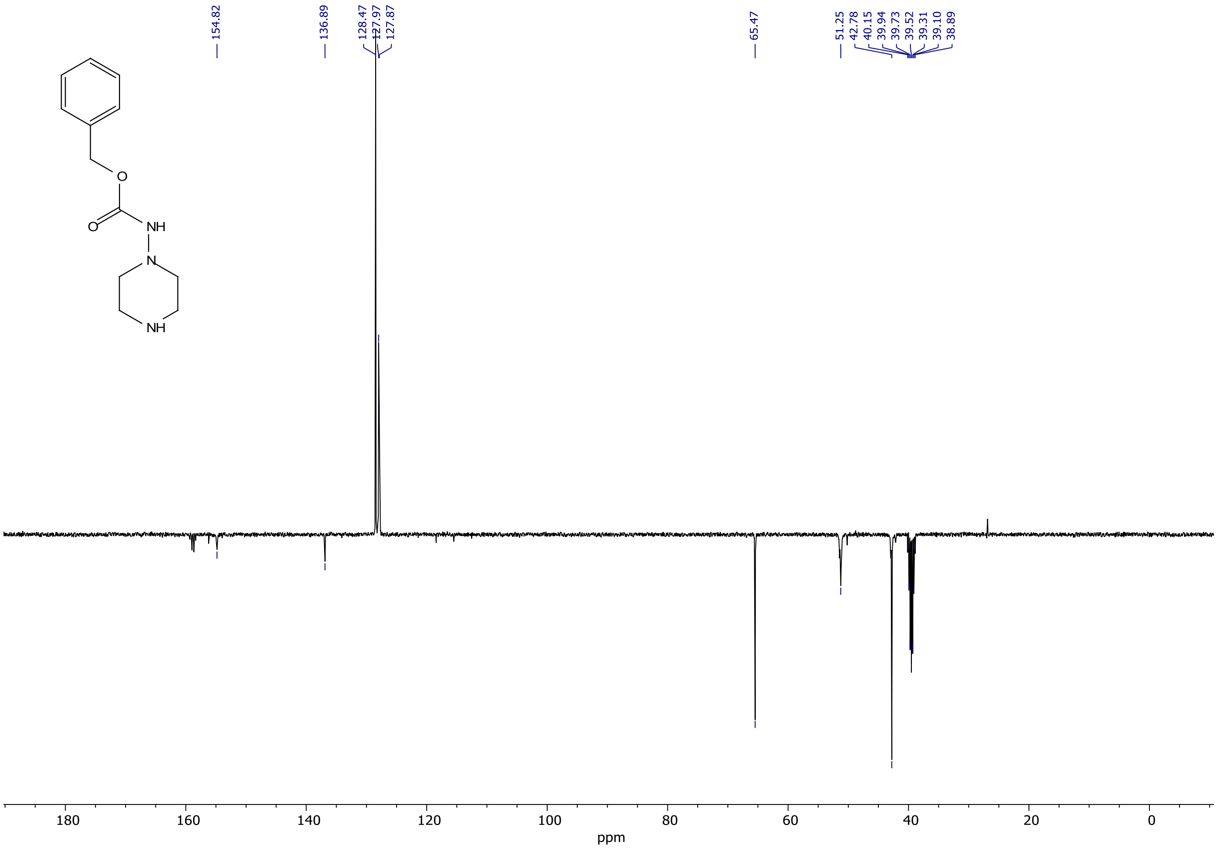

Figure S11. $^{13}C\{^{1}H\}$ NMR (101 MHz, DMSO-d_6) of the compound **17**.

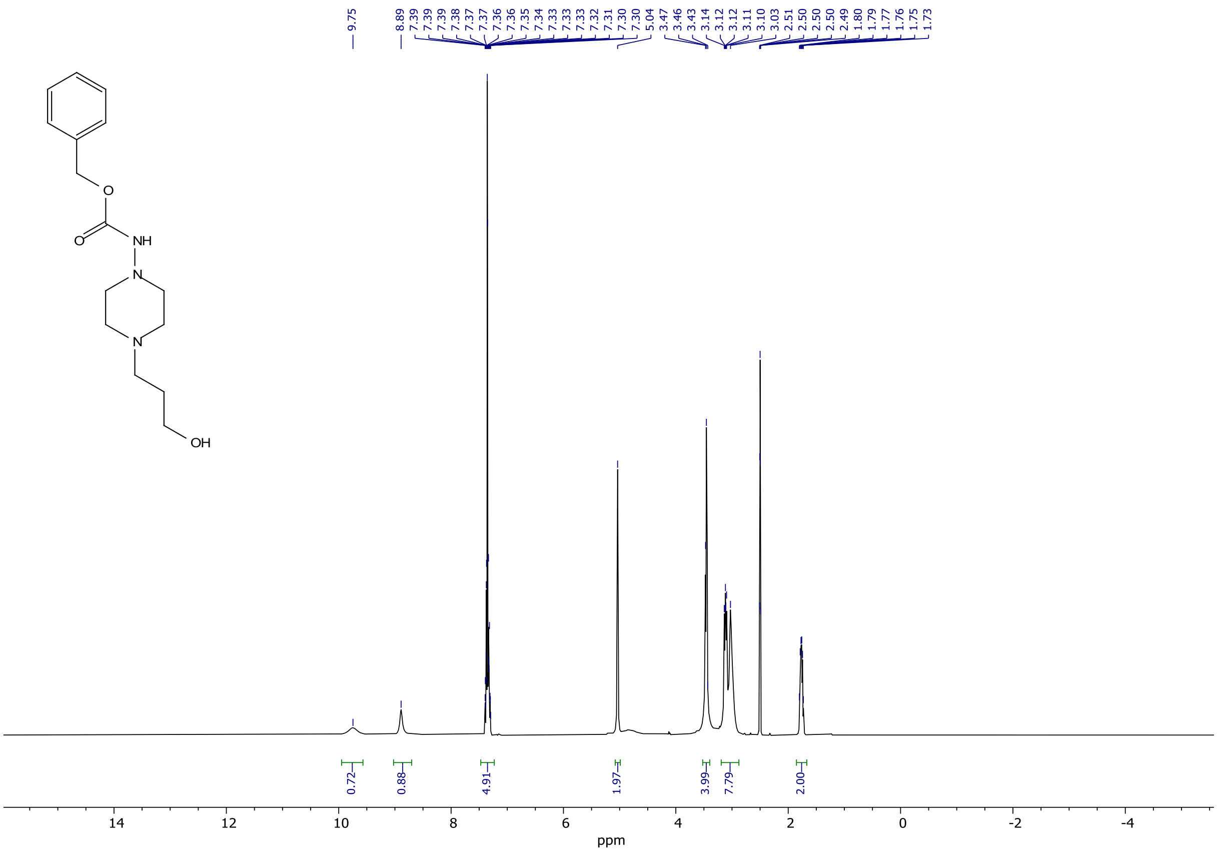

Figure S12. ^{1}H-NMR (400 MHz, DMSO-d_6) of compound **18**.

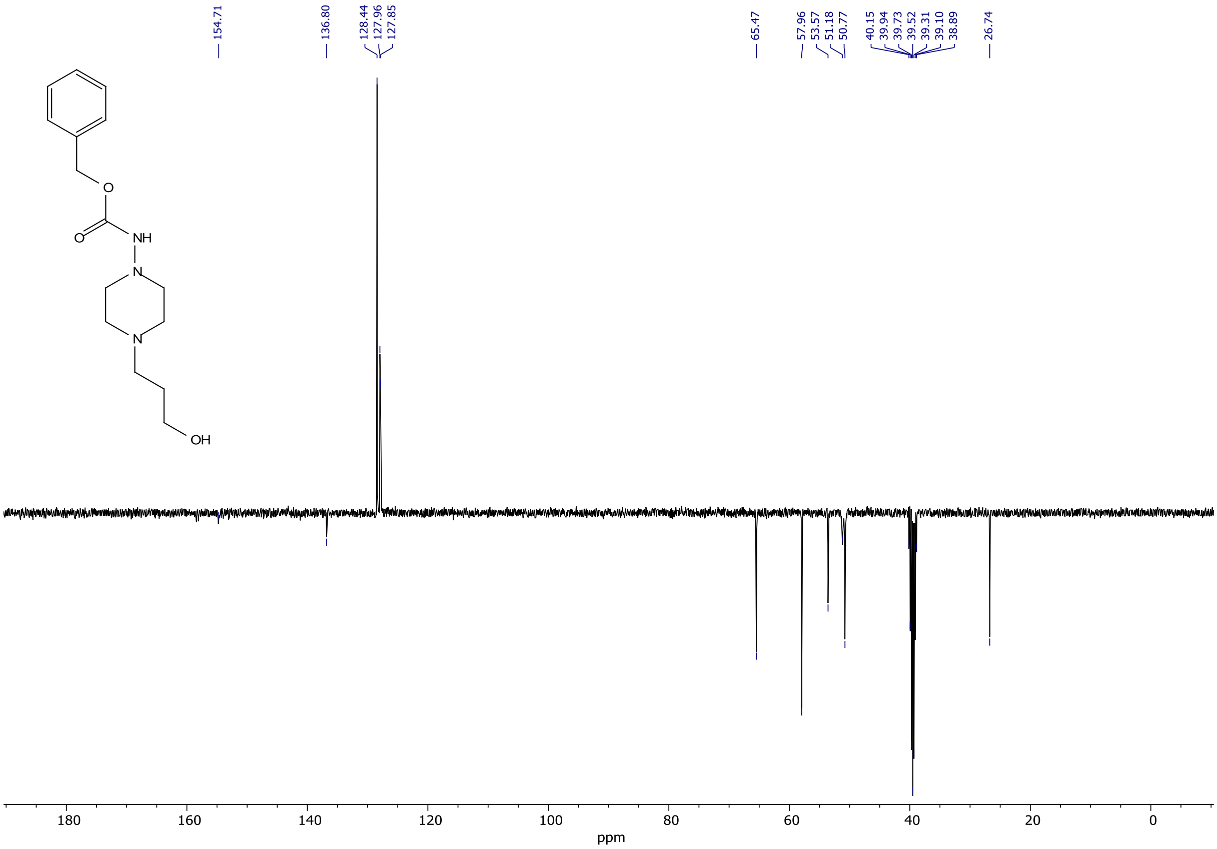

Figure S13. ^{13}C{^{1}H} NMR (101 MHz, DMSO-d_6) of the compound **18**.

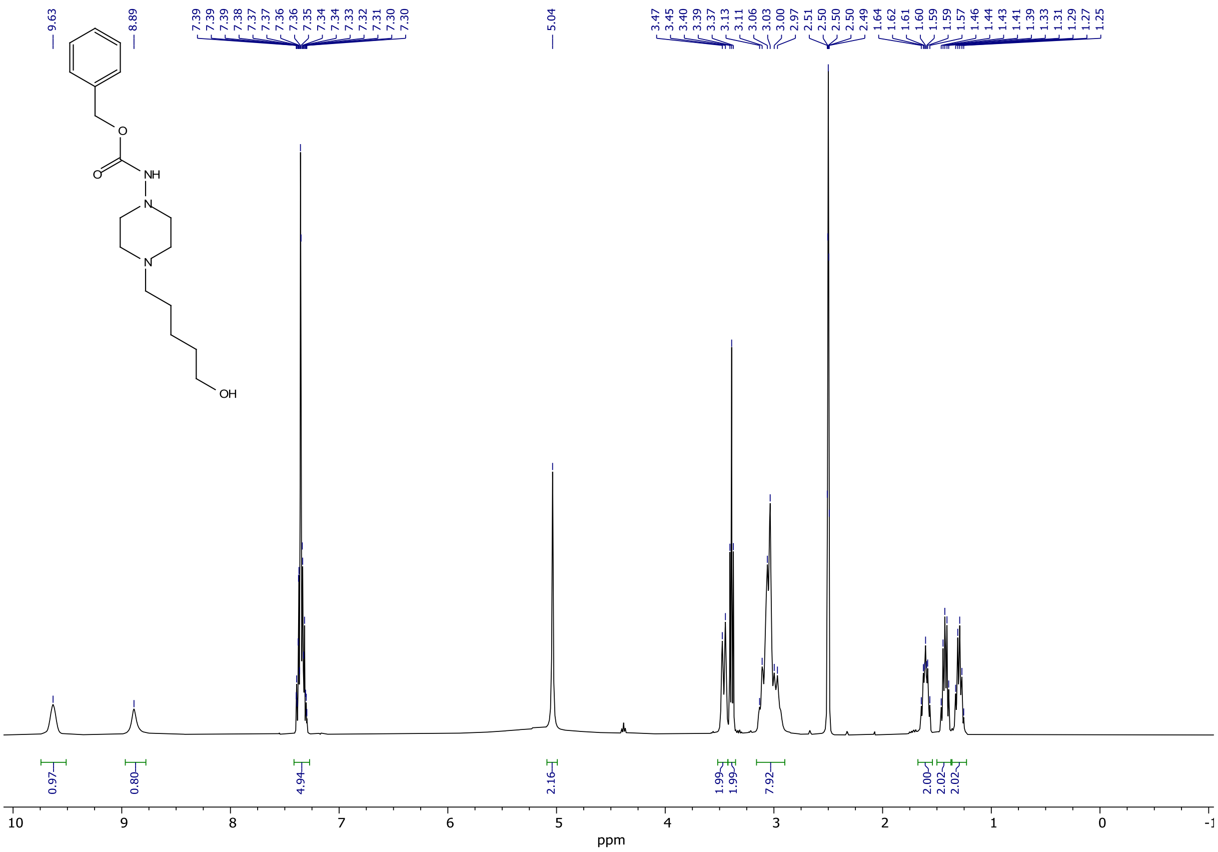

Figure S14. ^{1}H-NMR (400 MHz, DMSO-d_6) of compound **19**.

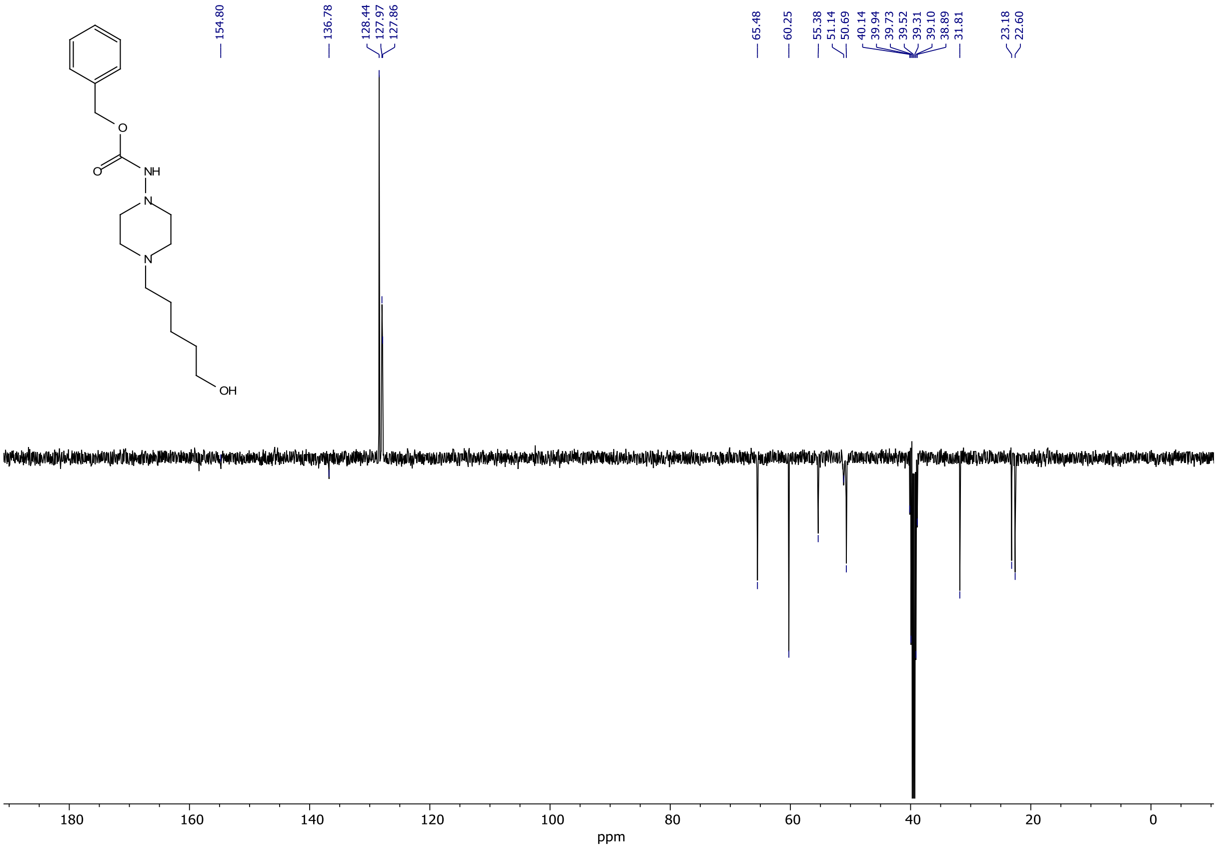

Figure S15. ^{13}C{^{1}H} NMR (101 MHz, DMSO-d_6) of the compound **19**.

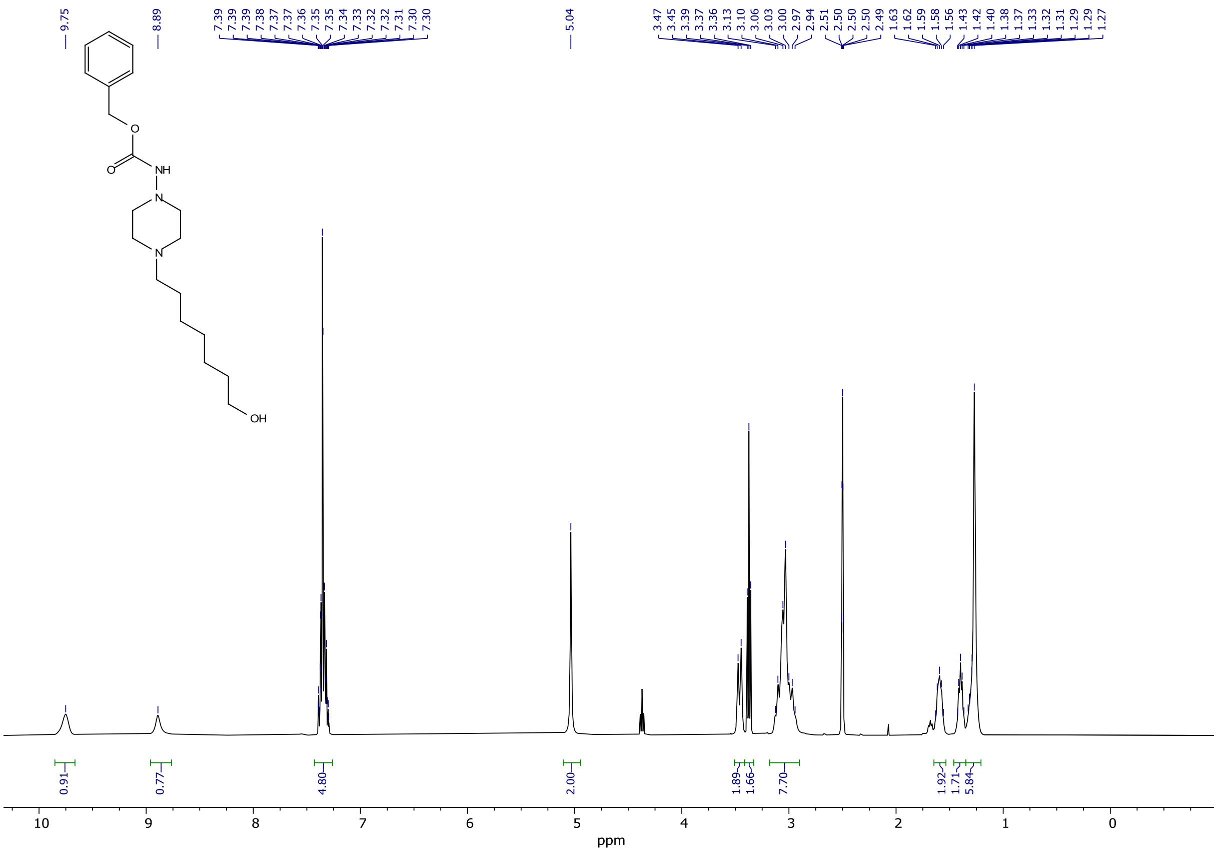

Figure S16. ^{1}H-NMR (400 MHz, DMSO-d_6) of compound **20**.

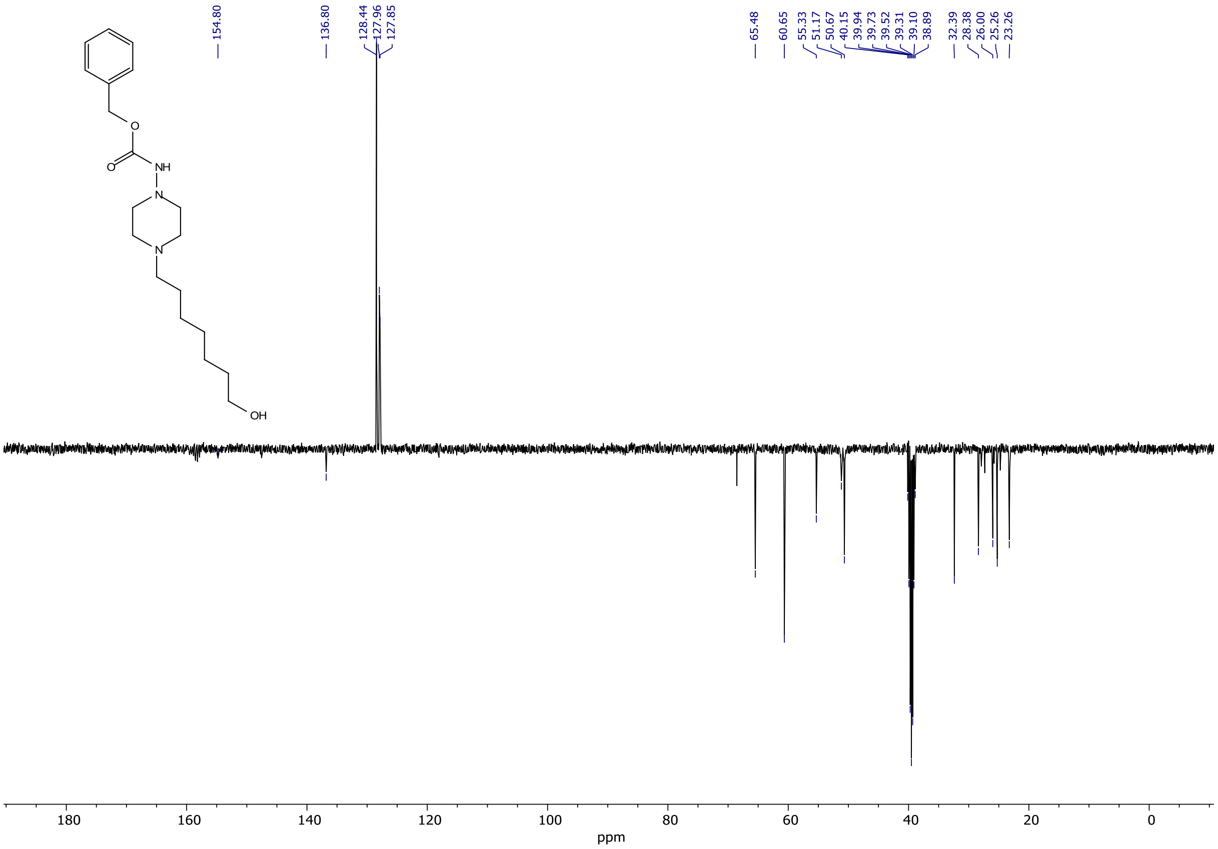

Figure S17. $^{13}C\{^{1}H\}$ NMR (101 MHz, DMSO-d_6) of the compound **20**.

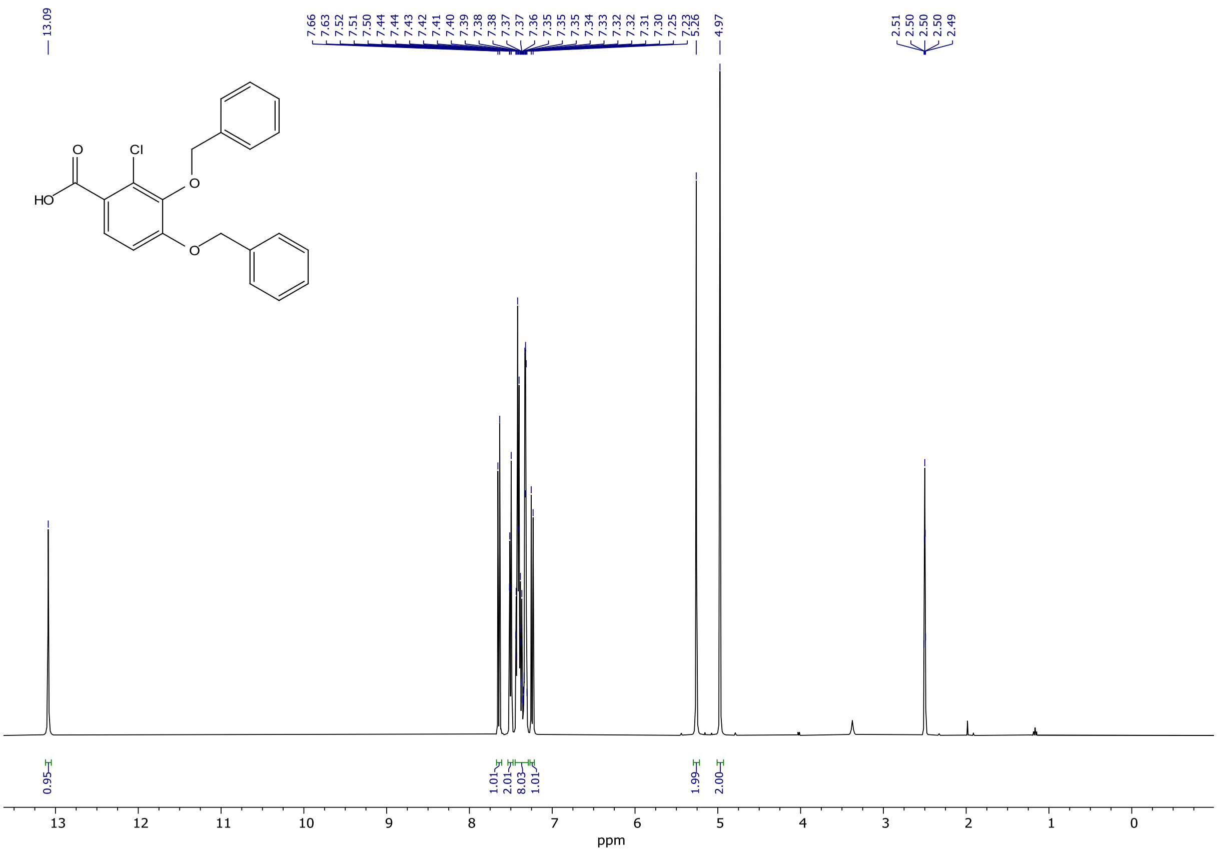

Figure S18. ^{1}H-NMR (400 MHz, DMSO-d_6) of compound **25**.

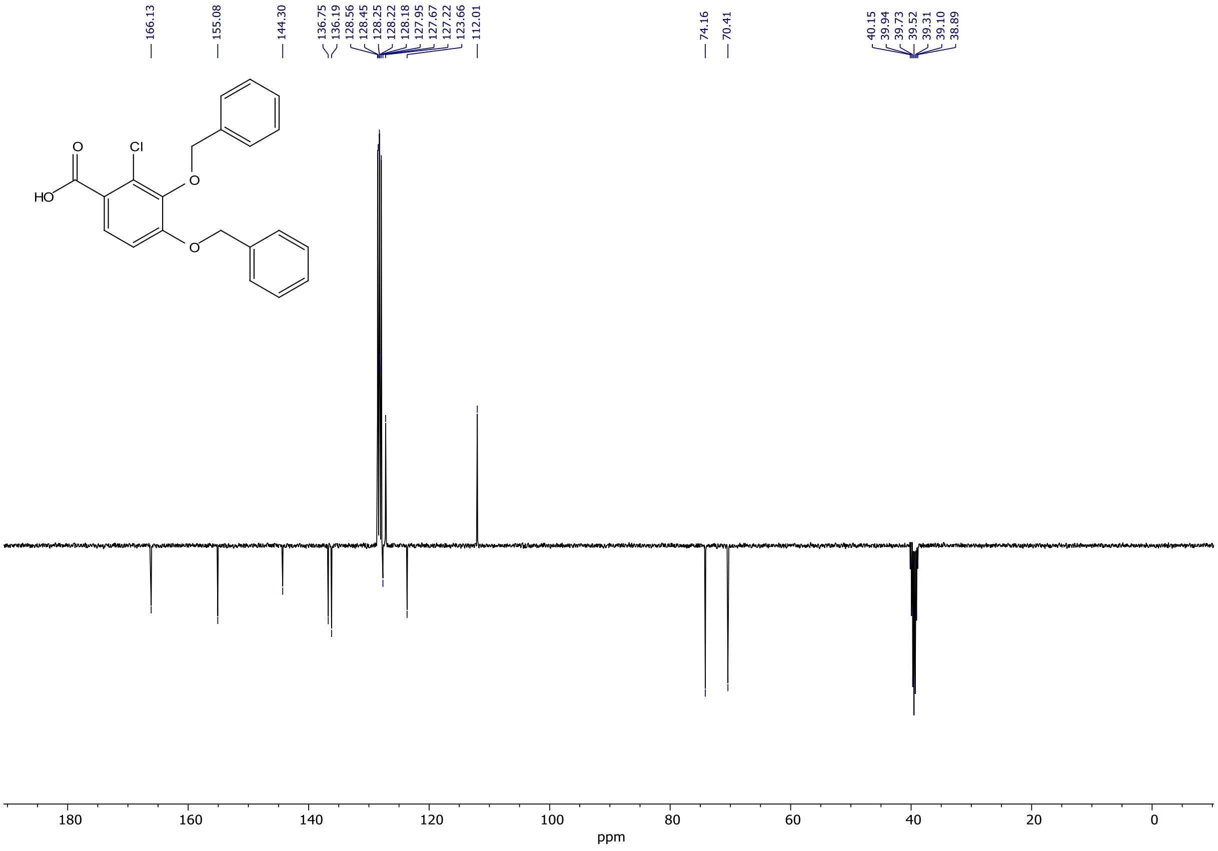

Figure S19. ^{13}C{^{1}H} NMR (101 MHz, DMSO-d_6) of the compound **25**.

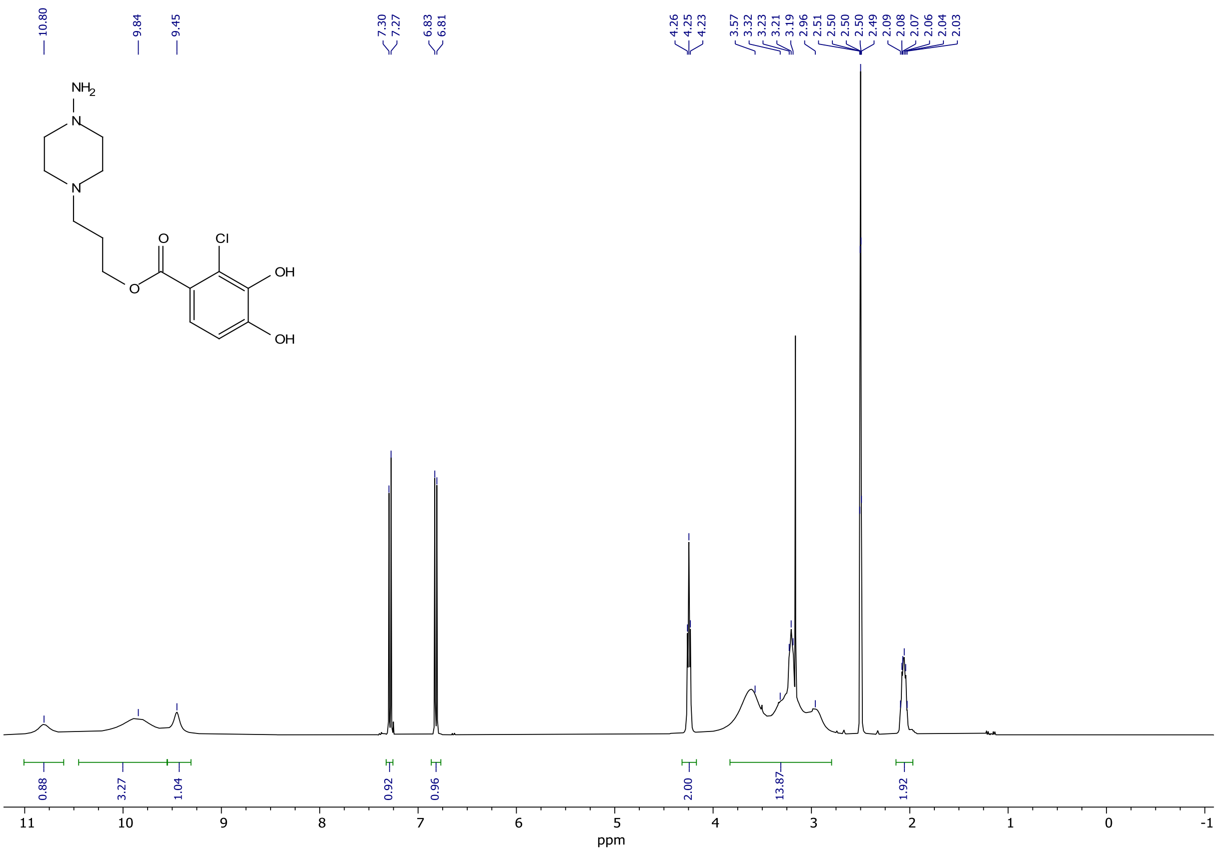

Figure S20. ^{1}H-NMR (400 MHz, DMSO-d_6) of compound **27**.

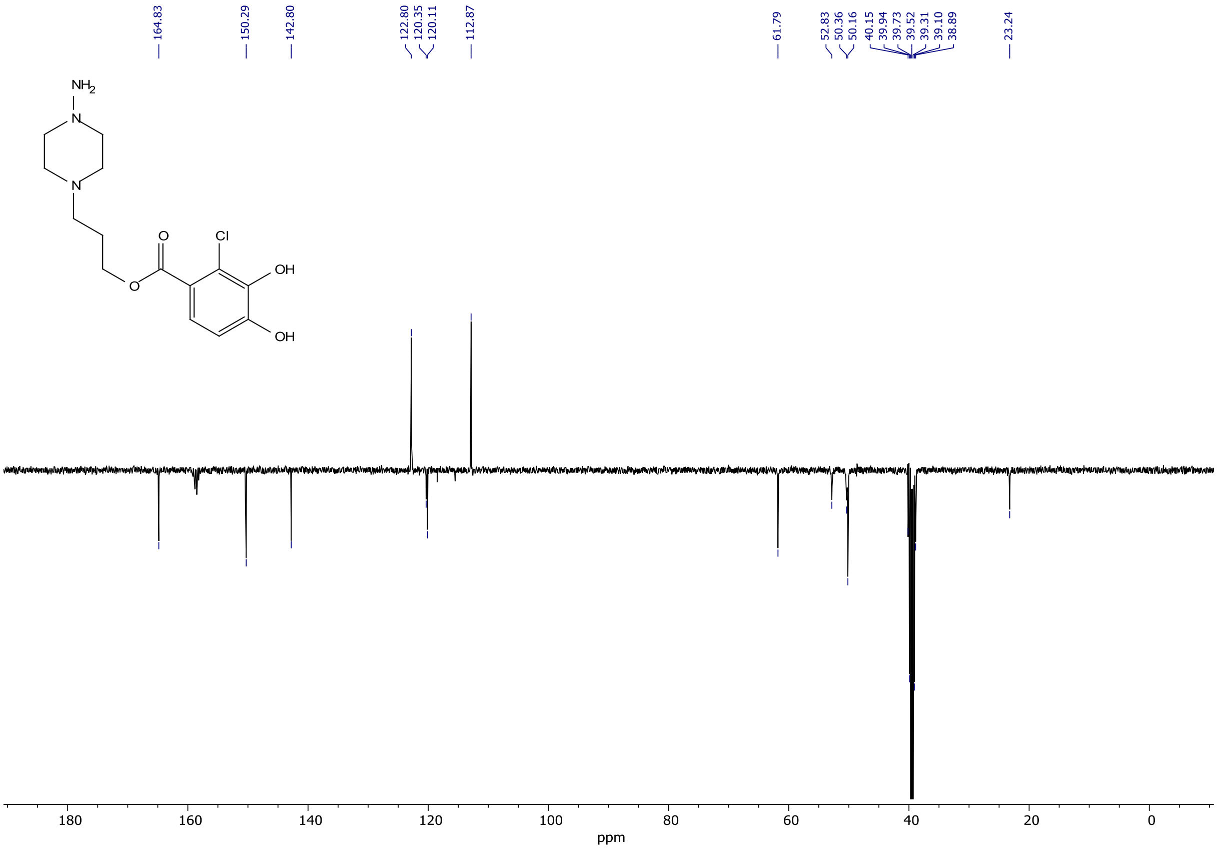

Figure S21. ^{13}C{^{1}H} NMR (101 MHz, DMSO-d_6) of the compound **27**.

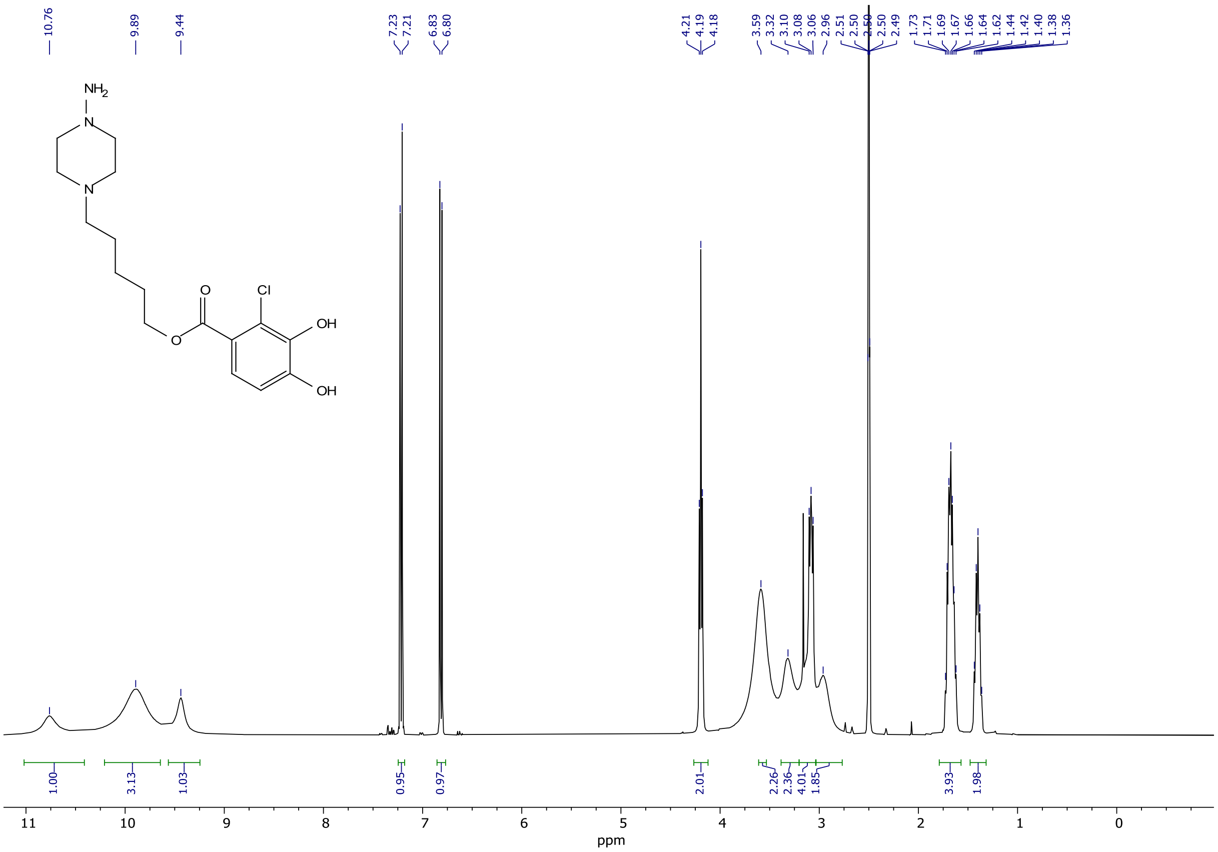

Figure S22. ^{1}H-NMR (400 MHz, DMSO-d_6) of compound **28**.

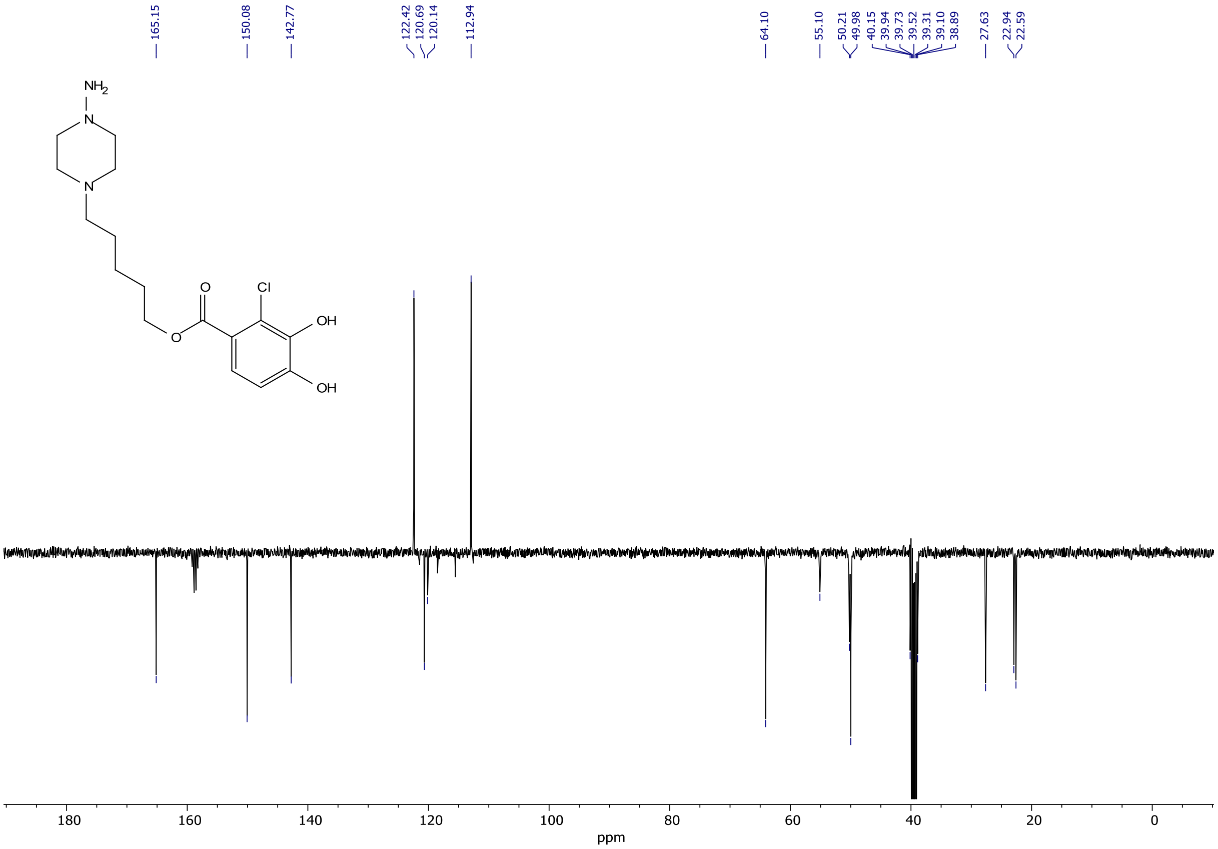

Figure S23. $^{13}C\{^{1}H\}$ NMR (101 MHz, DMSO-d_6) of the compound **28**.

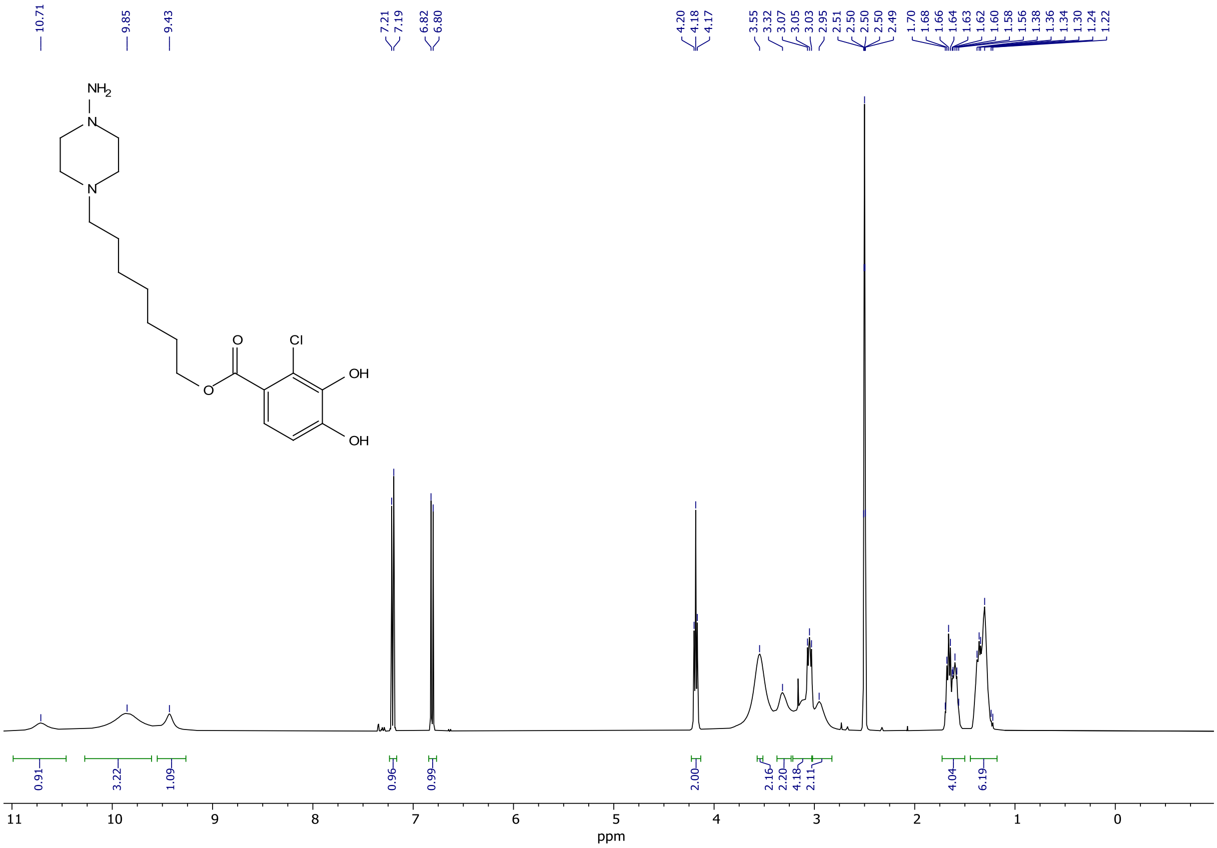

Figure S24. ^{1}H-NMR (400 MHz, DMSO-*d*$_{6}$) of compound **29**.

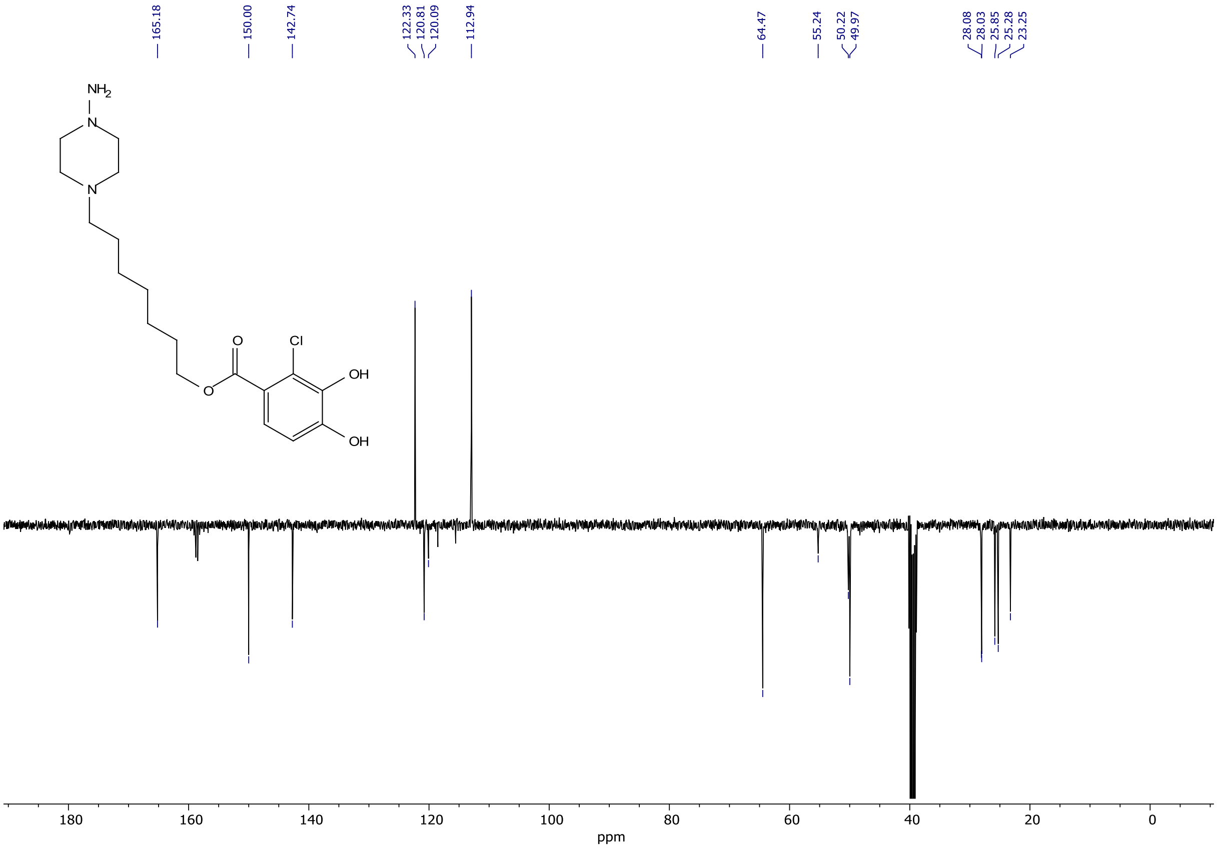

Figure S25. ^{13}C{^{1}H} NMR (101 MHz, DMSO-*d*$_{6}$) of the compound **29**.

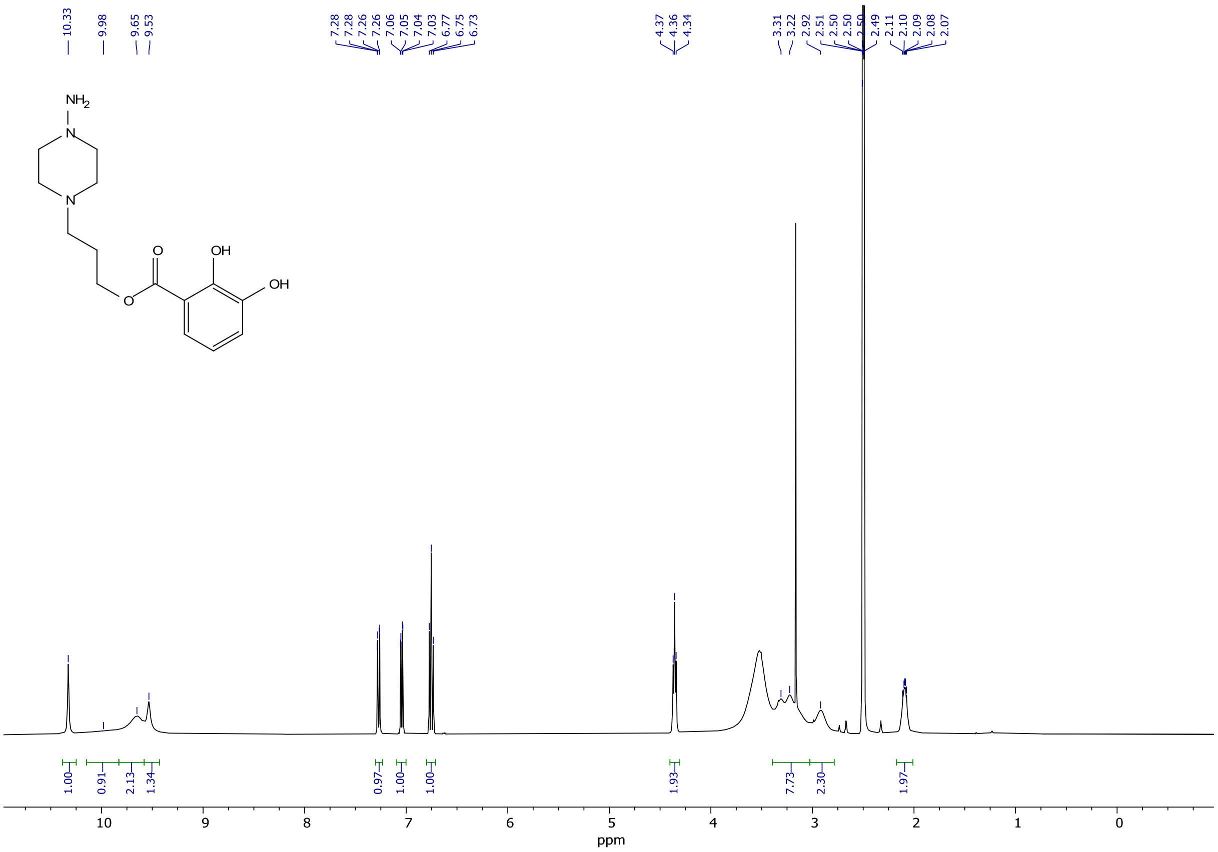

Figure S26. ^{1}H-NMR (400 MHz, DMSO-d_6) of compound **30**.

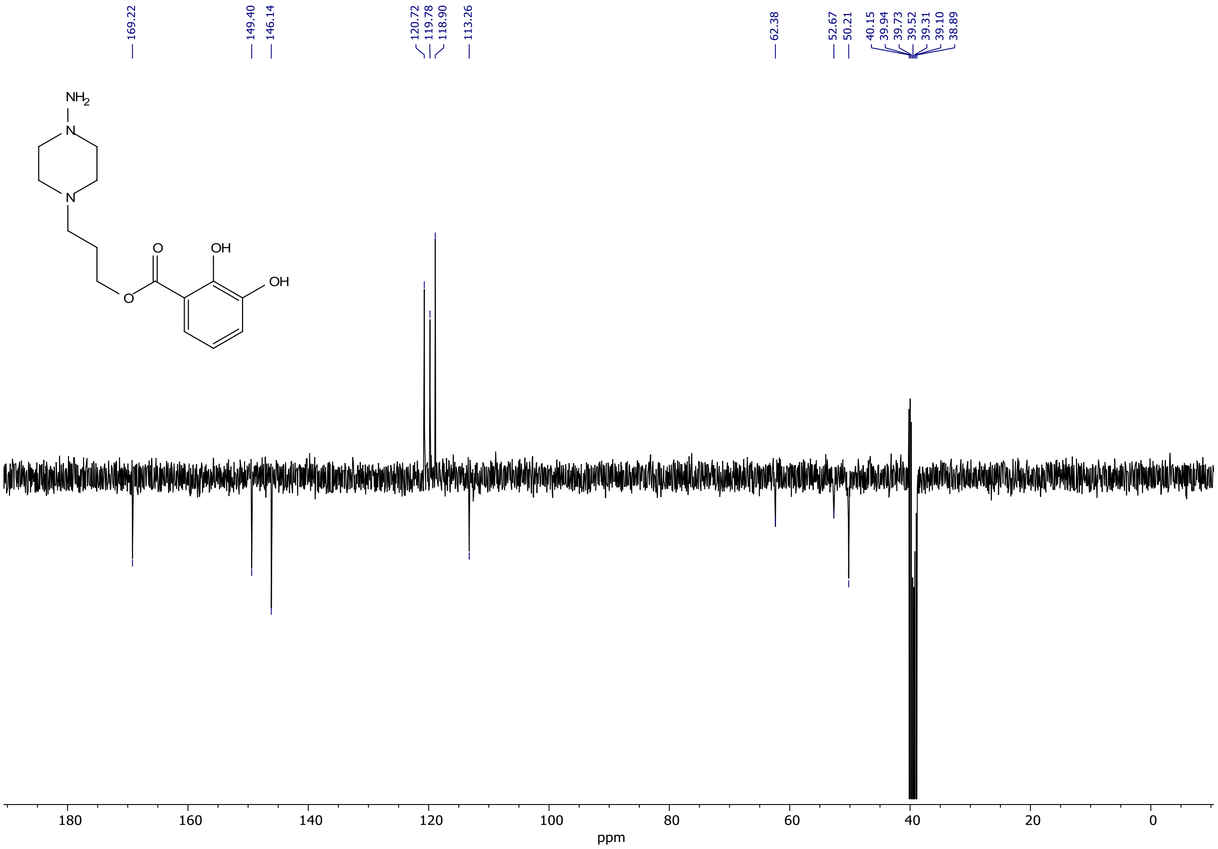

Figure S27. $^{13}C\{^{1}H\}$ NMR (101 MHz, DMSO-d_6) of the compound **30**.

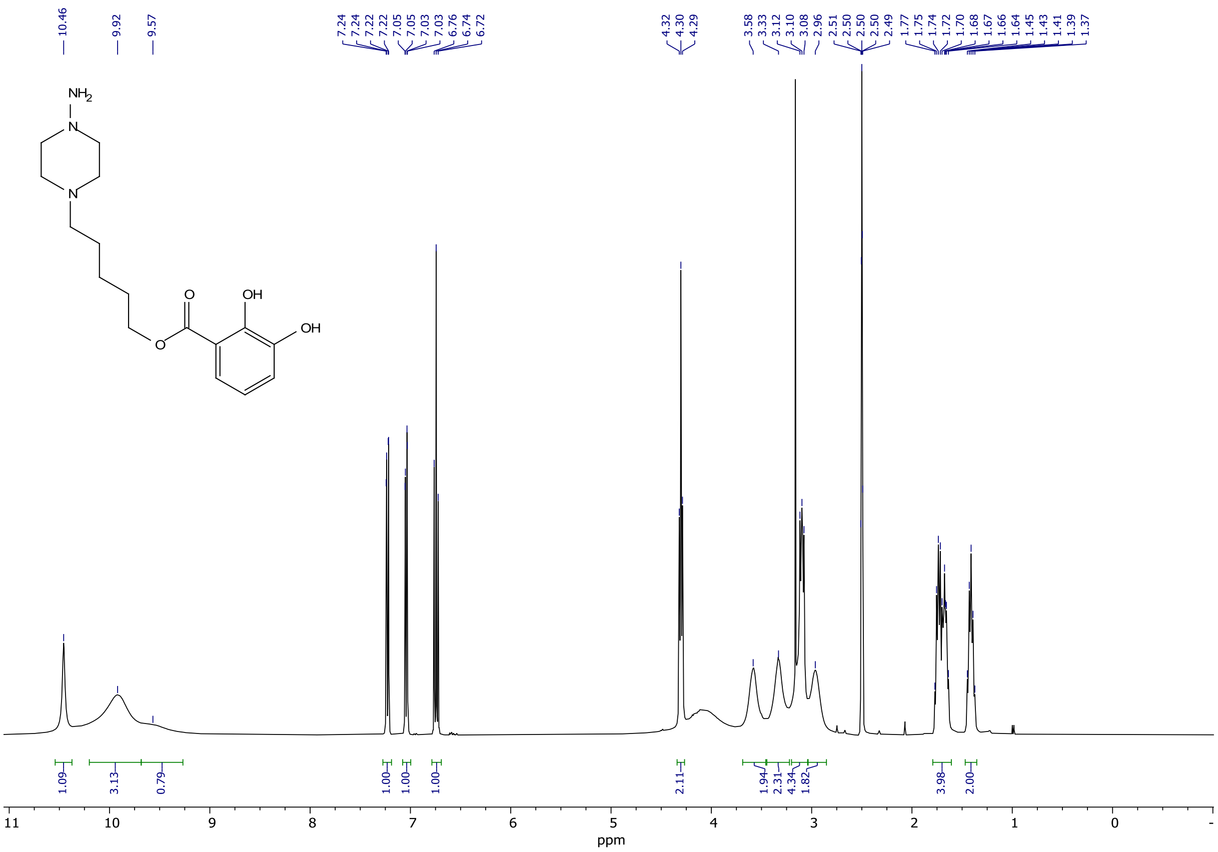

Figure S28. ^{1}H-NMR (400 MHz, DMSO-d_6) of compound **31**.

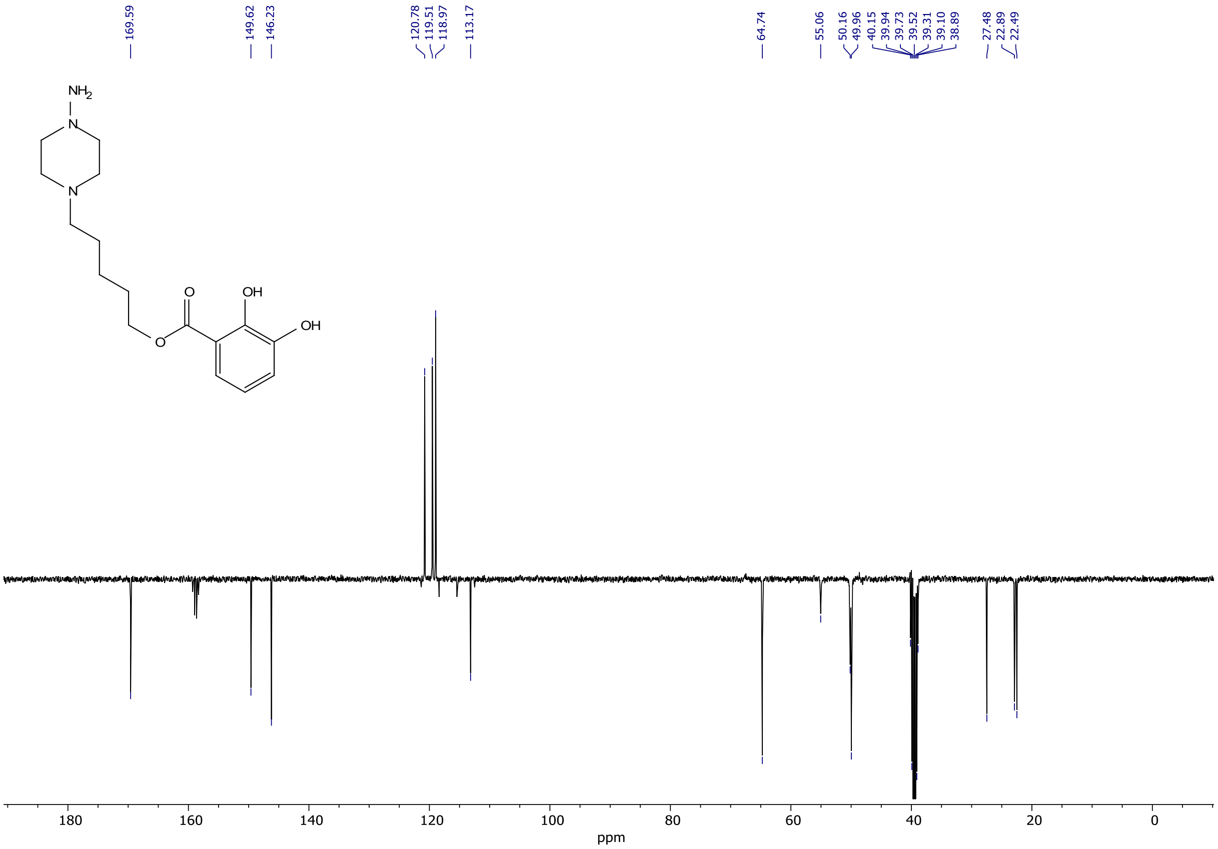

Figure S29. $^{13}C\{^{1}H\}$ NMR (101 MHz, DMSO-d_6) of the compound **31**.

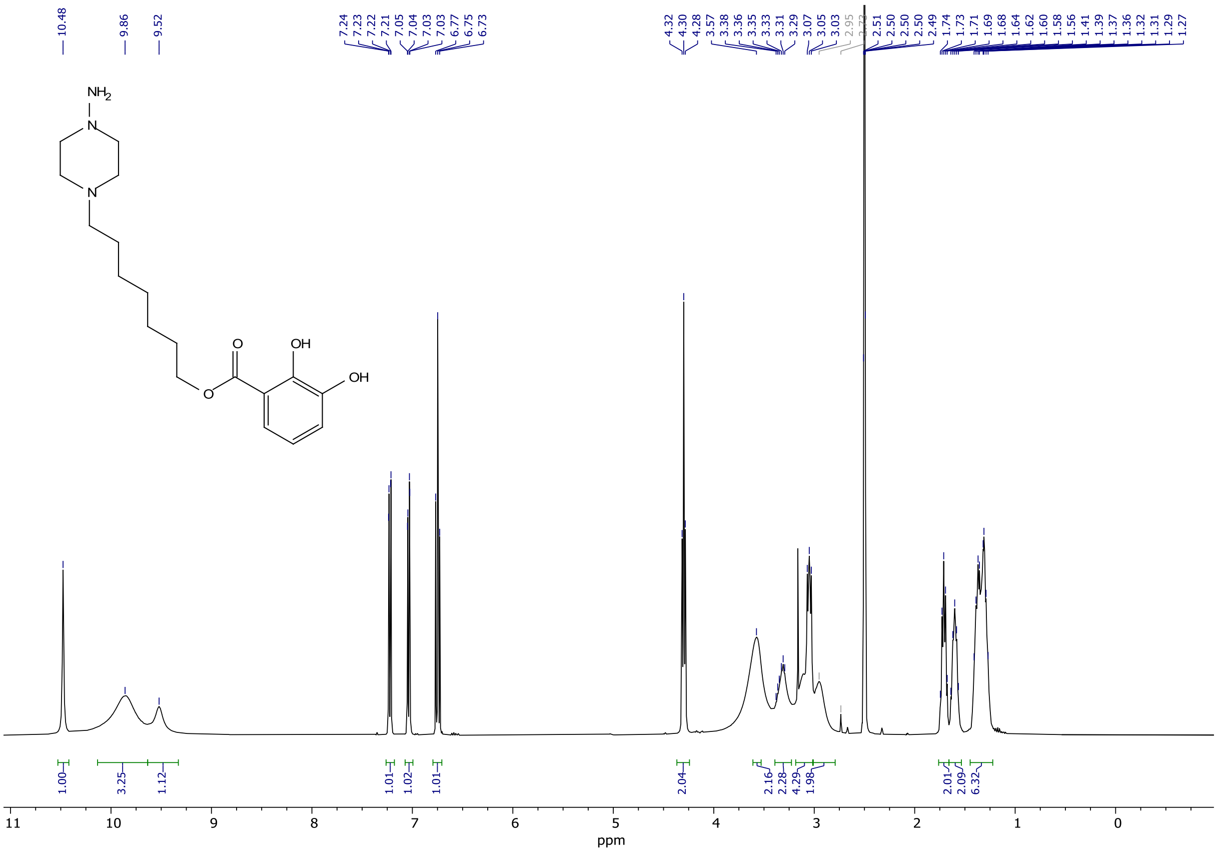

Figure S30. ^{1}H-NMR (400 MHz, DMSO-d_6) of compound **32**.

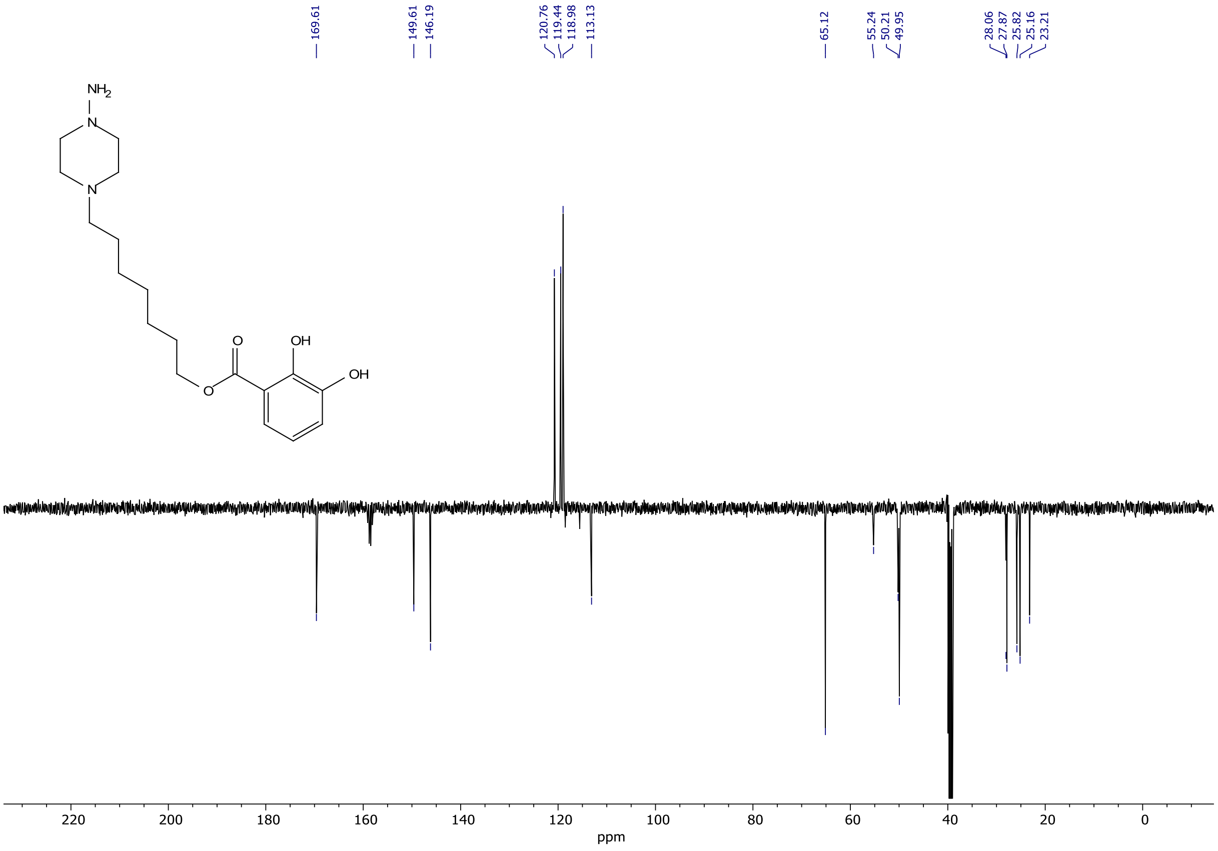

Figure S31. ^{13}C{^{1}H} NMR (101 MHz, DMSO-d_6) of the compound **32**.

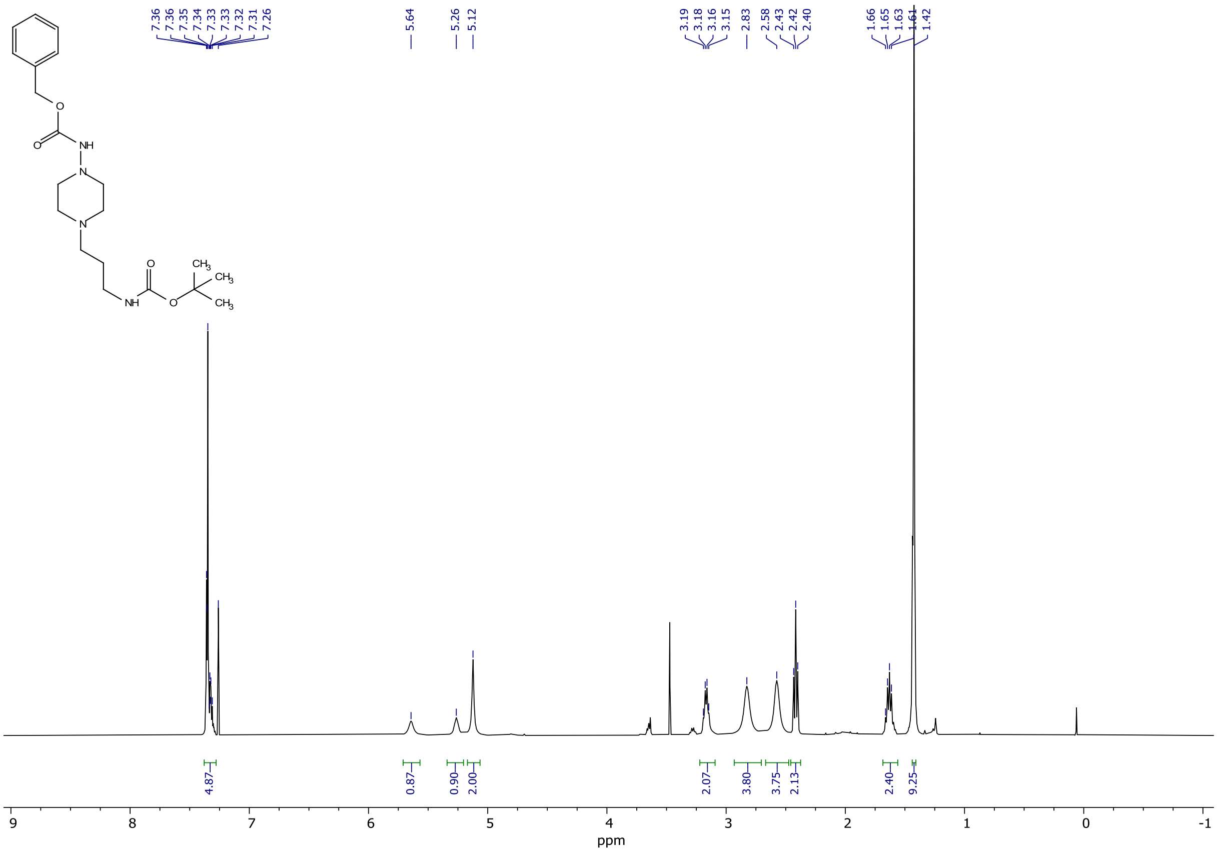

Figure S32. ^{1}H-NMR (400 MHz, $CDCl_3$) of compound **S9**.

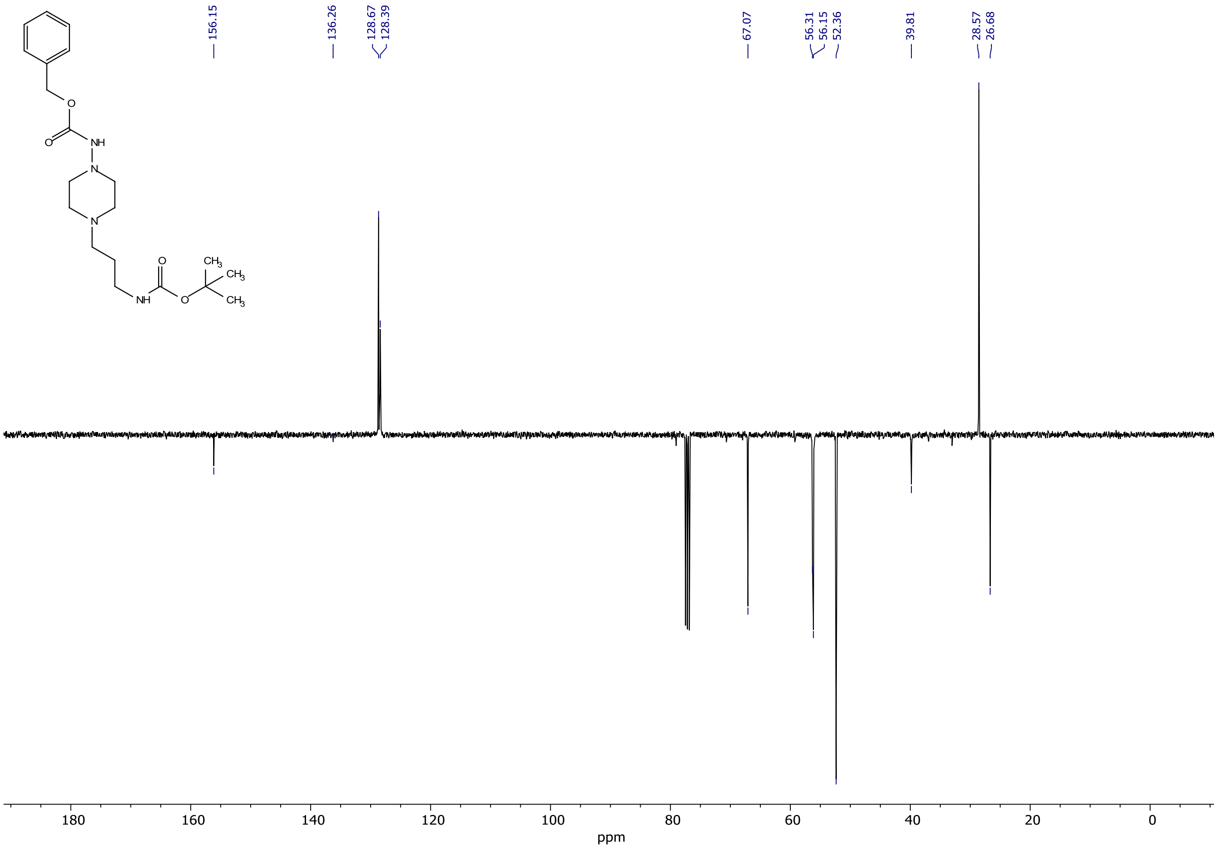

Figure S33. $^{13}C\{^1H\}$ NMR (101 MHz, $CDCl_3$) of the compound **S9**.

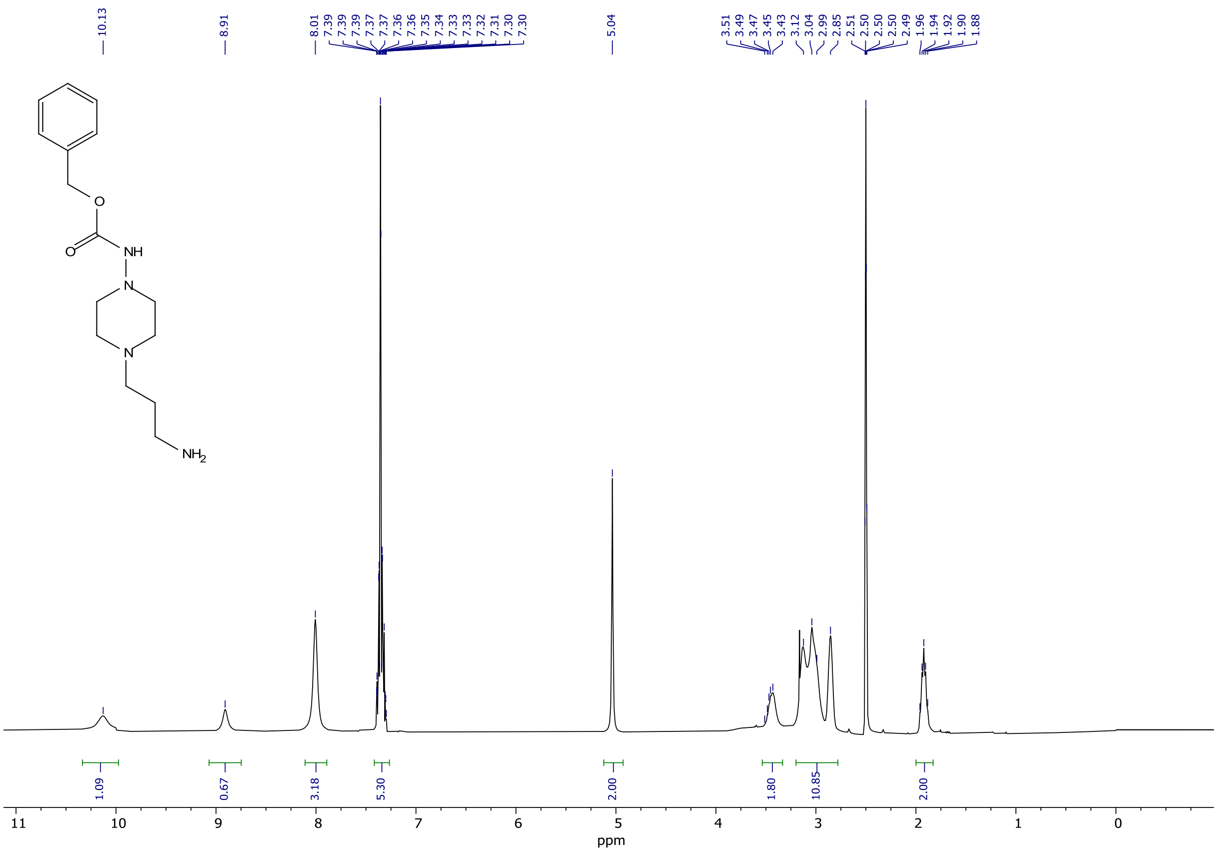

Figure S34. ^{1}H-NMR (400 MHz, DMSO-d_6) of compound **S10**.

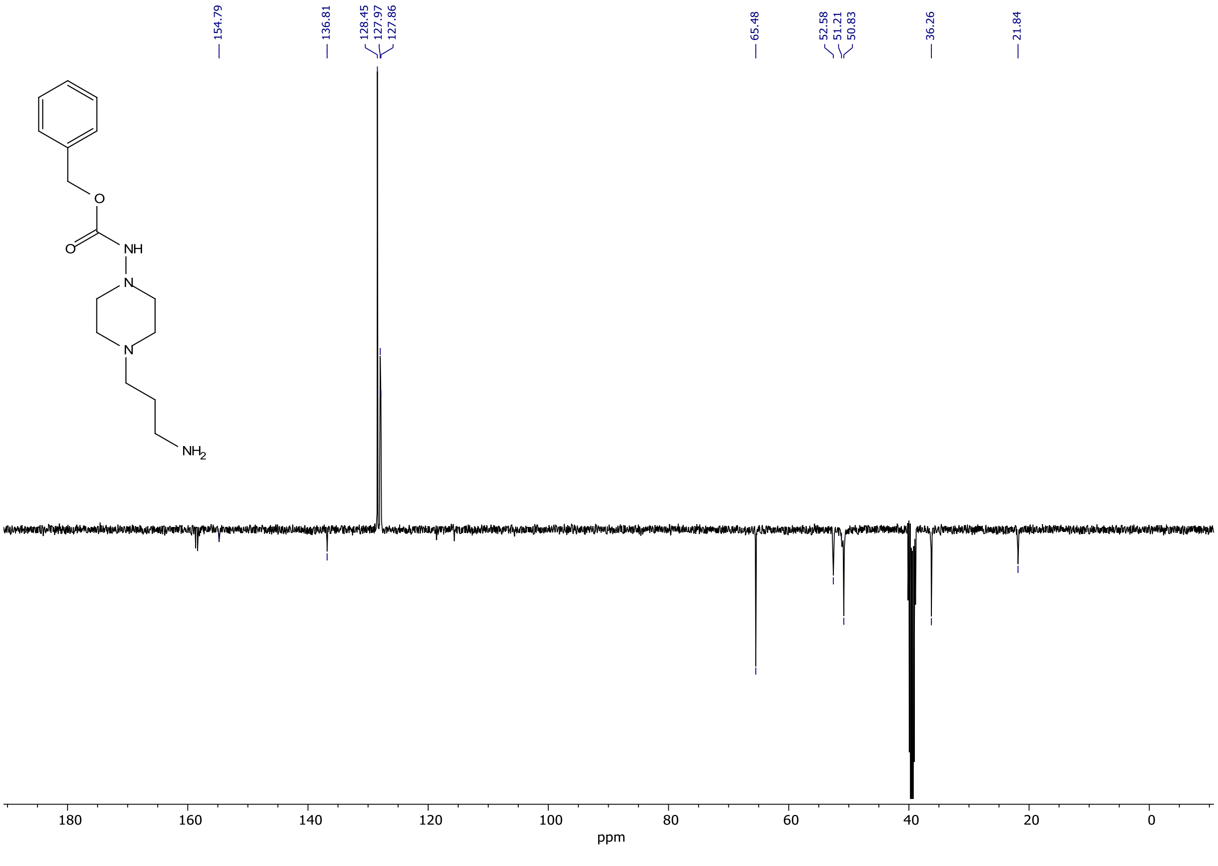

Figure S35. $^{13}C\{^{1}H\}$ NMR (101 MHz, DMSO-d_6) of the compound **S10**.

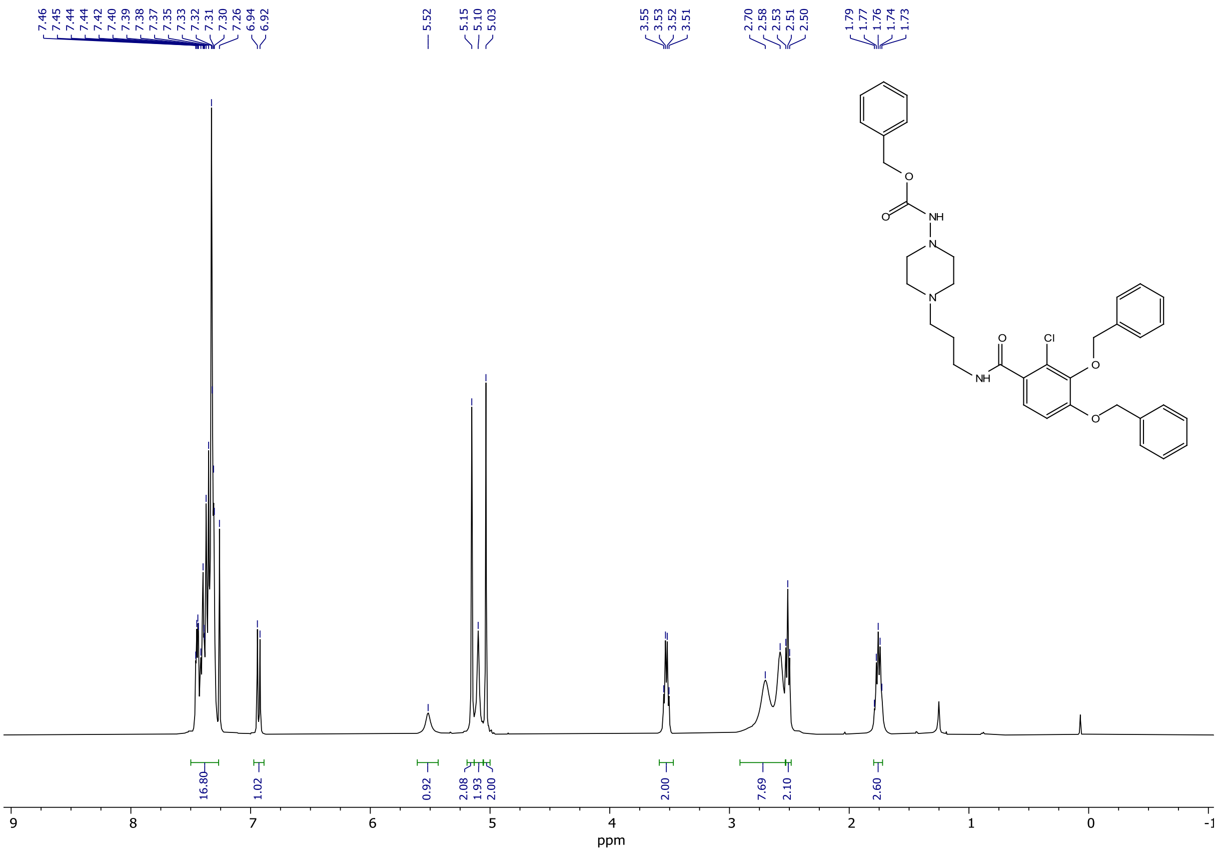

Figure S36. ^{1}H-NMR (400 MHz, $CDCl_3$) of compound **S11**.

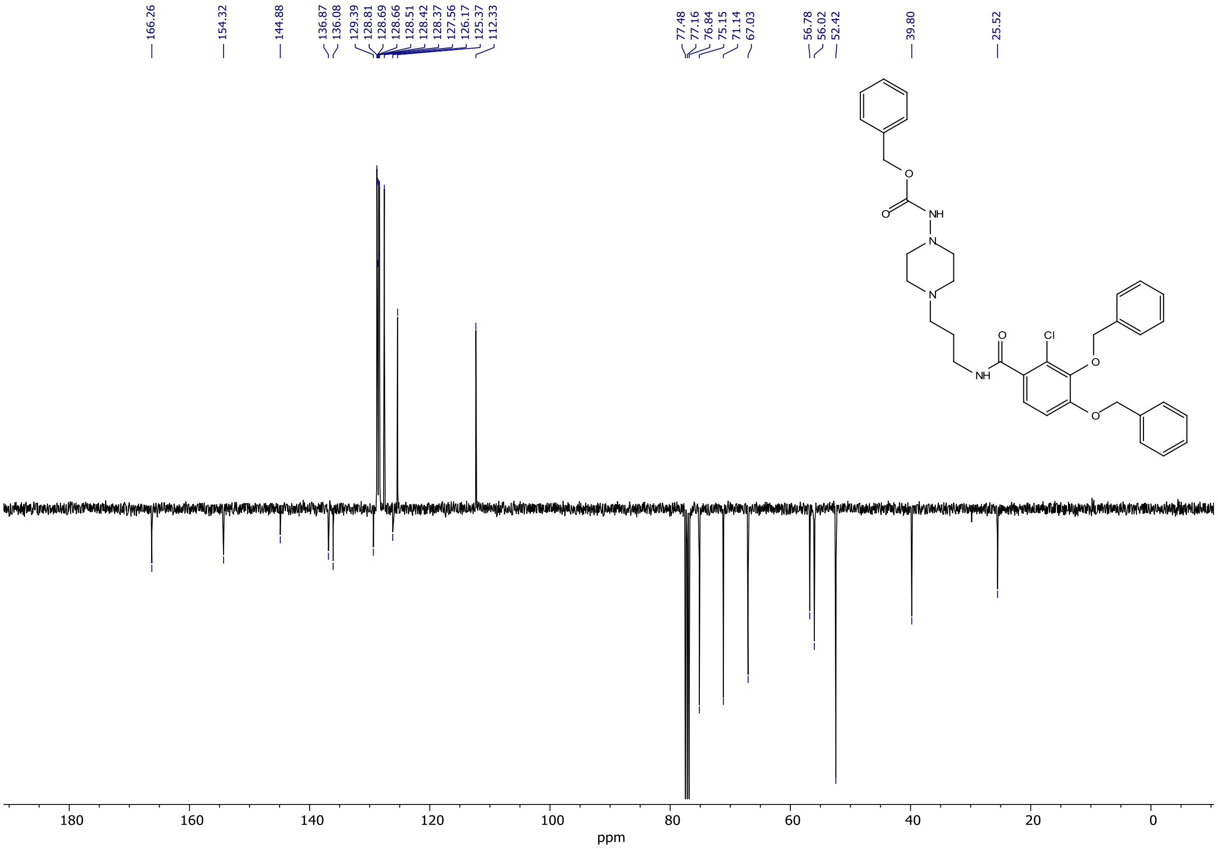

Figure S37. $^{13}C\{^{1}H\}$ NMR (101 MHz, $CDCl_3$) of the compound **S11**.

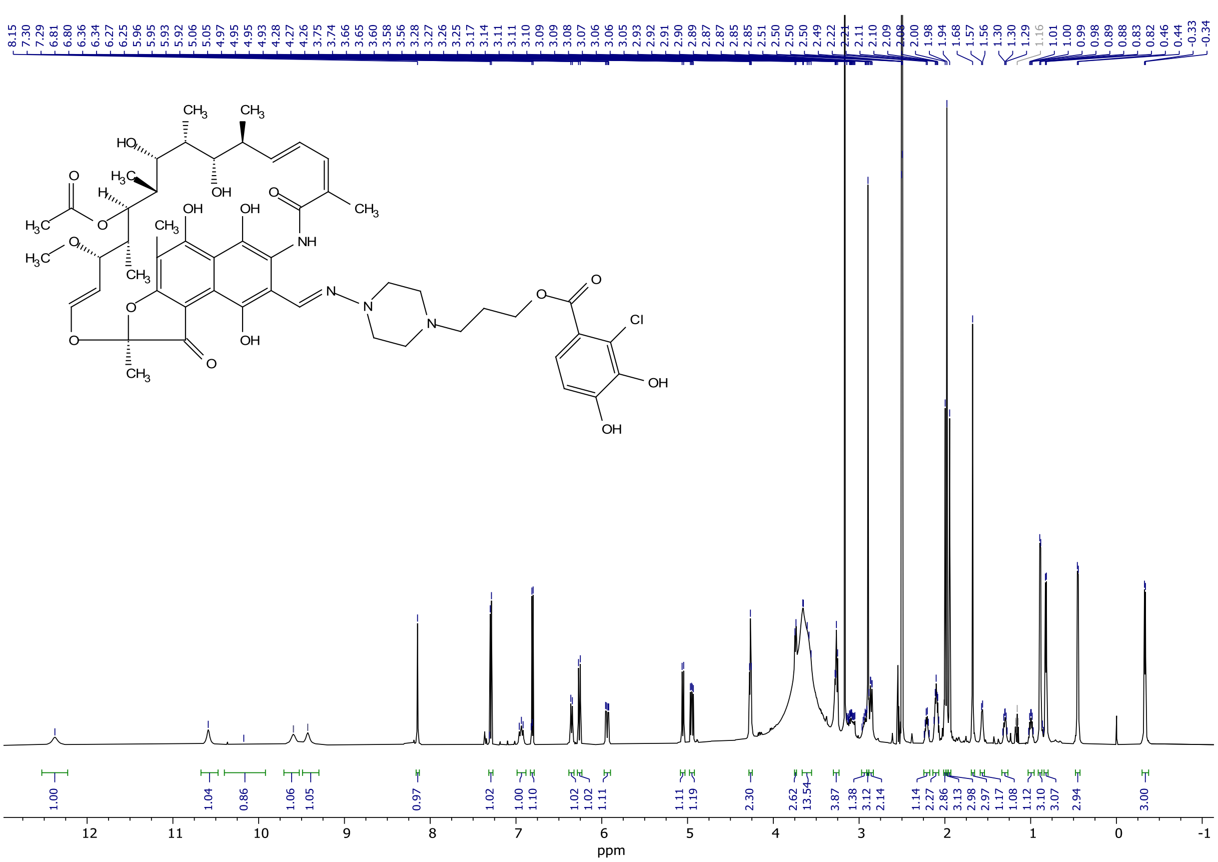

Figure S38. ^{1}H-NMR (400 MHz, DMSO-d_6) of compound **33**.

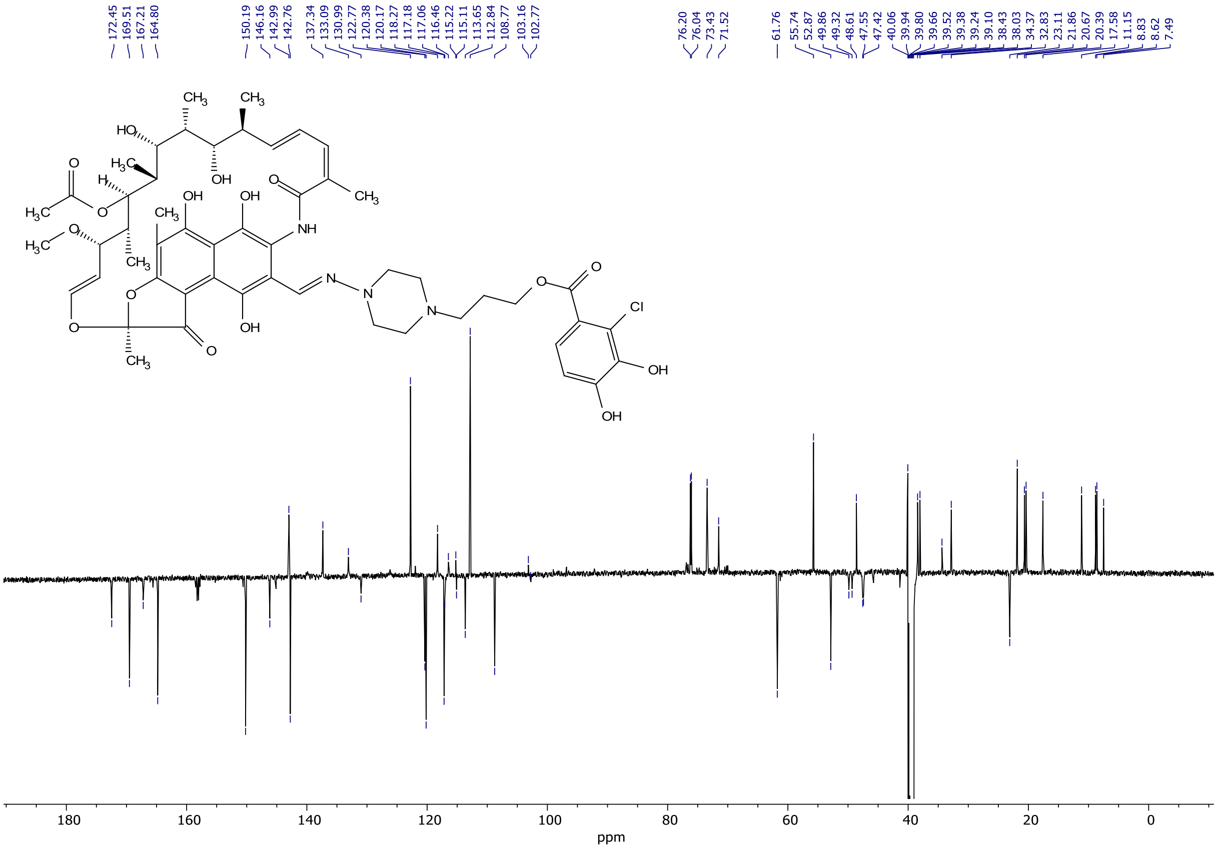

Figure S39. $^{13}C\{^{1}H\}$ NMR (101 MHz, DMSO-d_6) of the compound **33**.

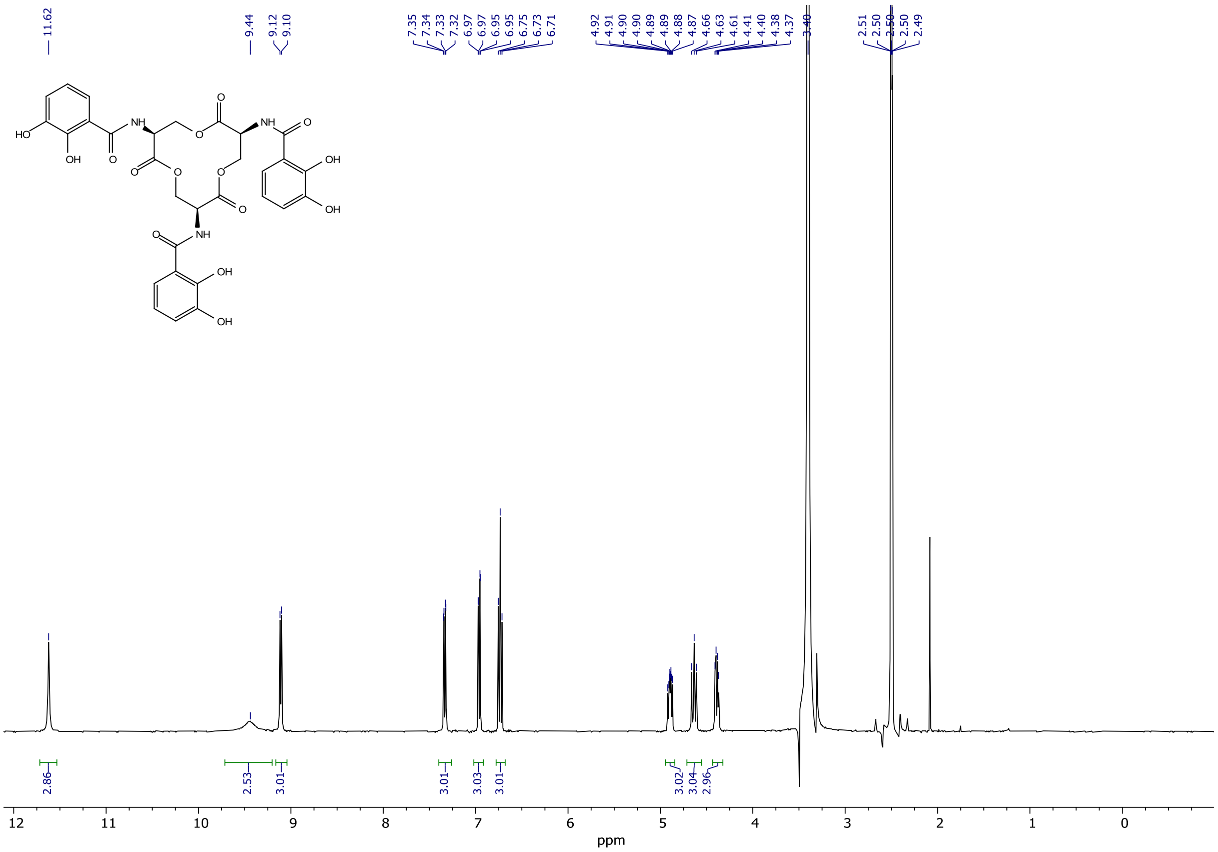

Figure S40. ^{1}H-NMR (400 MHz, DMSO-d_6) of enterobactin.

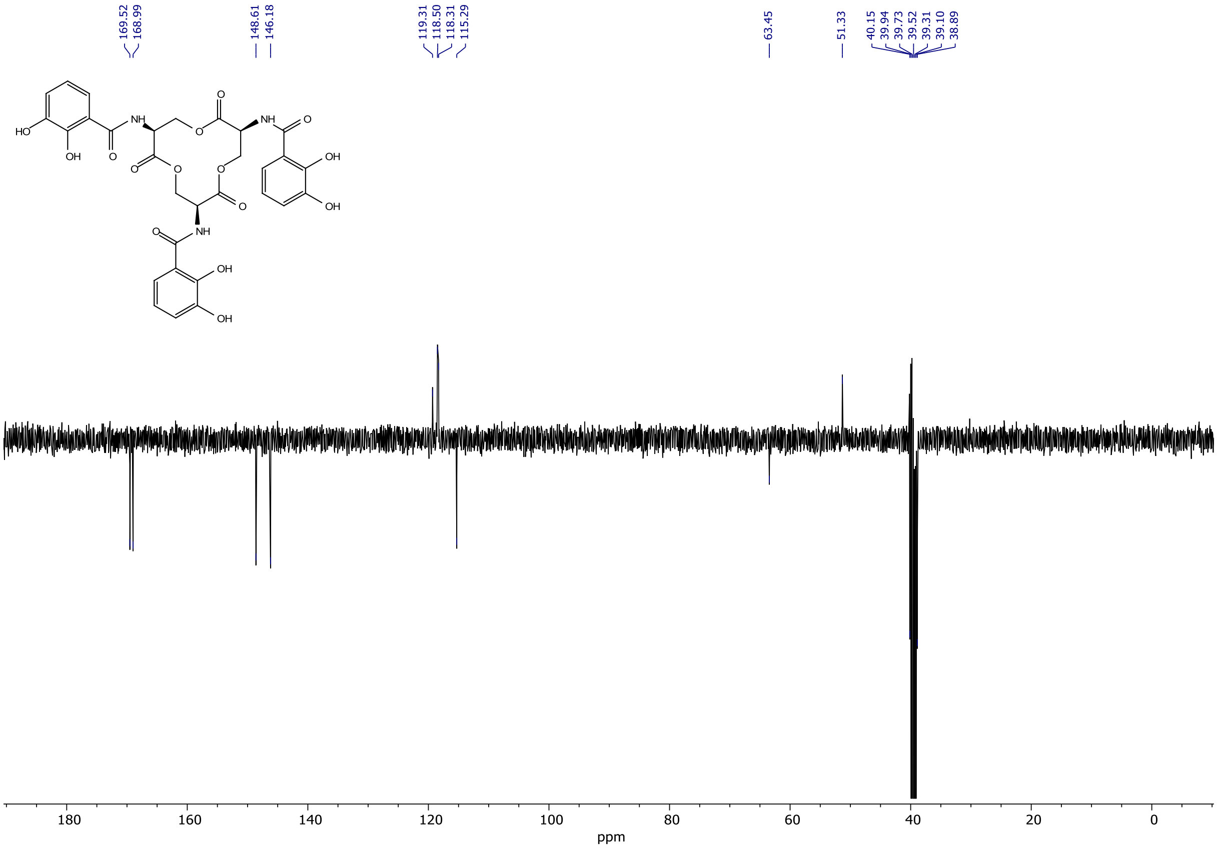

Figure S41. ^{13}C{^{1}H} NMR (101 MHz, DMSO-d_6) of the enterobactin.

HPLC traces

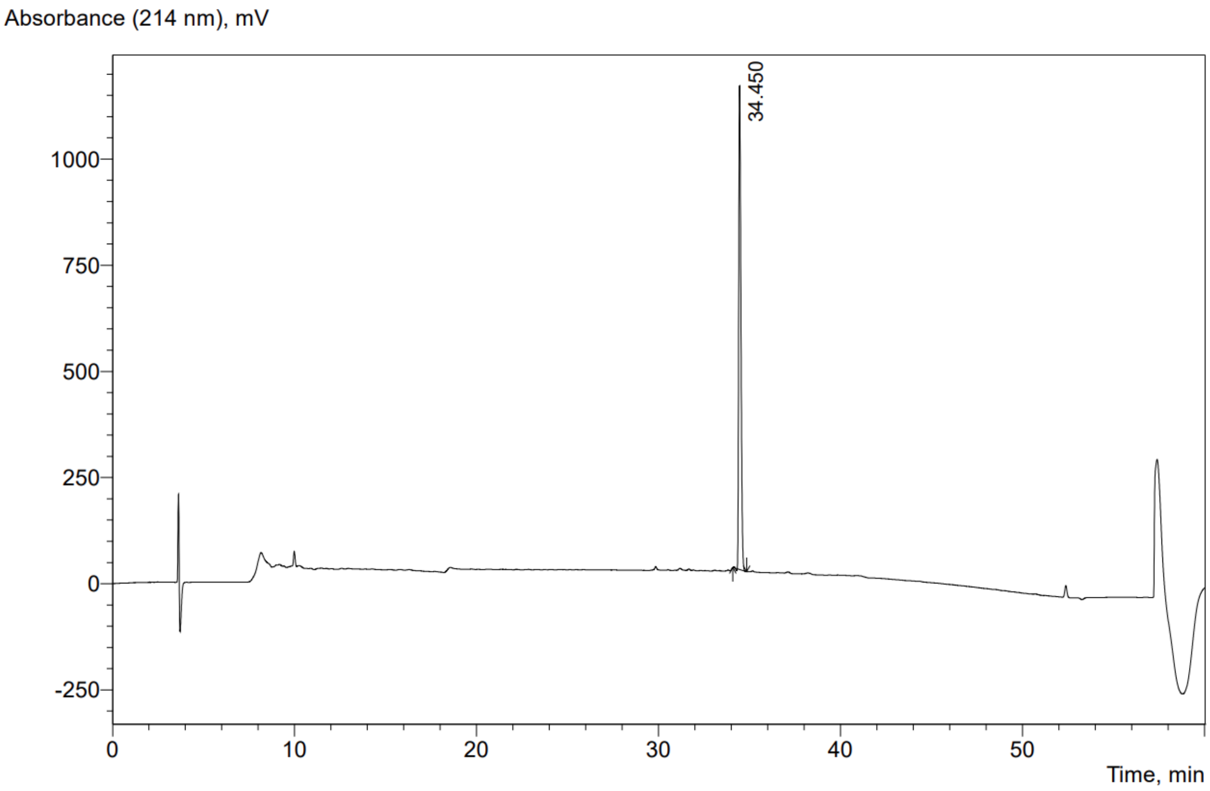

Figure S42. HPLC trace of the purified compound **1**.

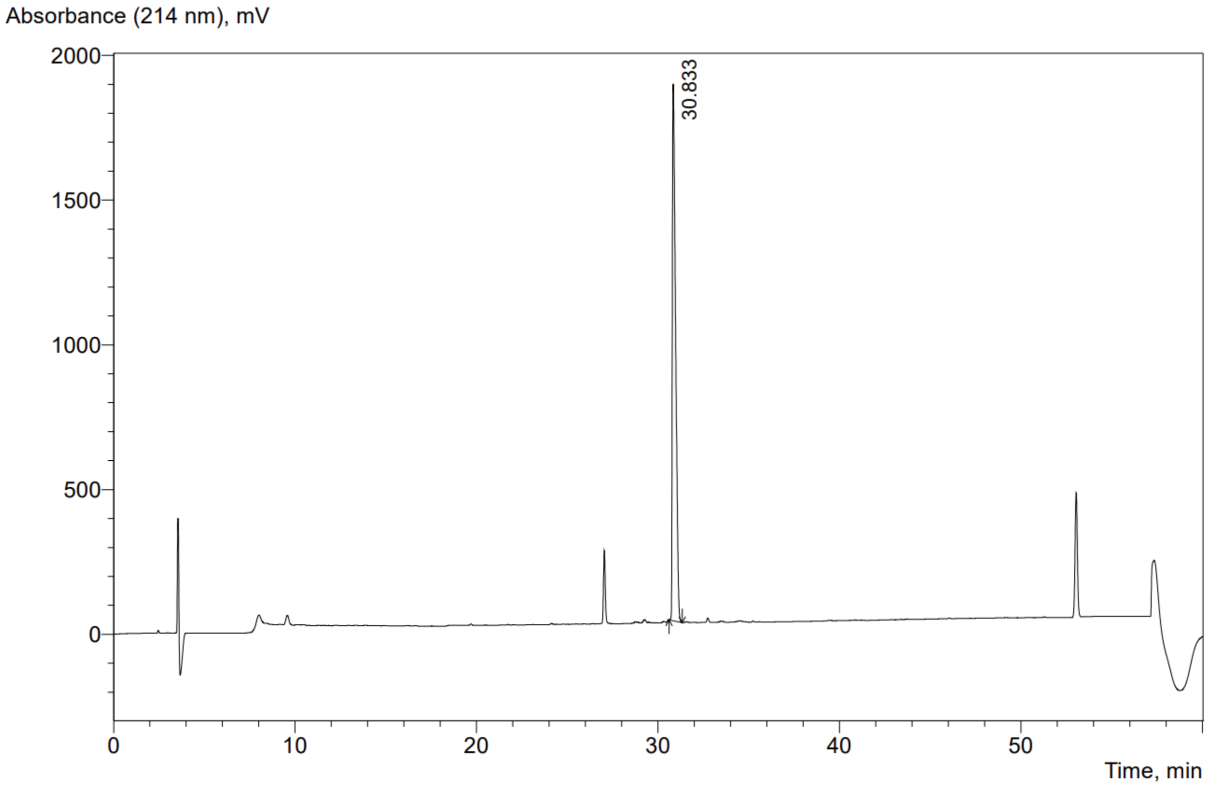

Figure S43. HPLC trace of the purified compound **8**.

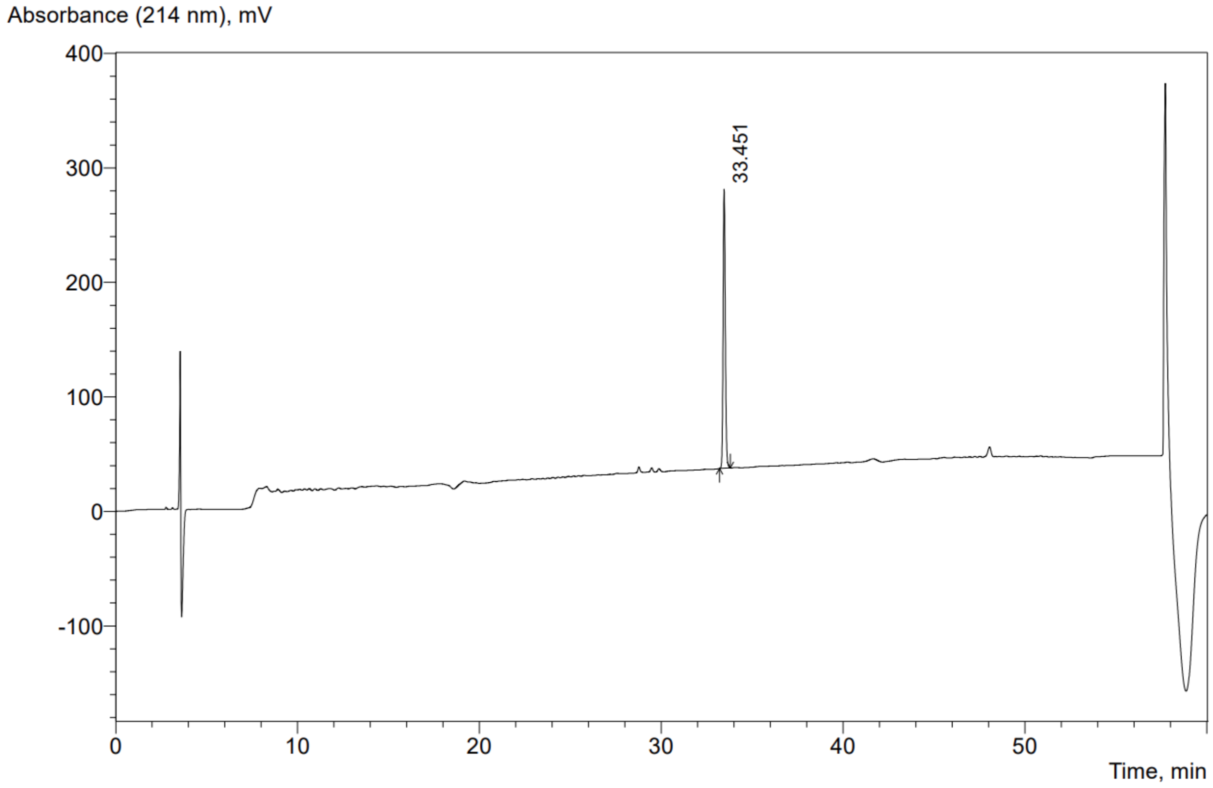

Figure S44. HPLC trace of the purified compound **9**.

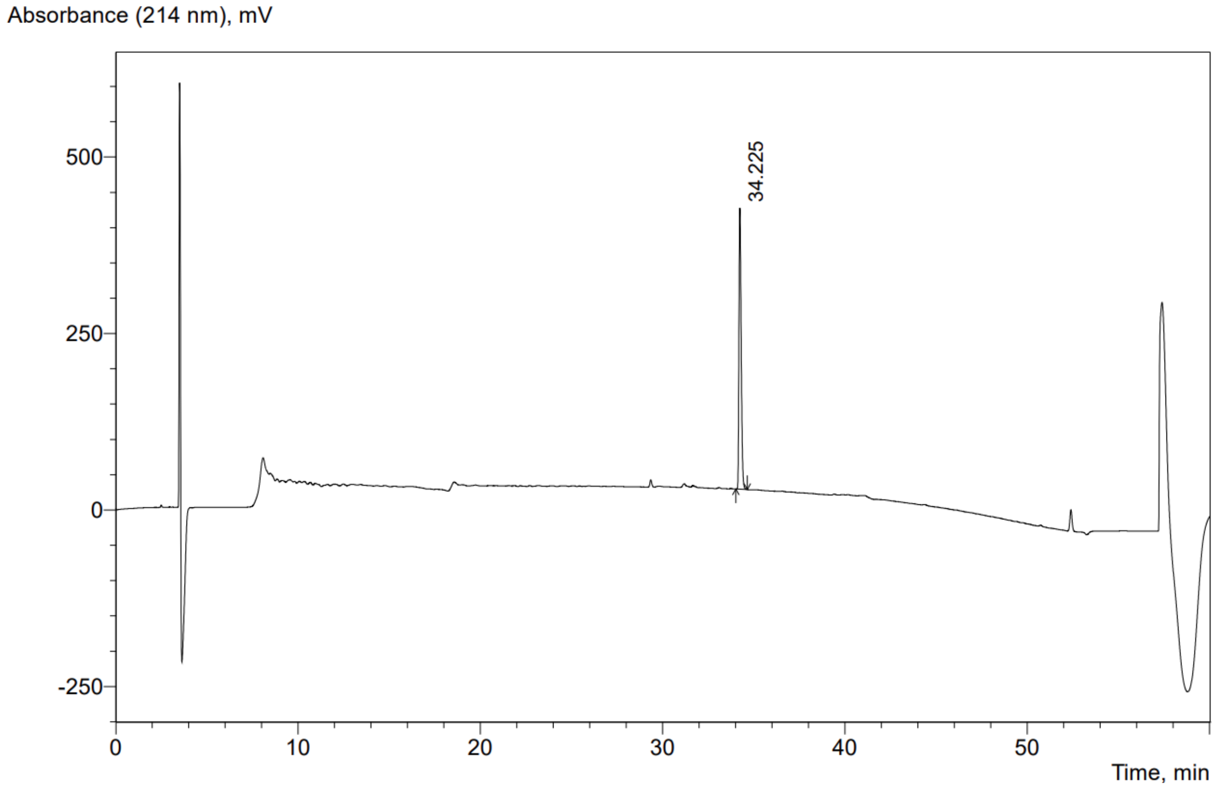

Figure S45. HPLC trace of the purified compound **10**.

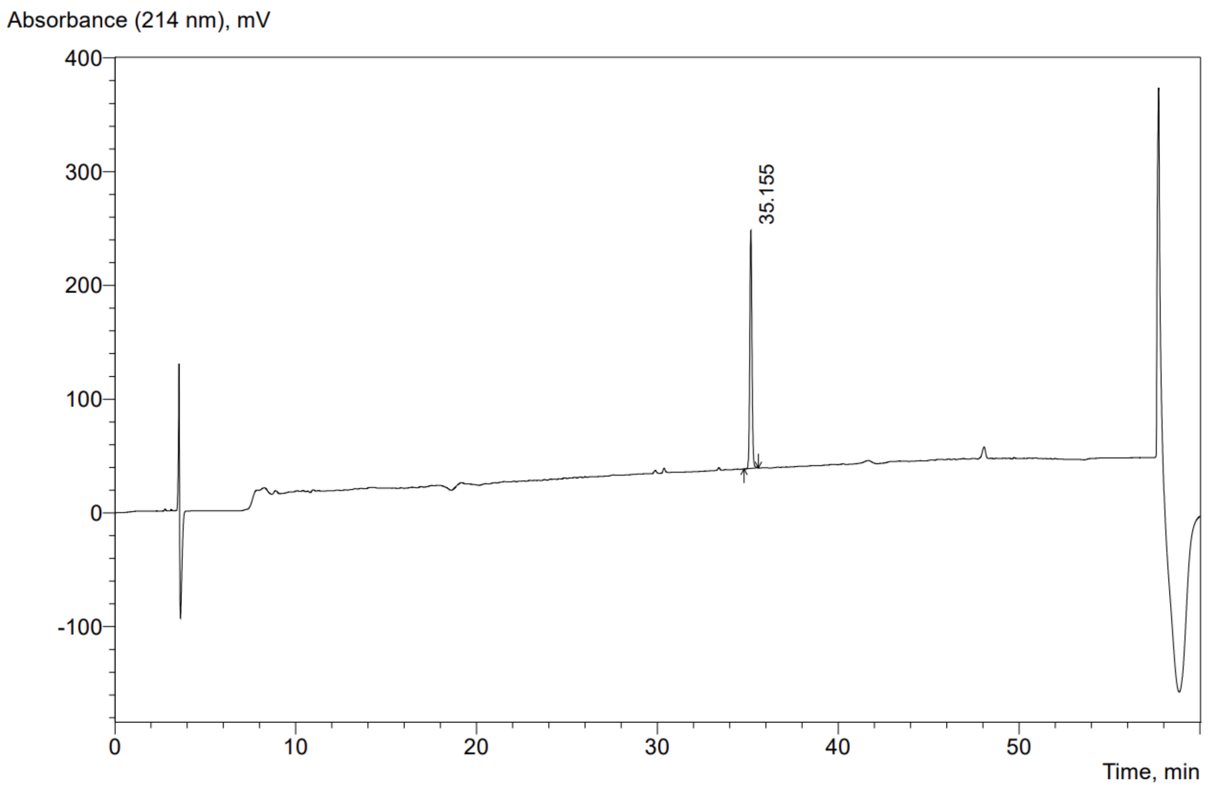

Figure S46. HPLC trace of the purified compound **11**.

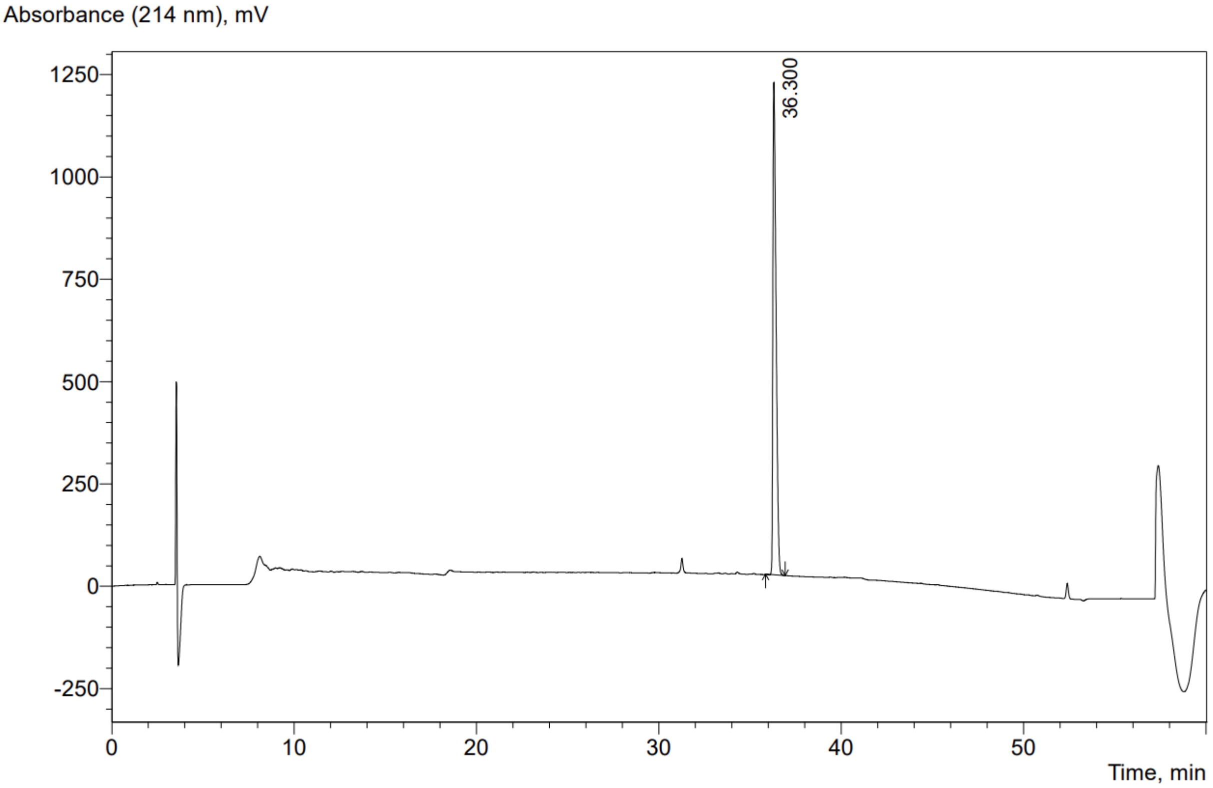

Figure S47. HPLC trace of the purified compound **12**.

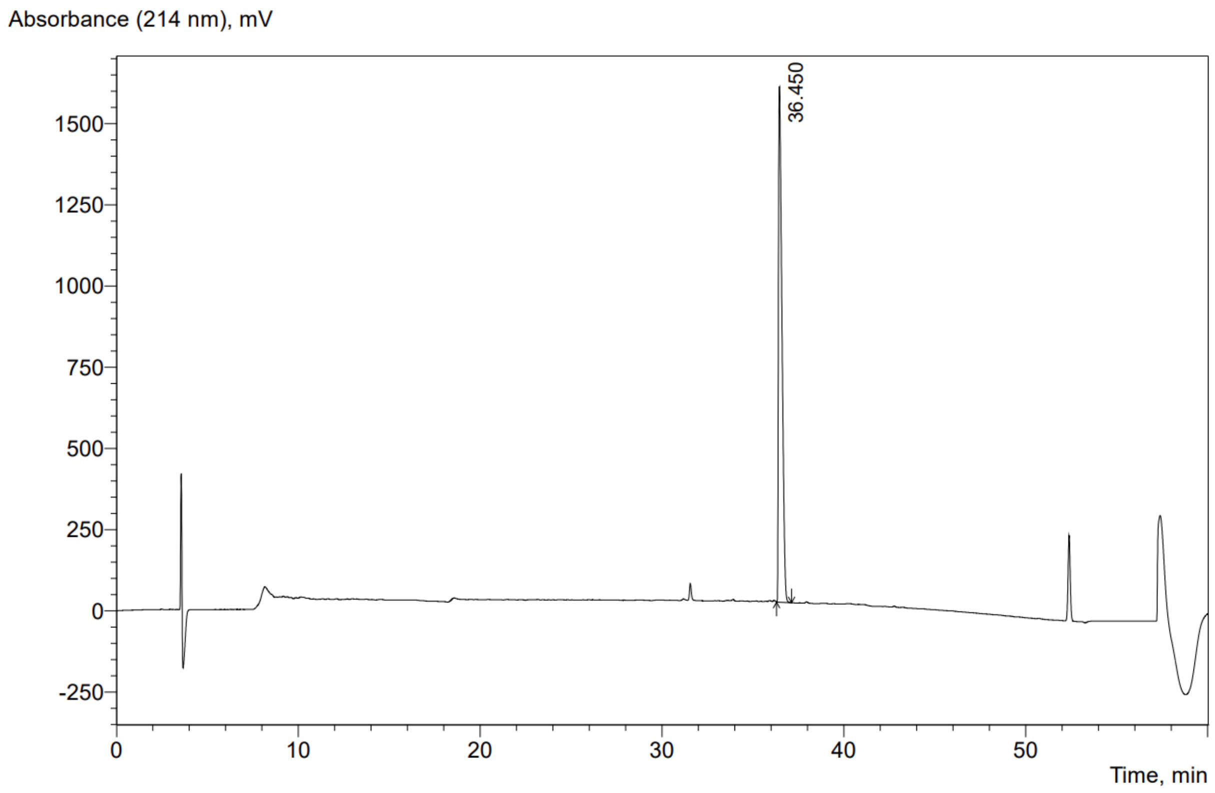

Figure S48. HPLC trace of the purified compound **21**.

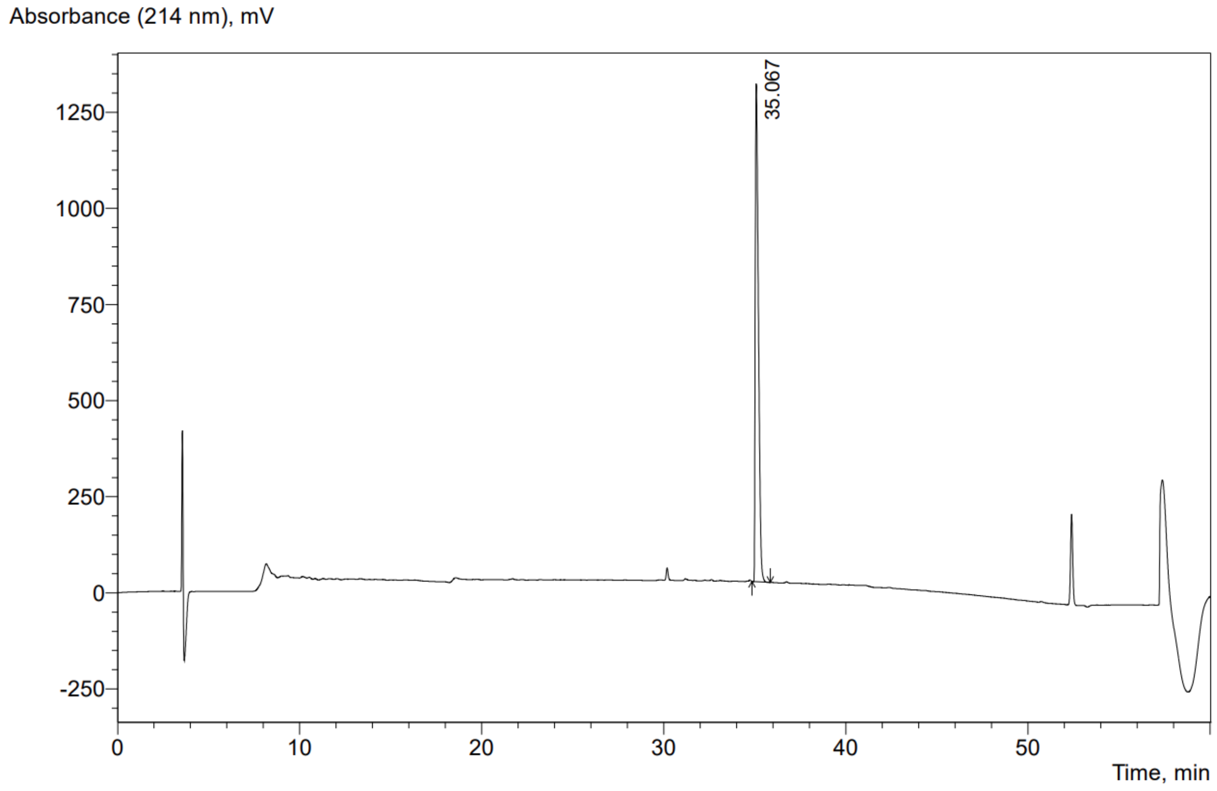

Figure S49. HPLC trace of the purified compound **22**.

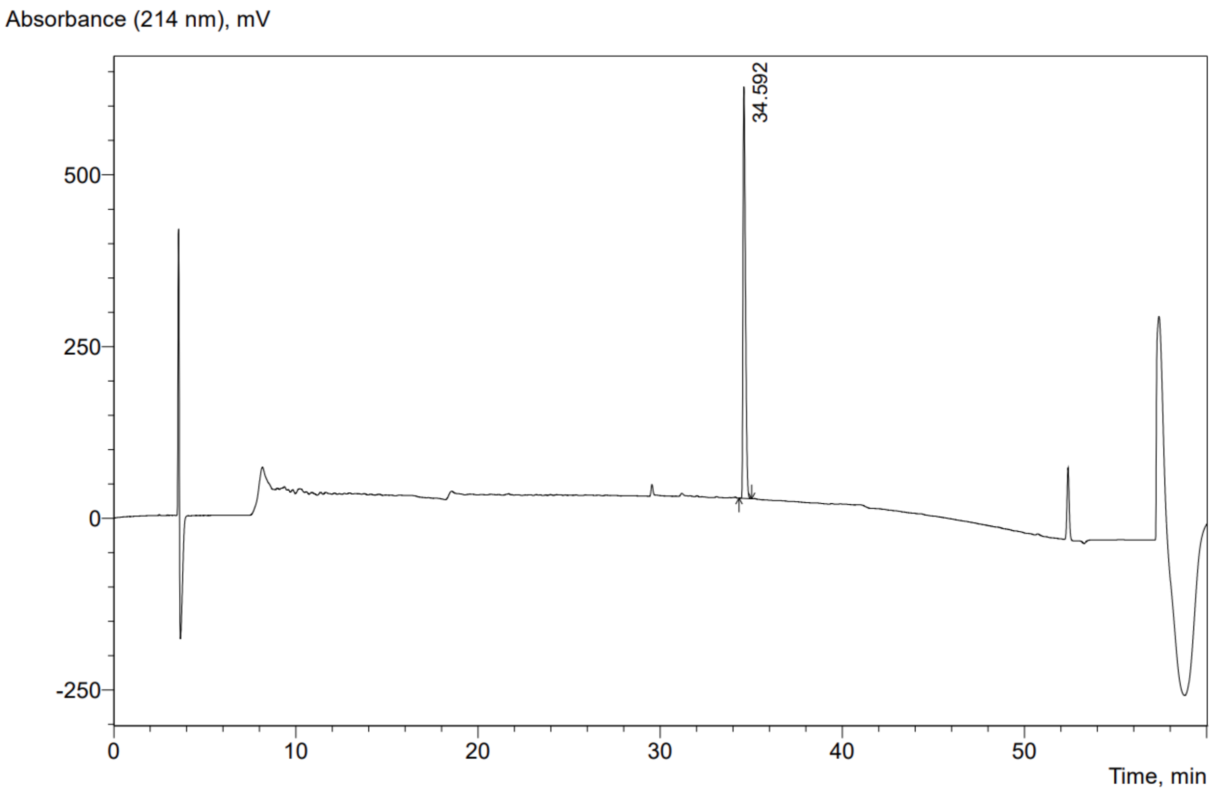

Figure S50. HPLC trace of the purified compound **23**.

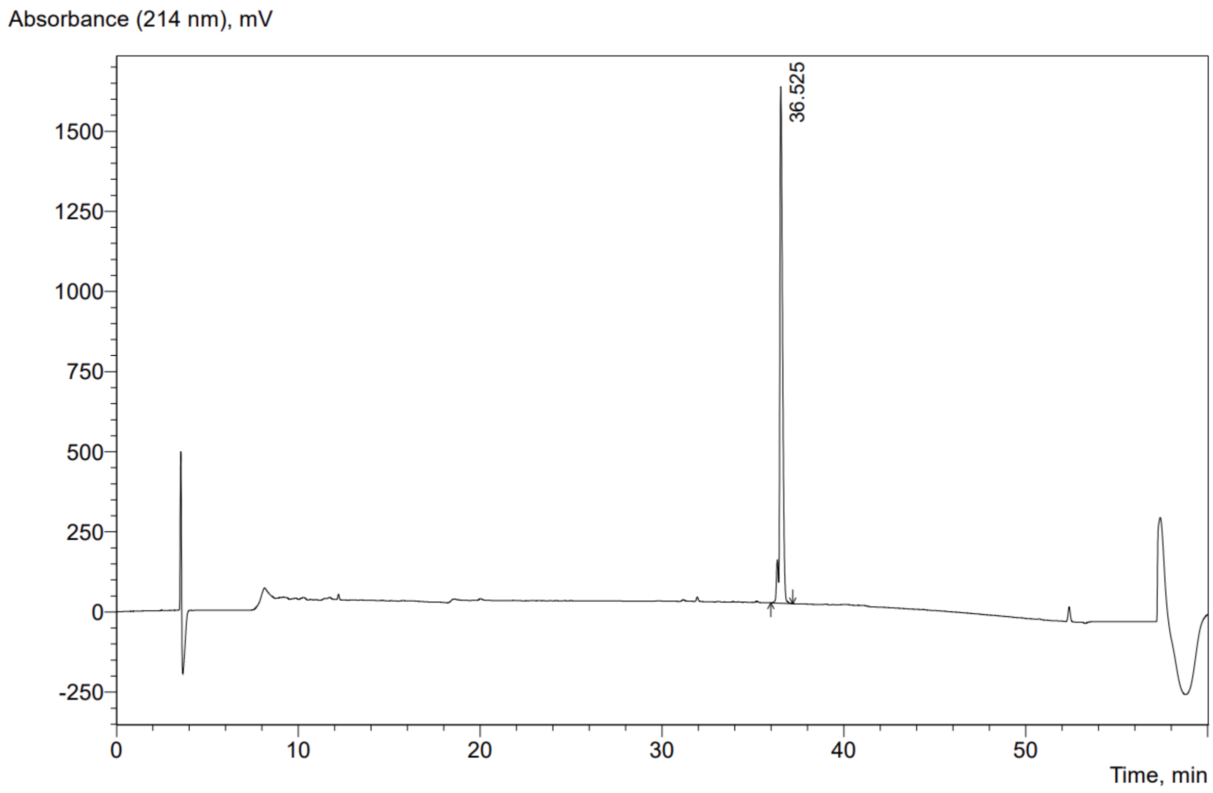

Figure S51. HPLC trace of the purified compound **33**.

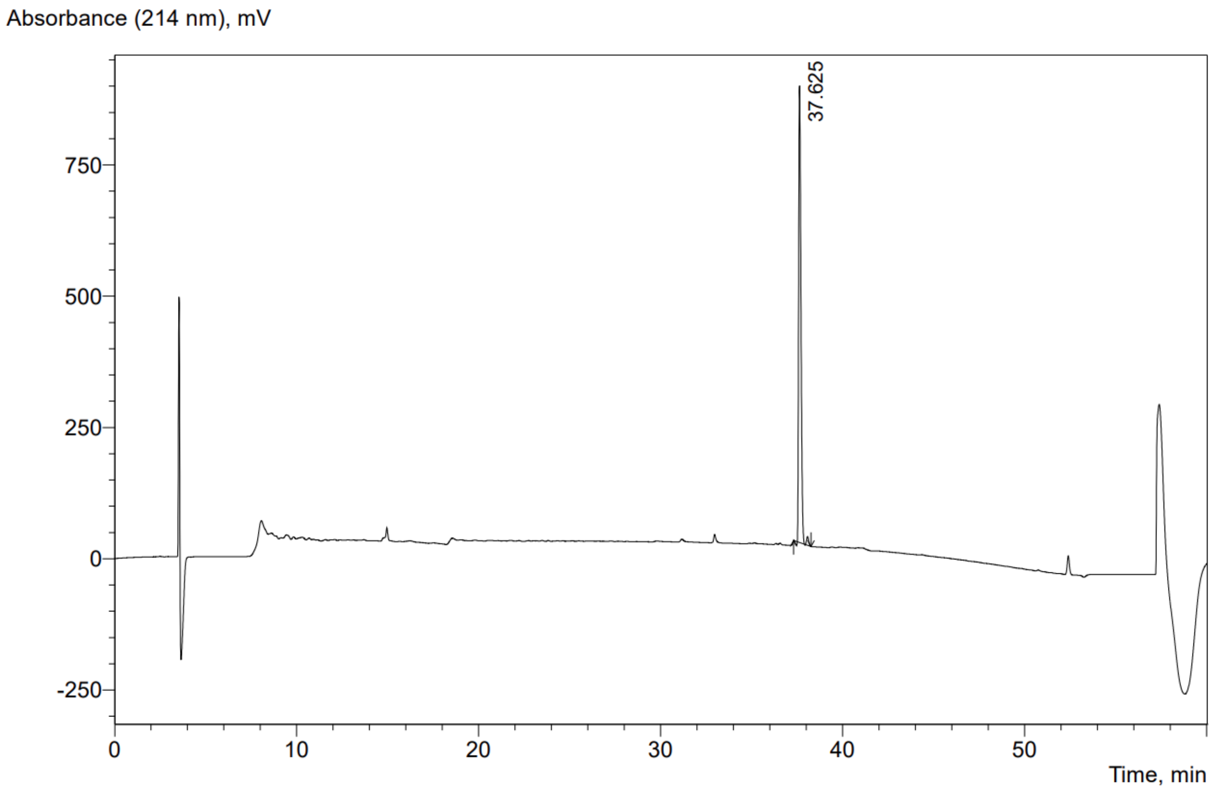

Figure S52. HPLC trace of the purified compound **34**.

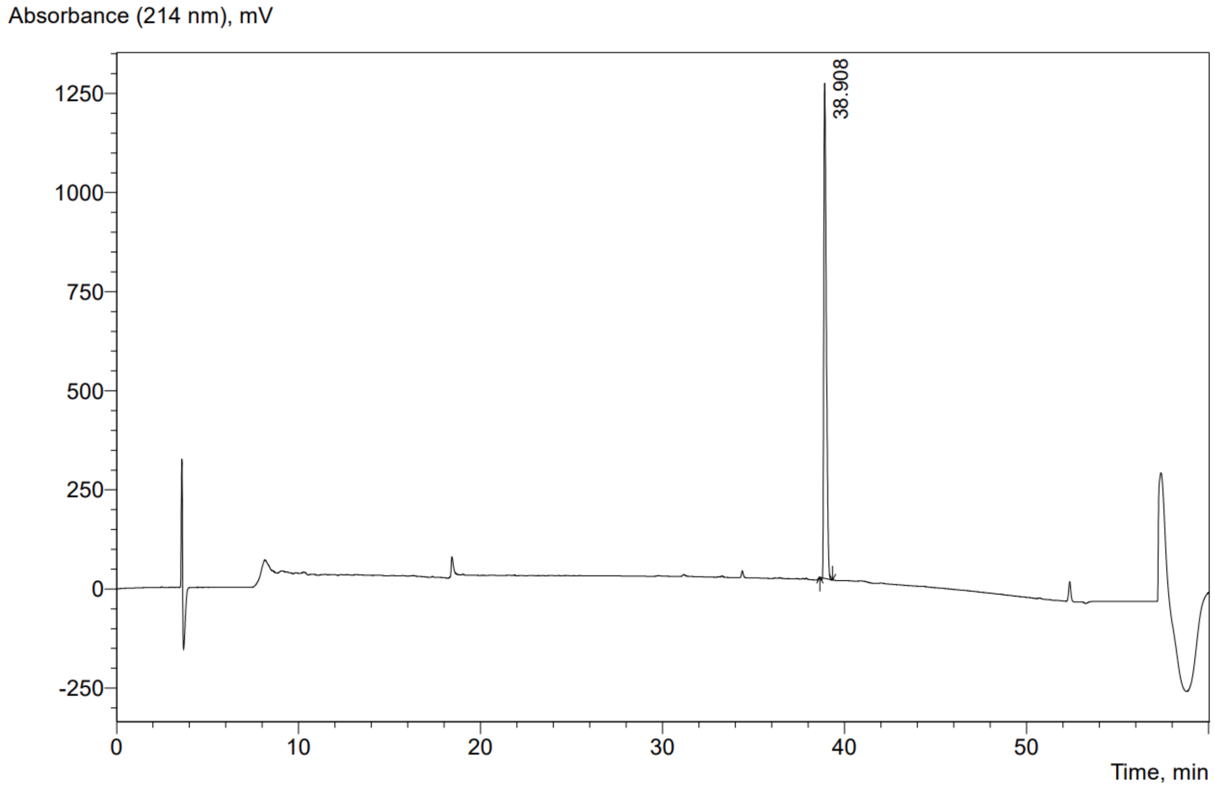

Figure S53. HPLC trace of the purified compound **35**.

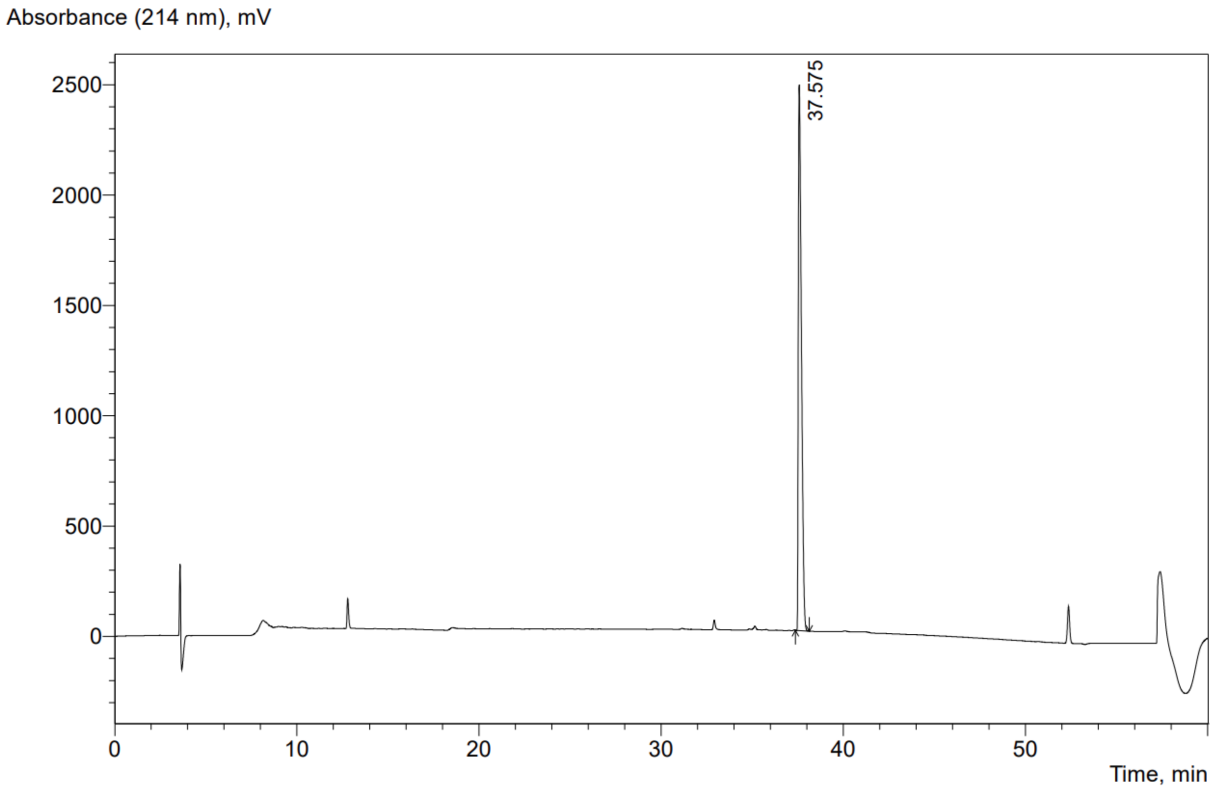

Figure S54. HPLC trace of the purified compound **36**.

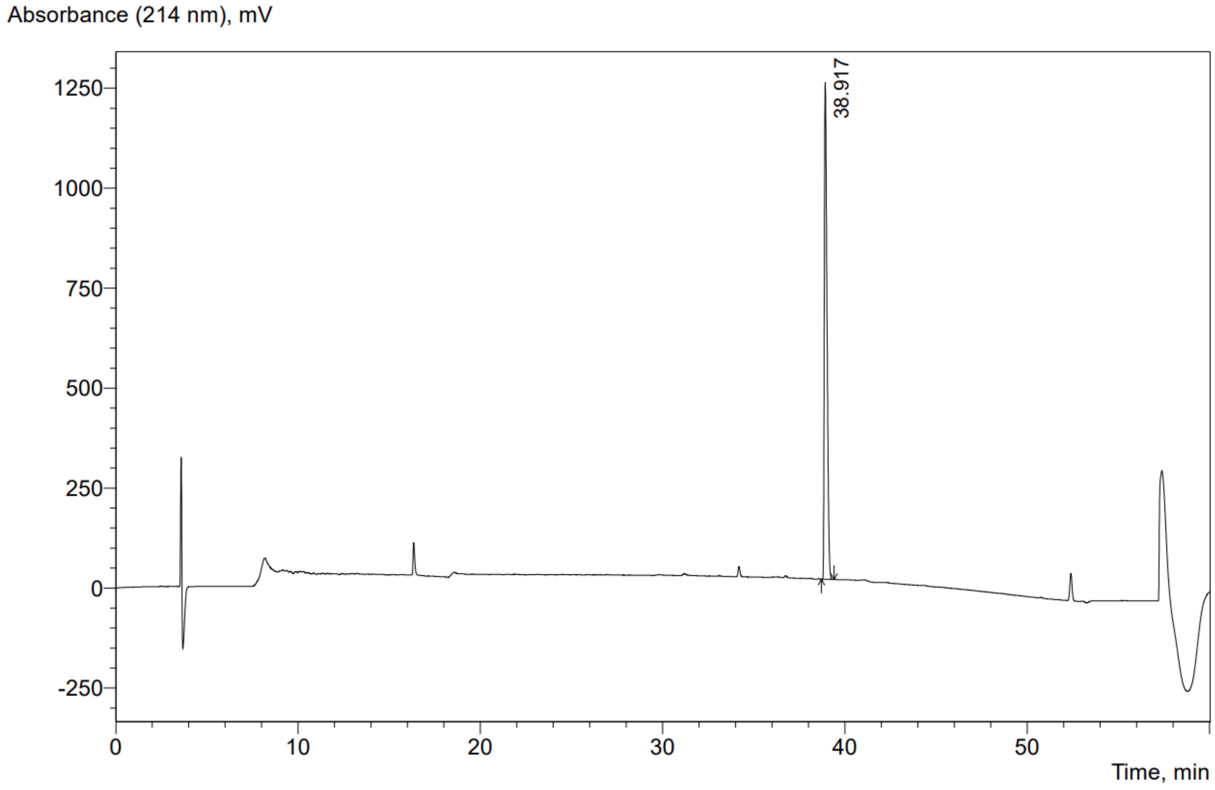

Figure S55. HPLC trace of the purified compound **37**.

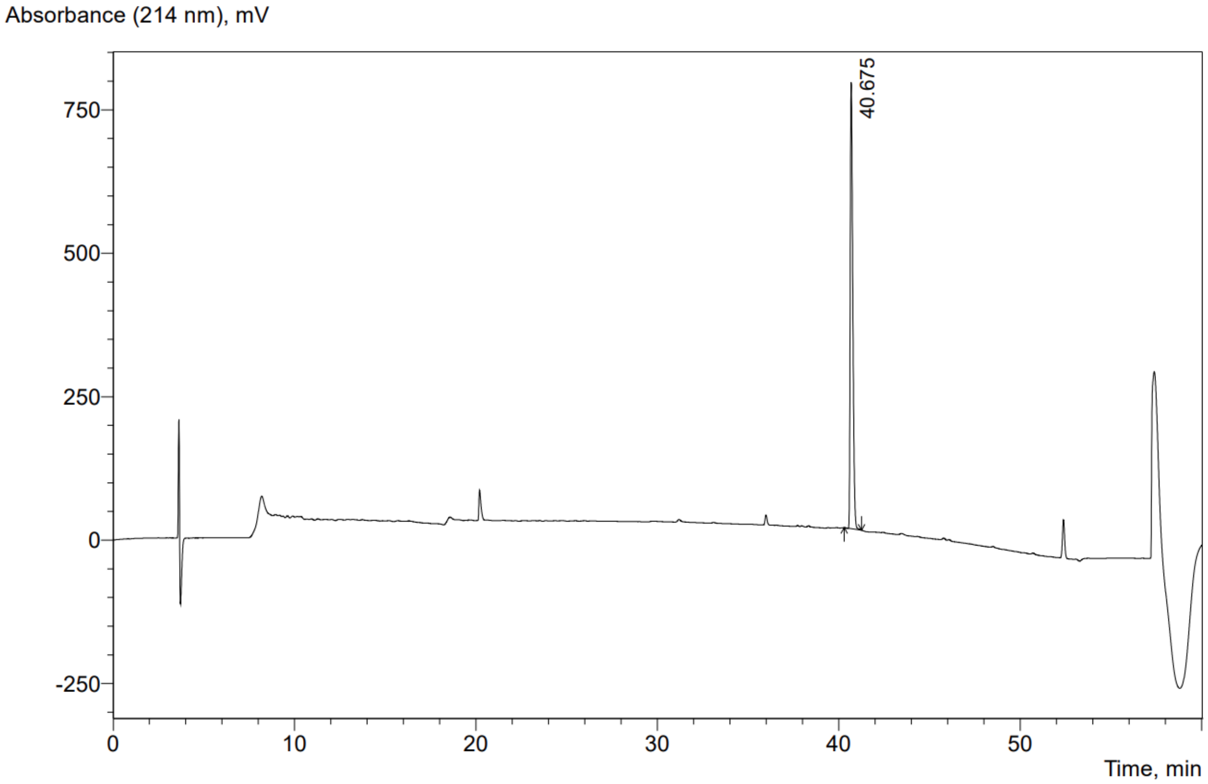

Figure S56. HPLC trace of the purified compound **38**.

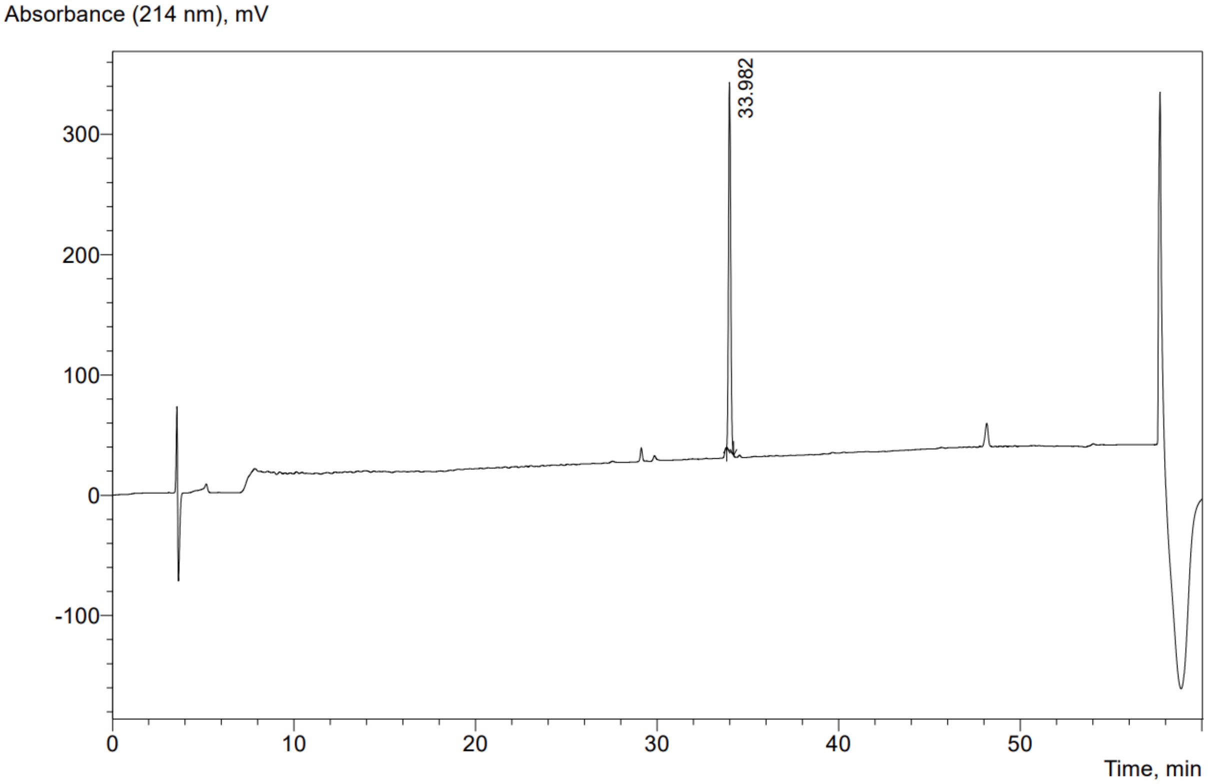

Figure S57. HPLC trace of the purified compound **39**.

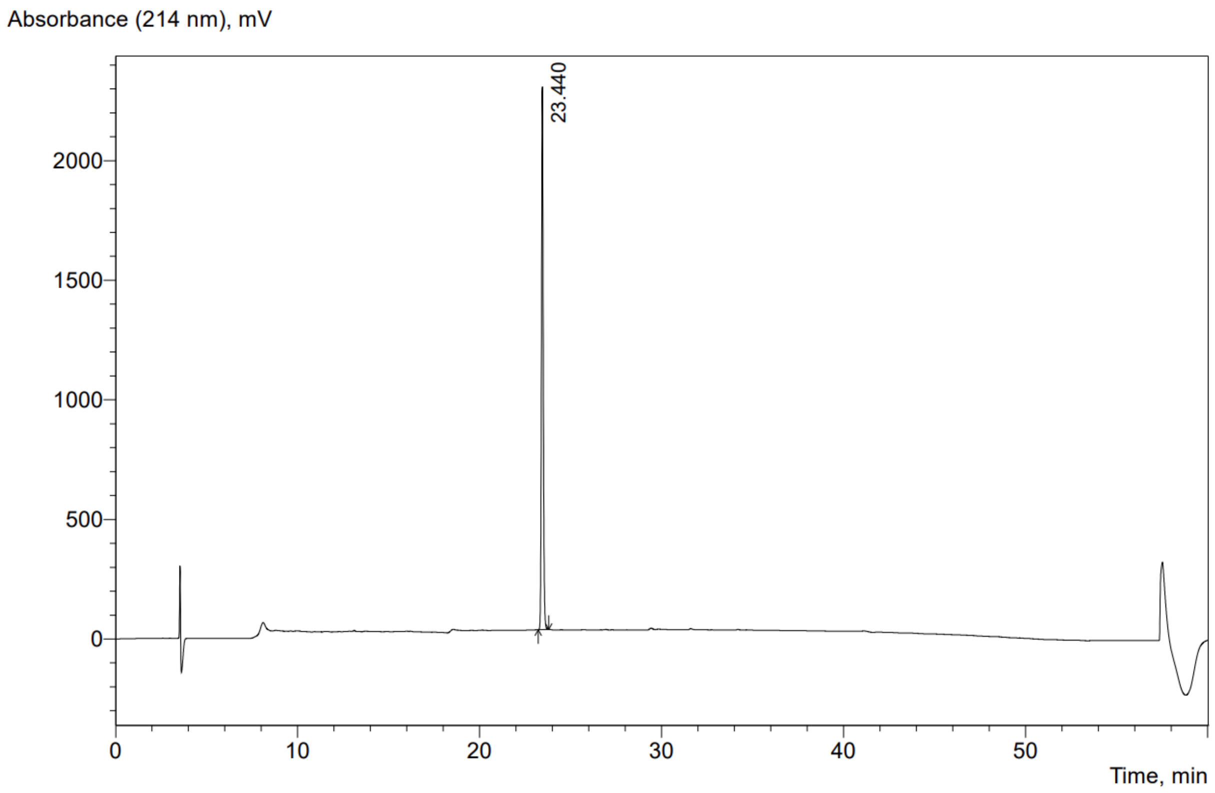

Figure S58. HPLC trace of the purified enterobactin.

References

(1) Cochrane, S. A.; Li, X.; He, S.; Yu, M.; Wu, M.; Vederas, J. C. Synthesis of Tridecaptin–Antibiotic Conjugates with in Vivo Activity against Gram-Negative Bacteria. *J Med Chem* **2015**, *58* (24), 9779–9785. https://doi.org/10.1021/acs.jmedchem.5b01578.

(2) Idowu, T.; Arthur, G.; Zhanel, G. G.; Schweizer, F. Heterodimeric Rifampicin–Tobramycin Conjugates Break Intrinsic Resistance of Pseudomonas Aeruginosa to Doxycycline and Chloramphenicol in Vitro and in a Galleria Mellonella in Vivo Model. *Eur J Med Chem* **2019**, *174*, 16–32. https://doi.org/10.1016/j.ejmech.2019.04.034.

(3) Schatz, C.; Louguet, S.; Le Meins, J.; Lecommandoux, S. Polysaccharide- *Block* -polypeptide Copolymer Vesicles: Towards Synthetic Viral Capsids. *Angewandte Chemie International Edition* **2009**, *48* (14), 2572–2575. https://doi.org/10.1002/anie.200805895.

(4) Vercillo, O. E.; Andrade, C. K. Z.; Wessjohann, L. A. Design and Synthesis of Cyclic RGD Pentapeptoids by Consecutive Ugi Reactions. *Org Lett* **2008**, *10* (2), 205–208. https://doi.org/10.1021/ol702521g.

(5) Hanna, J.; Allan, C.; Lawrence, C.; Meyer, O.; Wilson, N.; Hulme, A. Optimizing the Readout of Lanthanide-DOTA Complexes for the Detection of Ligand-Bound Copper(I). *Molecules* **2017**, *22* (5), 802. https://doi.org/10.3390/molecules22050802.

(6) Chang, D.; Zhu, D.; Shi, L. [3 + 2] Cycloadditions of Azides with Arynes via Photolysis of Phthaloyl Peroxide Derivatives. *J Org Chem* **2015**, *80* (11), 5928–5933. https://doi.org/10.1021/acs.joc.5b00517.

(7) Gann, A. W.; Amoroso, J. W.; Einck, V. J.; Rice, W. P.; Chambers, J. J.; Schnarr, N. A. A Photoinduced, Benzyne Click Reaction. *Org Lett* **2014**, *16* (7), 2003–2005. https://doi.org/10.1021/ol500389t.

(8) Antoni, P.; Hed, Y.; Nordberg, A.; Nyström, D.; von Holst, H.; Hult, A.; Malkoch, M. Bifunctional Dendrimers: From Robust Synthesis and Accelerated One-Pot Postfunctionalization Strategy to Potential Applications. *Angewandte Chemie International Edition* **2009**, *48* (12), 2126–2130. https://doi.org/10.1002/anie.200804987.

(9) Rossetti, A.; Sacchetti, A.; Meneghetti, F.; Colombo Dugoni, G.; Mori, M.; Castellano, C. Synthesis and Characterization of New Triazole-Bispidinone Scaffolds and Their Metal Complexes for Catalytic Applications. *Molecules* **2023**, *28* (17), 6351. https://doi.org/10.3390/molecules28176351.

(10) Macerata, E.; Mossini, E.; Scaravaggi, S.; Mariani, M.; Mele, A.; Panzeri, W.; Boubals, N.; Berthon, L.; Charbonnel, M.-C.; Sansone, F.; Arduini, A.; Casnati, A. Hydrophilic Clicked 2,6-Bis-Triazolyl-Pyridines Endowed with High Actinide Selectivity and Radiochemical Stability: Toward a Closed Nuclear Fuel Cycle. *J Am Chem Soc* **2016**, *138* (23), 7232–7235. https://doi.org/10.1021/jacs.6b03106.

(11) Post, E. A. J.; Fletcher, S. P. Controlling the Kinetics of Self-Reproducing Micelles by Catalyst Compartmentalization in a Biphasic System. *J Org Chem* **2019**, *84* (5), 2741–2755. https://doi.org/10.1021/acs.joc.8b03149.

(12) Gracias, V.; Frank, K. E.; Milligan, G. L.; Aubé, J. Ring Expansion by in Situ Tethering of Hydroxy Azides to Ketones: The Boyer Reaction. *Tetrahedron* **1997**, *53* (48), 16241–16252. https://doi.org/10.1016/S0040-4020(97)01012-0.

(13) Chen, Y.-F.; Wu, C.-H.; Chen, L.-H.; Lee, H.-W.; Lee, J.-C.; Yeh, T.-K.; Chang, J.-Y.; Chou, M.-C.; Wu, H.-L.; Lai, Y.-P.; Song, J.-S.; Yeh, K.-C.; Chen, C.-T.; Lee, C.-J.; Shia, K.-S.; Shen, M.-R. Discovery of Potential Neuroprotective Agents against Paclitaxel-Induced Peripheral Neuropathy. *J Med Chem* **2022**, *65* (6), 4767–4782. https://doi.org/10.1021/acs.jmedchem.1c01912.

(14) Zhang, J.; Jiang, J.; Li, Y.; Wan, X. Iodide-Catalyzed Synthesis of *N* -Nitrosamines via C–N Cleavage of Nitromethane. *J Org Chem* **2013**, *78* (22), 11366–11372. https://doi.org/10.1021/jo401915t.

(15) Baramov, T.; Keijzer, K.; Irran, E.; Mösker, E.; Baik, M.; Süssmuth, R. Synthesis and Structural Characterization of Hexacoordinate Silicon, Germanium, and Titanium Complexes of the *E. Coli* Siderophore Enterobactin. *Chemistry – A European Journal* **2013**, *19* (32), 10536–10542. https://doi.org/10.1002/chem.201301825.

(16) Ramirez, R. J. A.; Karamanukyan, L.; Ortiz, S.; Gutierrez, C. G. A Much Improved Synthesis of the Siderophore Enterobactin. *Tetrahedron Lett* **1997**, *38* (5), 749–752. https://doi.org/10.1016/S0040-4039(96)02452-5.

(17) Shanzer, A.; Libman, J. Total Synthesis of Enterobactin via an Organotin Template. *J Chem Soc Chem Commun* **1983**, No. 15, 846. https://doi.org/10.1039/c39830000846.

(18) Marinez, E. R.; Salmassian, E. K.; Lau, T. T.; Gutierrez, C. G. Enterobactin and Enantioenterobactin. *J Org Chem* **1996**, *61* (10), 3548–3550. https://doi.org/10.1021/jo9520194.
